# Supplementary figures and images for: The combined effects of temperature and relative humidity parameters on the reproduction of Stomoxys species in a laboratory setting
Source: PLoS One. 2020 Dec 21;15(12):e0242794. doi: 10.1371/journal.pone.0242794 (PMC7751869; doi:10.1371/journal.pone.0242794)

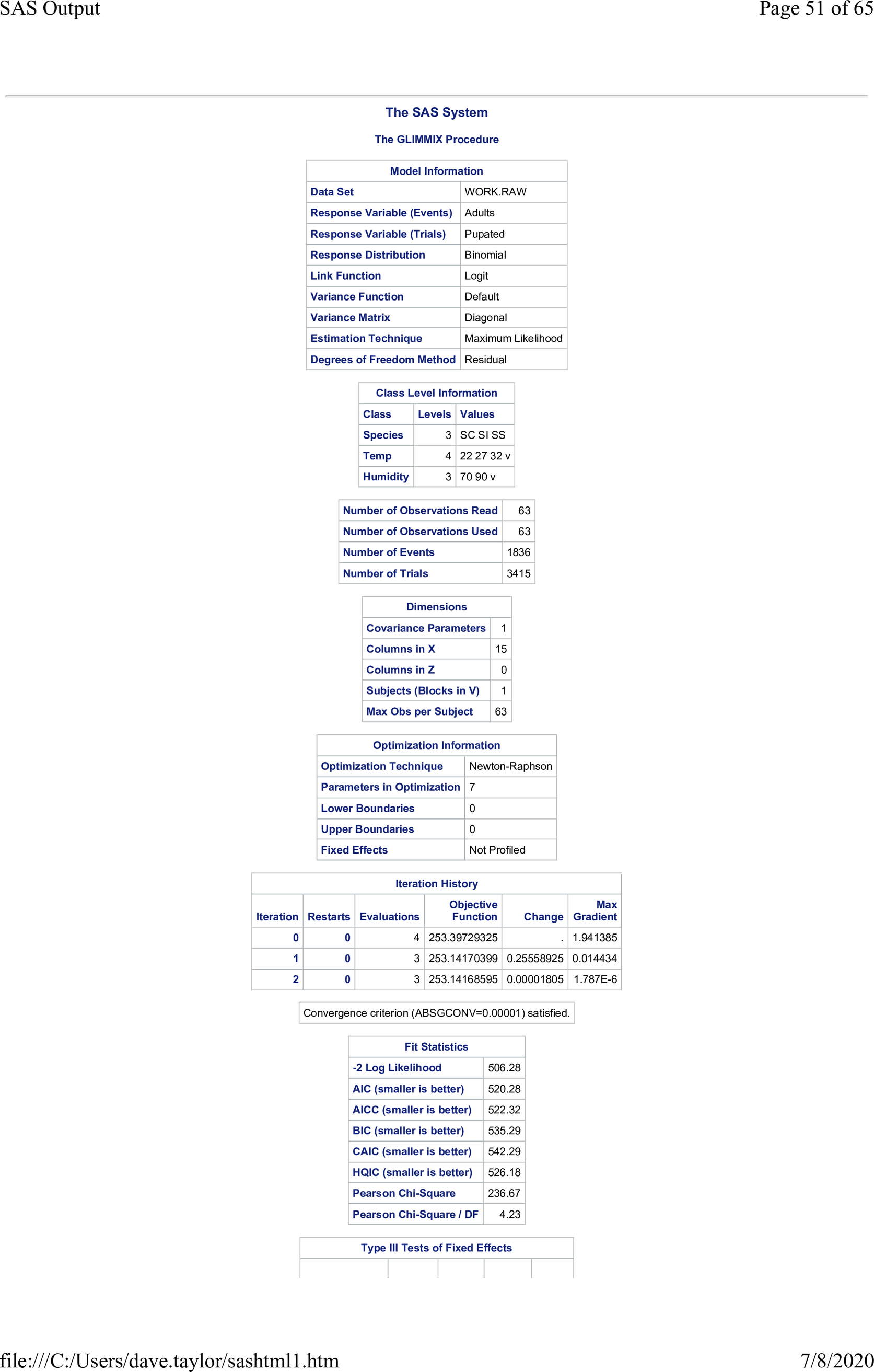

Supplement: S1 File — (ZIP) [file pone.0242794.s001.zip › PACE Corrected/S1_File.pdf.tif]

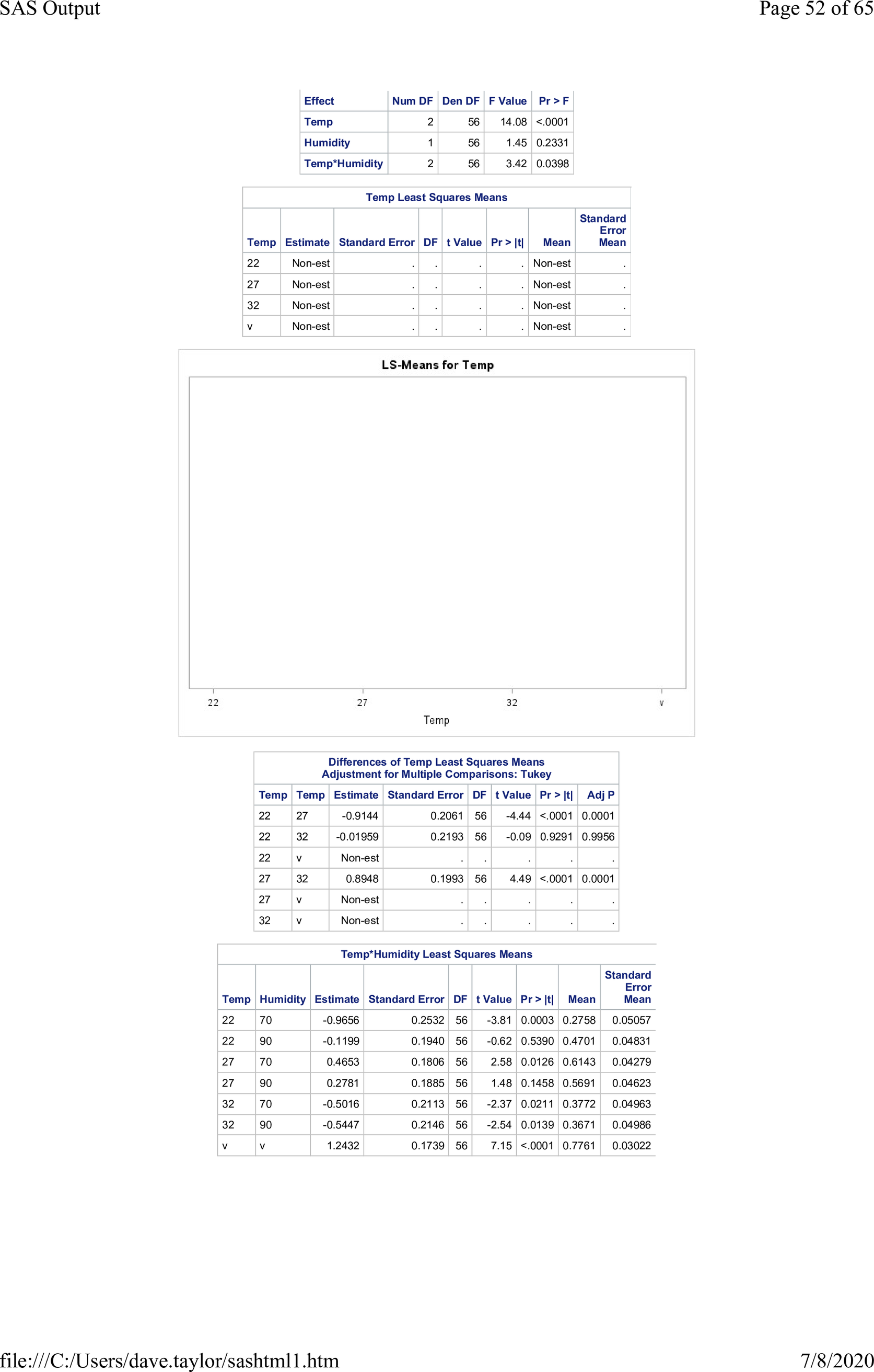

Supplement: S1 File — (ZIP) [file pone.0242794.s001.zip › PACE Corrected/S1_File.pdf.tif]

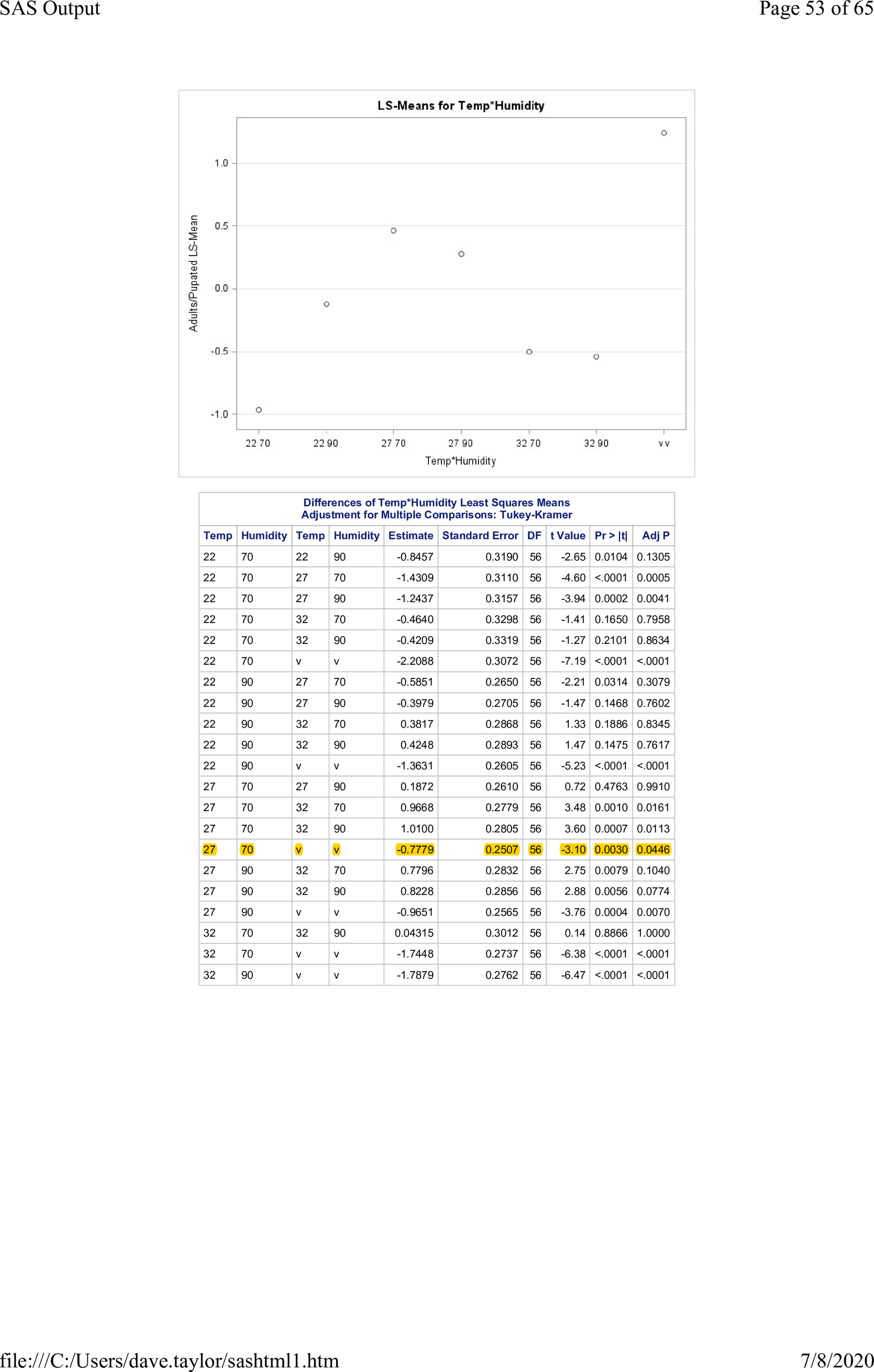

Supplement: S1 File — (ZIP) [file pone.0242794.s001.zip › PACE Corrected/S1_File.pdf.tif]

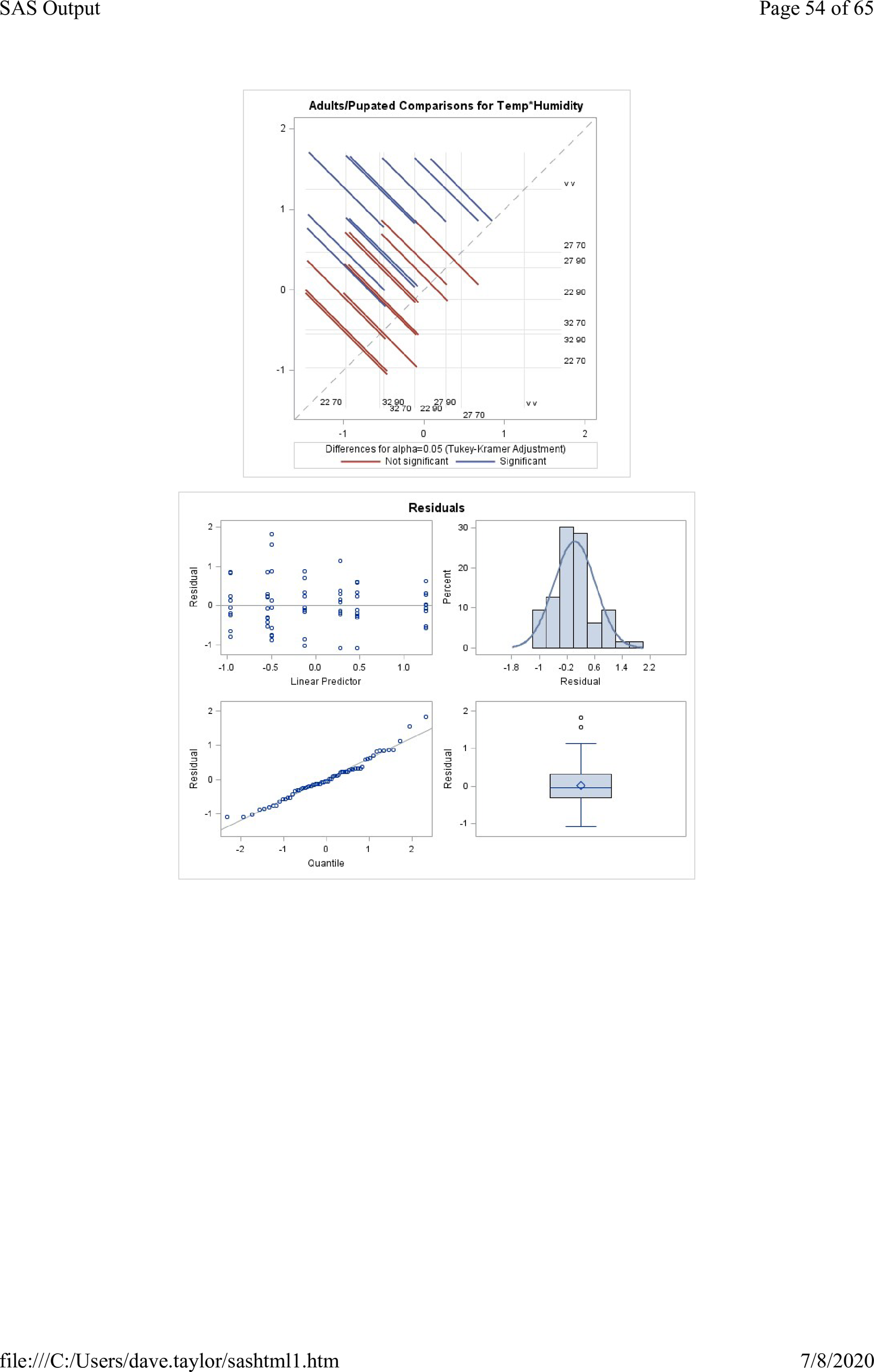

Supplement: S1 File — (ZIP) [file pone.0242794.s001.zip › PACE Corrected/S1_File.pdf.tif]

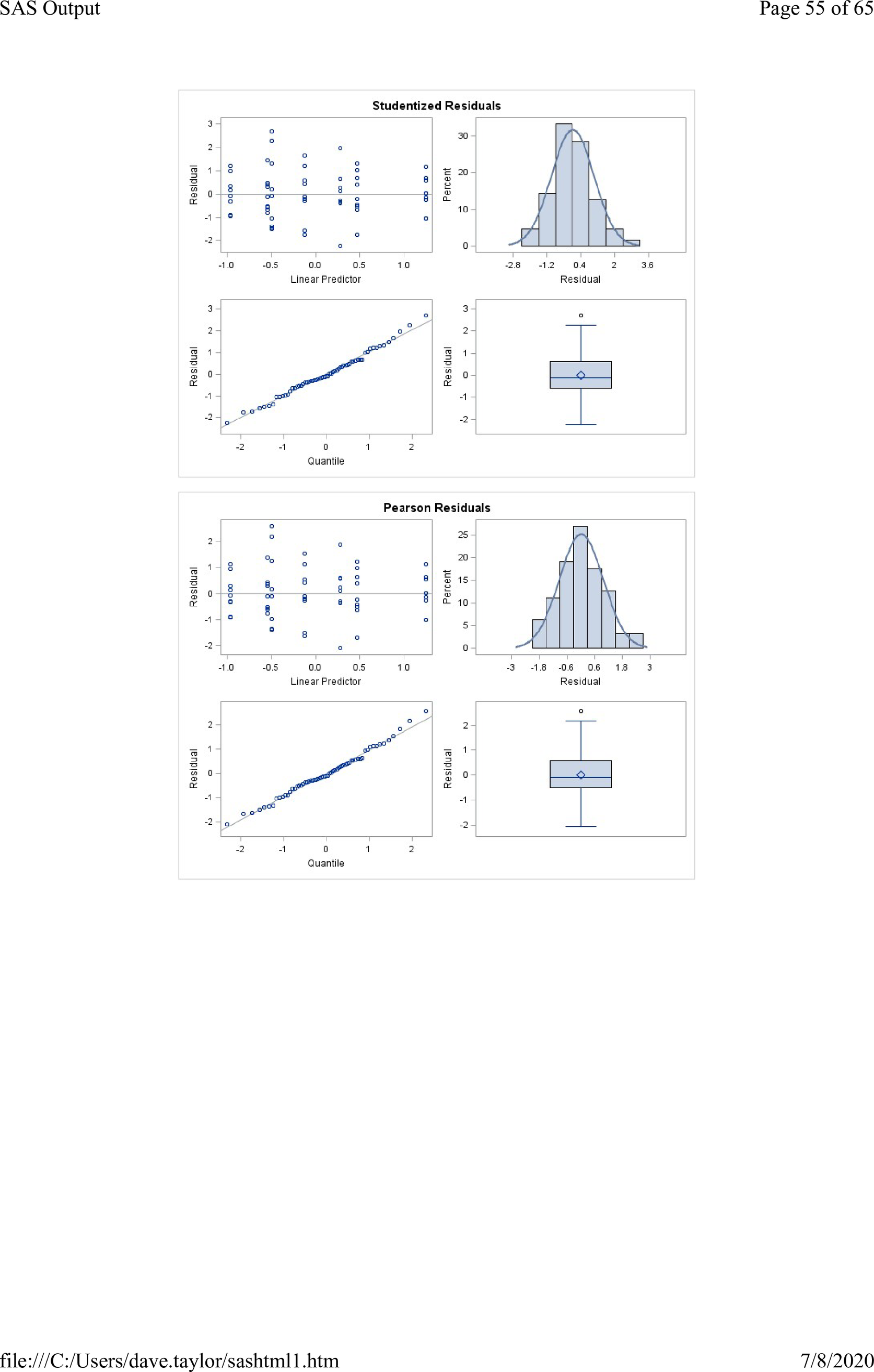

Supplement: S1 File — (ZIP) [file pone.0242794.s001.zip › PACE Corrected/S1_File.pdf.tif]

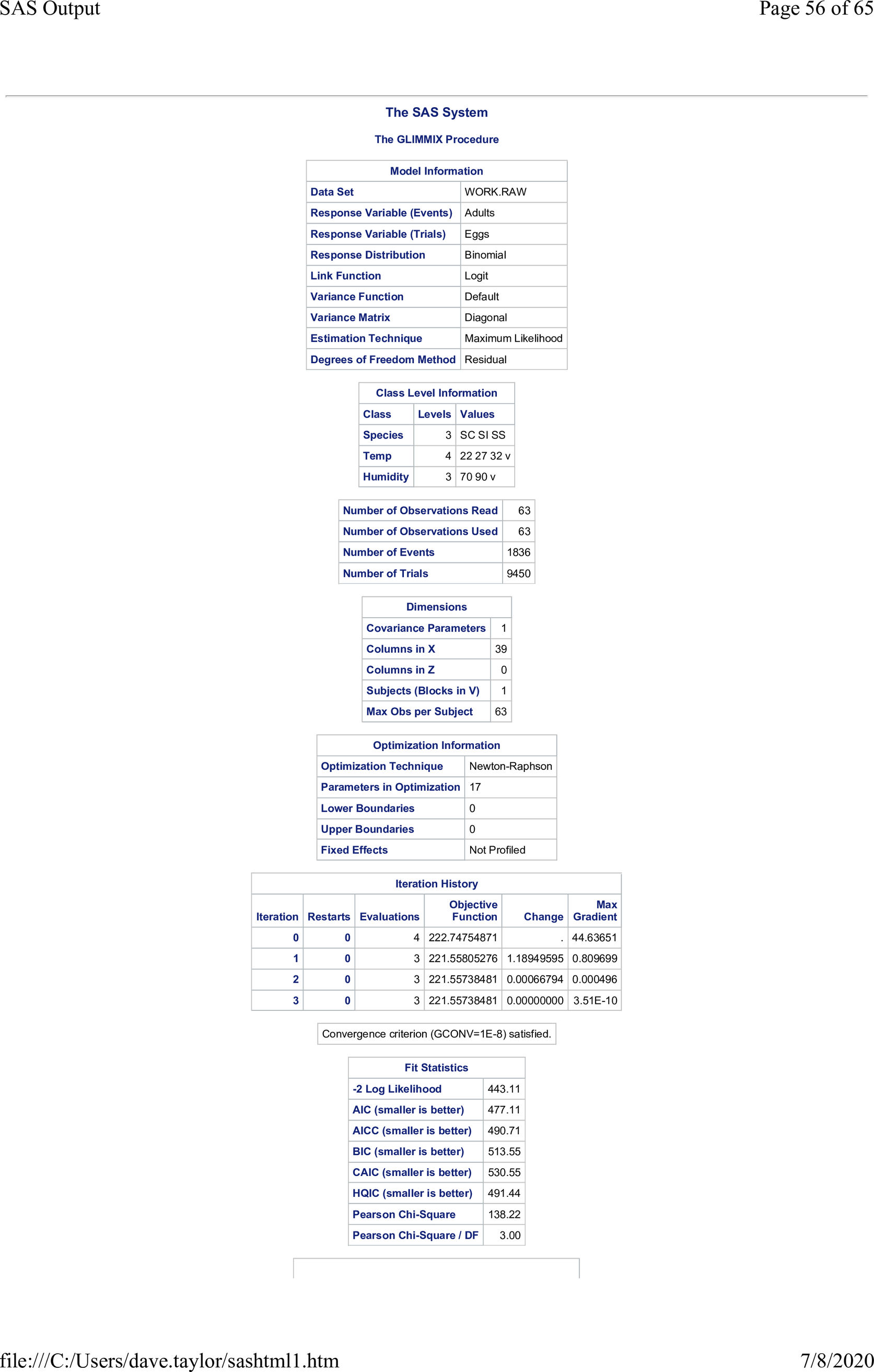

Supplement: S1 File — (ZIP) [file pone.0242794.s001.zip › PACE Corrected/S1_File.pdf.tif]

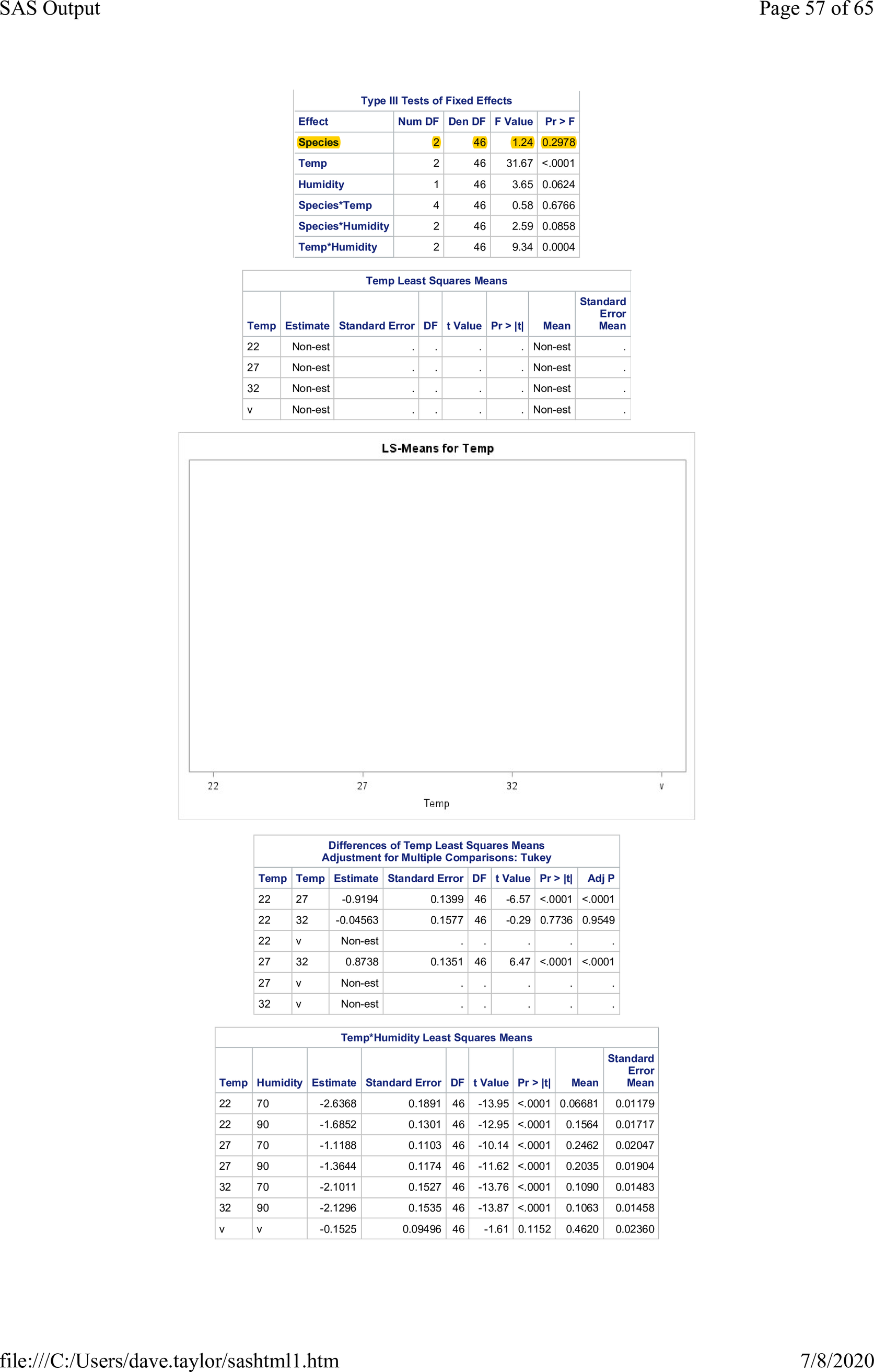

Supplement: S1 File — (ZIP) [file pone.0242794.s001.zip › PACE Corrected/S1_File.pdf.tif]

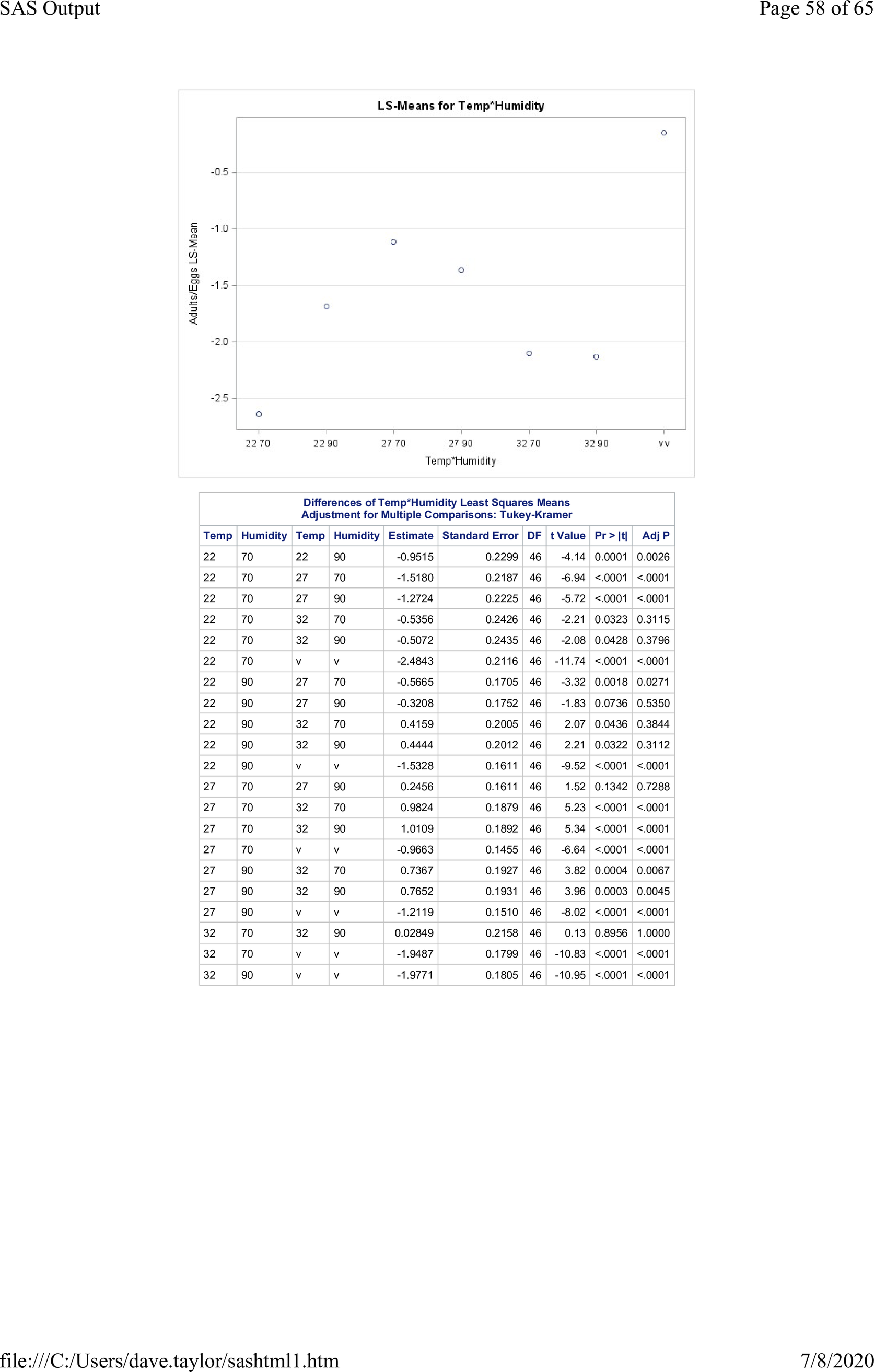

Supplement: S1 File — (ZIP) [file pone.0242794.s001.zip › PACE Corrected/S1_File.pdf.tif]

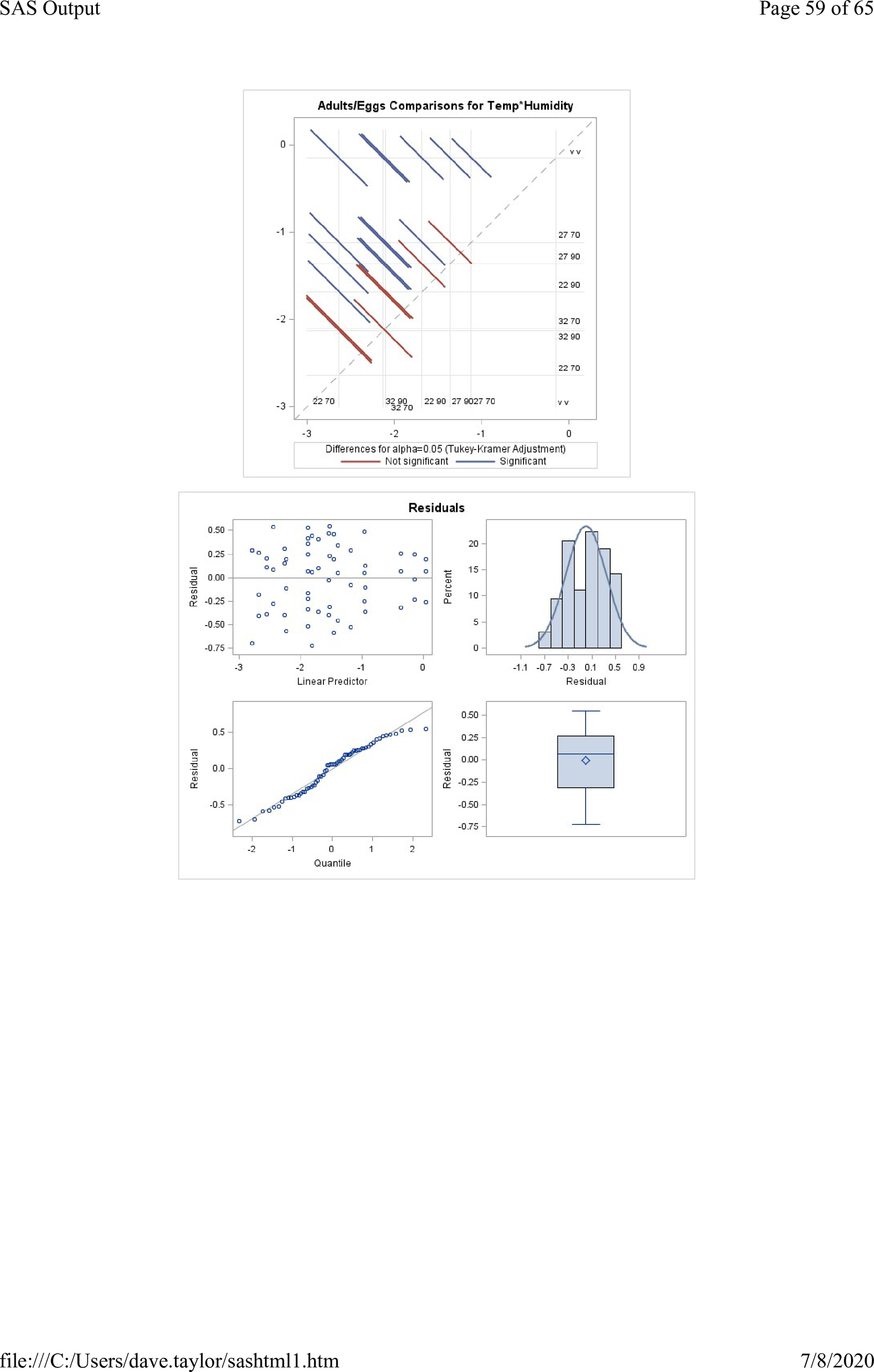

Supplement: S1 File — (ZIP) [file pone.0242794.s001.zip › PACE Corrected/S1_File.pdf.tif]

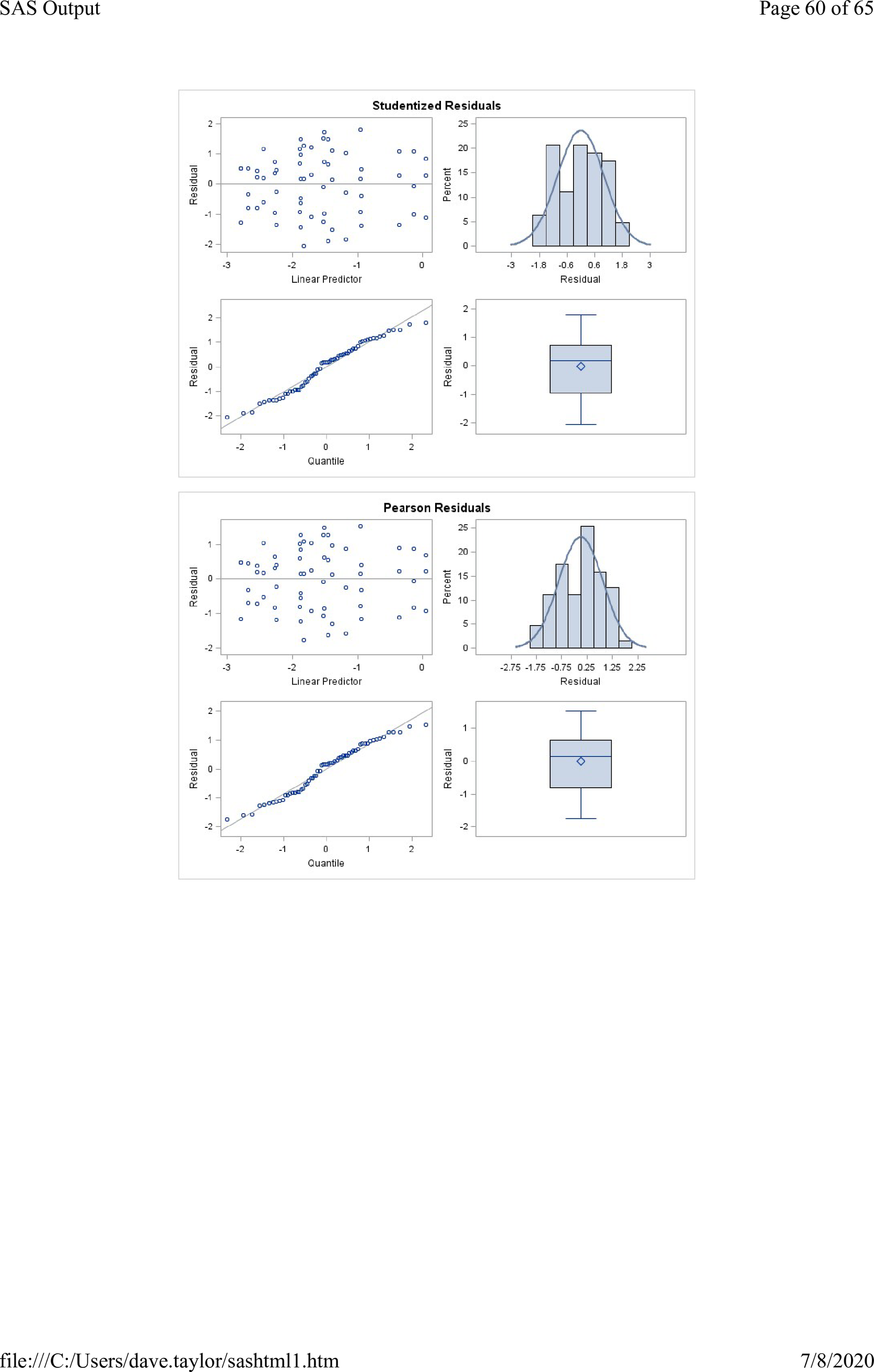

Supplement: S1 File — (ZIP) [file pone.0242794.s001.zip › PACE Corrected/S1_File.pdf.tif]

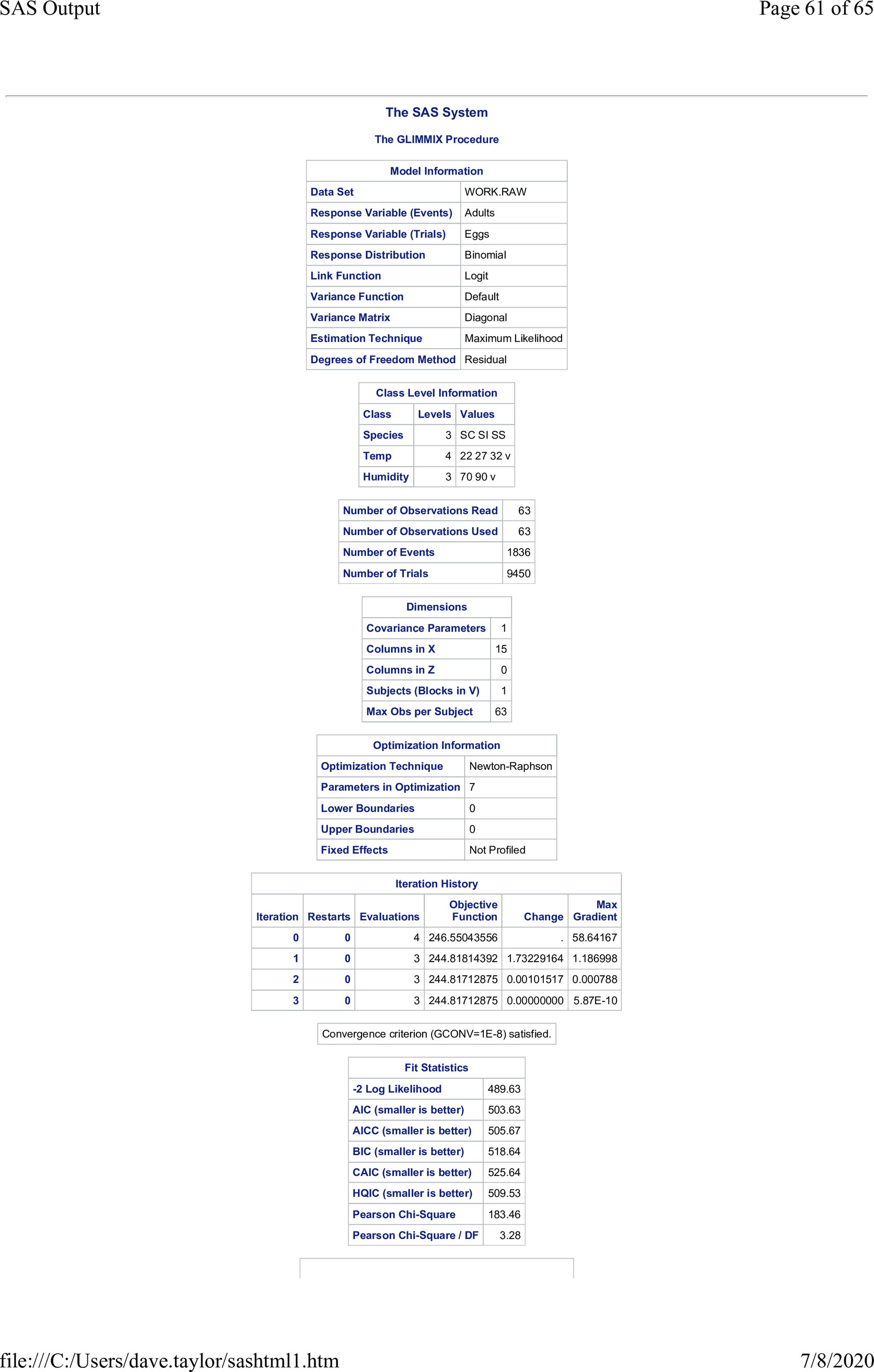

Supplement: S1 File — (ZIP) [file pone.0242794.s001.zip › PACE Corrected/S1_File.pdf.tif]

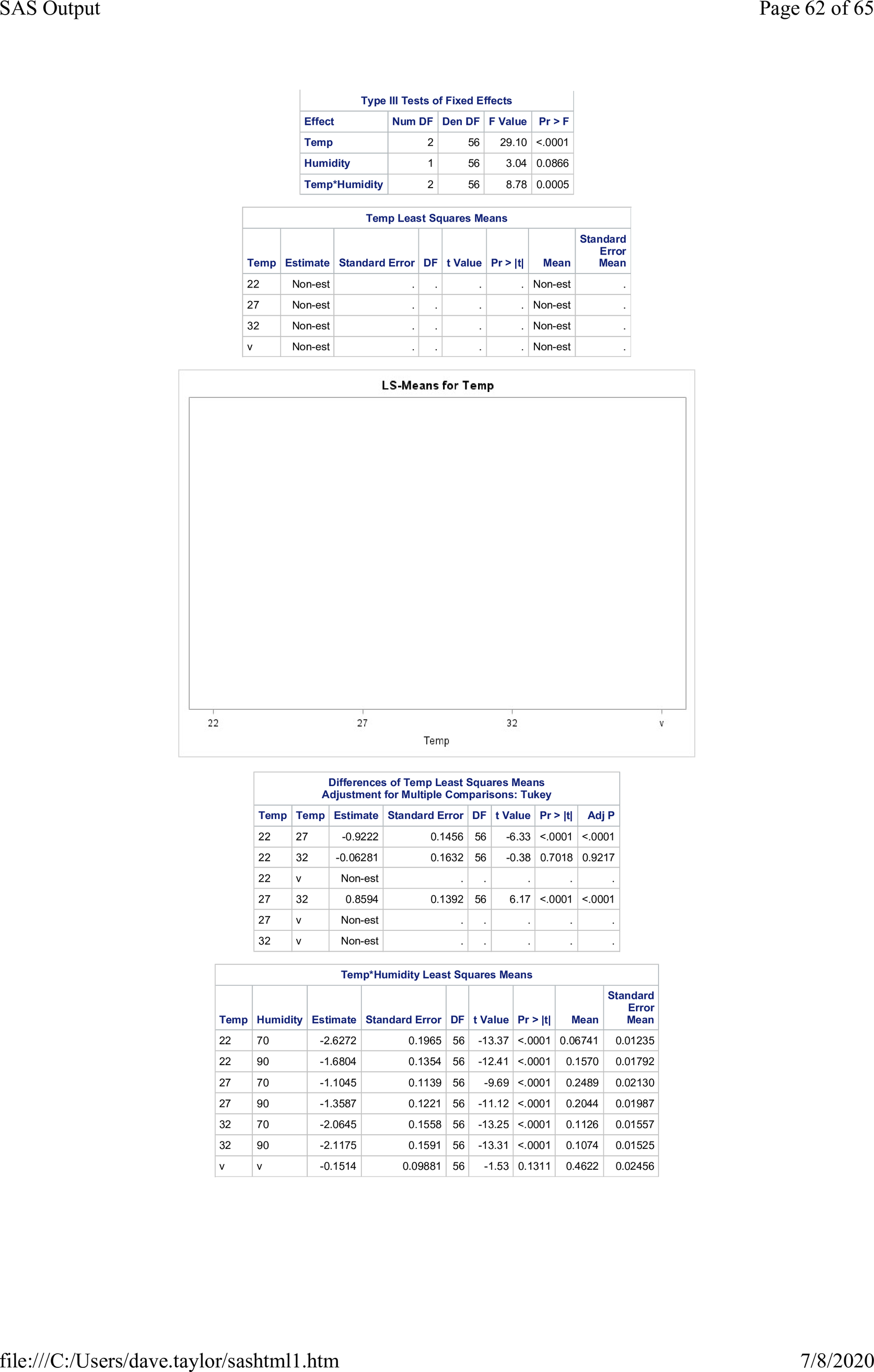

Supplement: S1 File — (ZIP) [file pone.0242794.s001.zip › PACE Corrected/S1_File.pdf.tif]

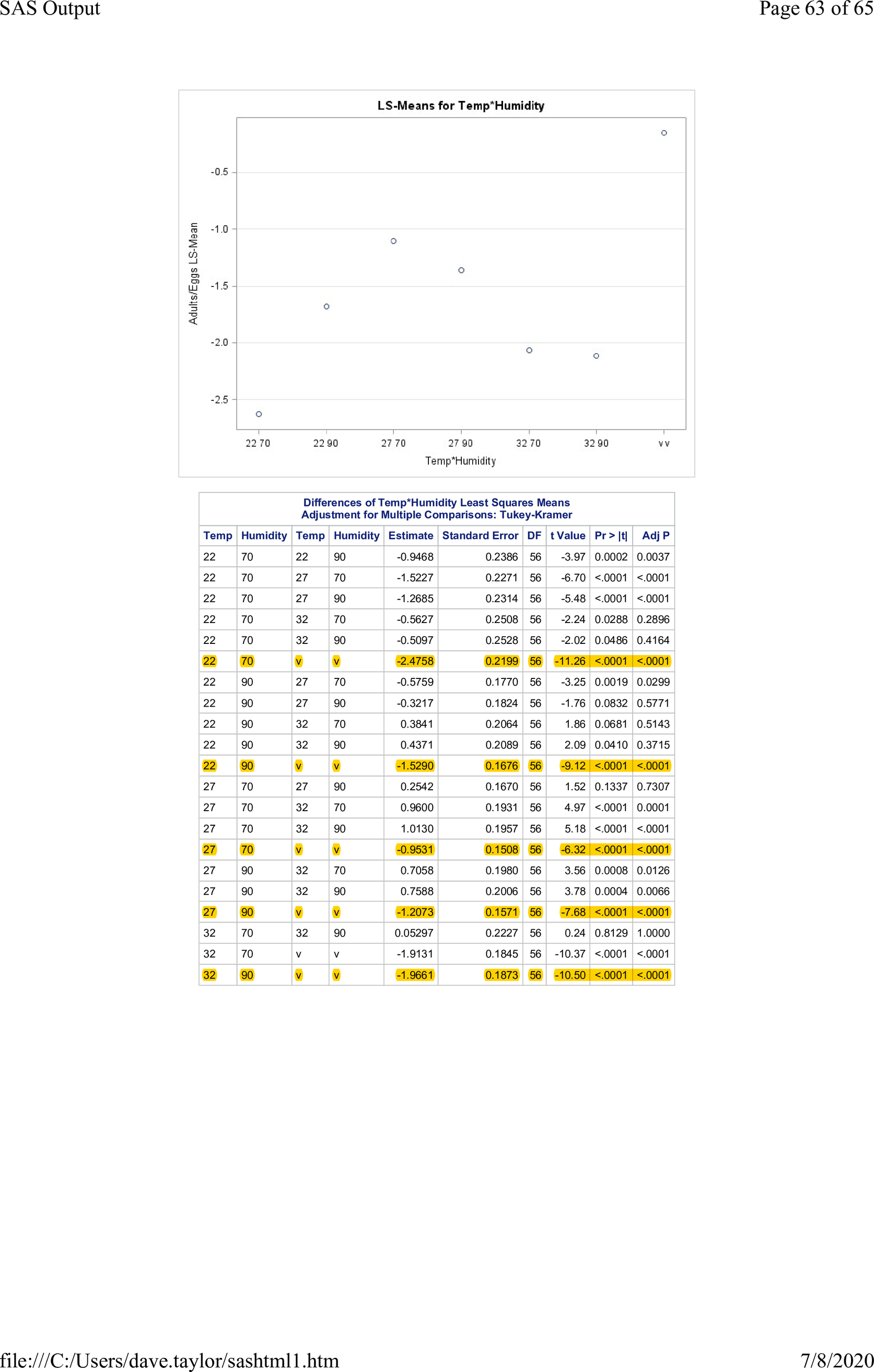

Supplement: S1 File — (ZIP) [file pone.0242794.s001.zip › PACE Corrected/S1_File.pdf.tif]

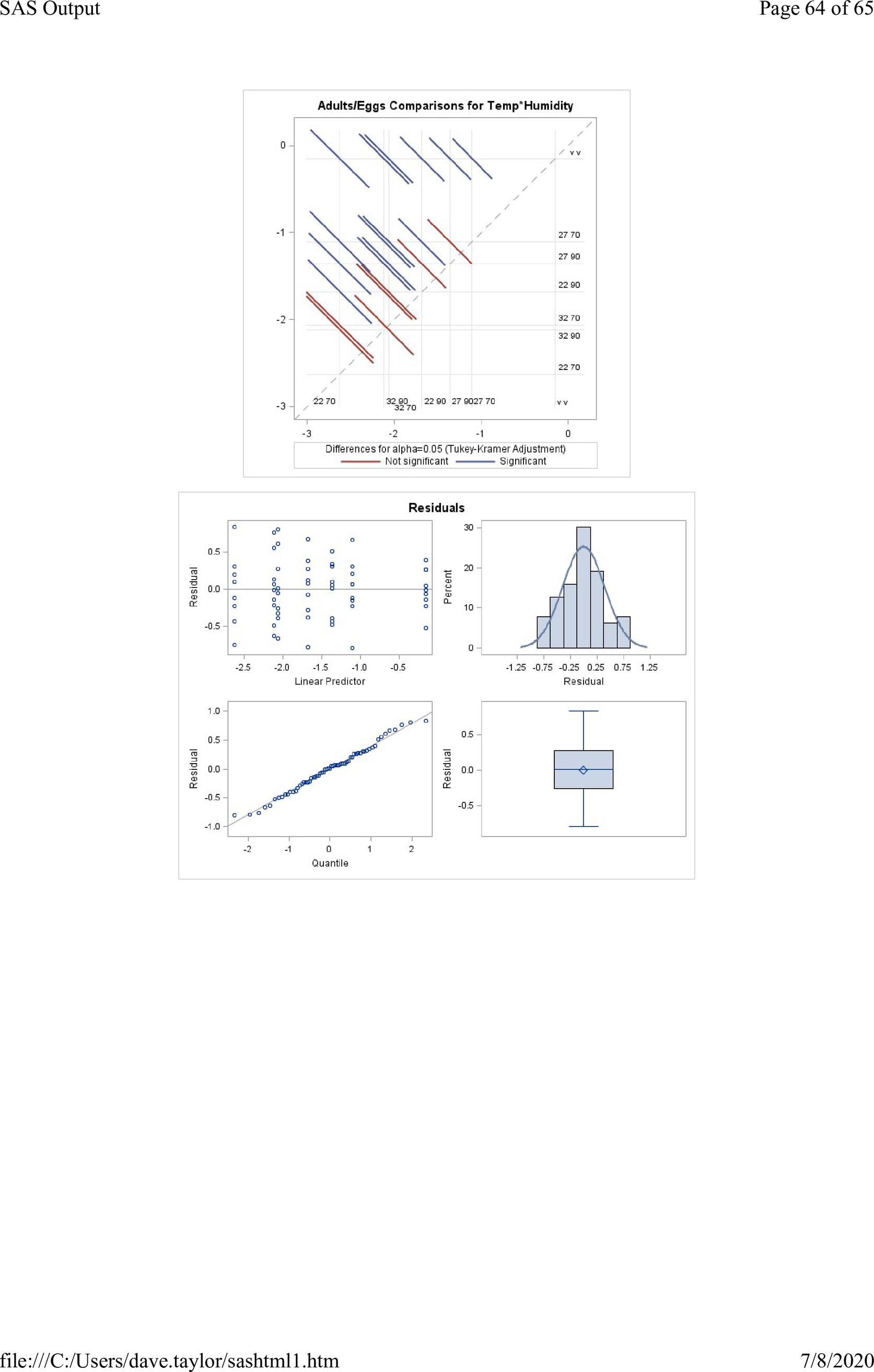

Supplement: S1 File — (ZIP) [file pone.0242794.s001.zip › PACE Corrected/S1_File.pdf.tif]

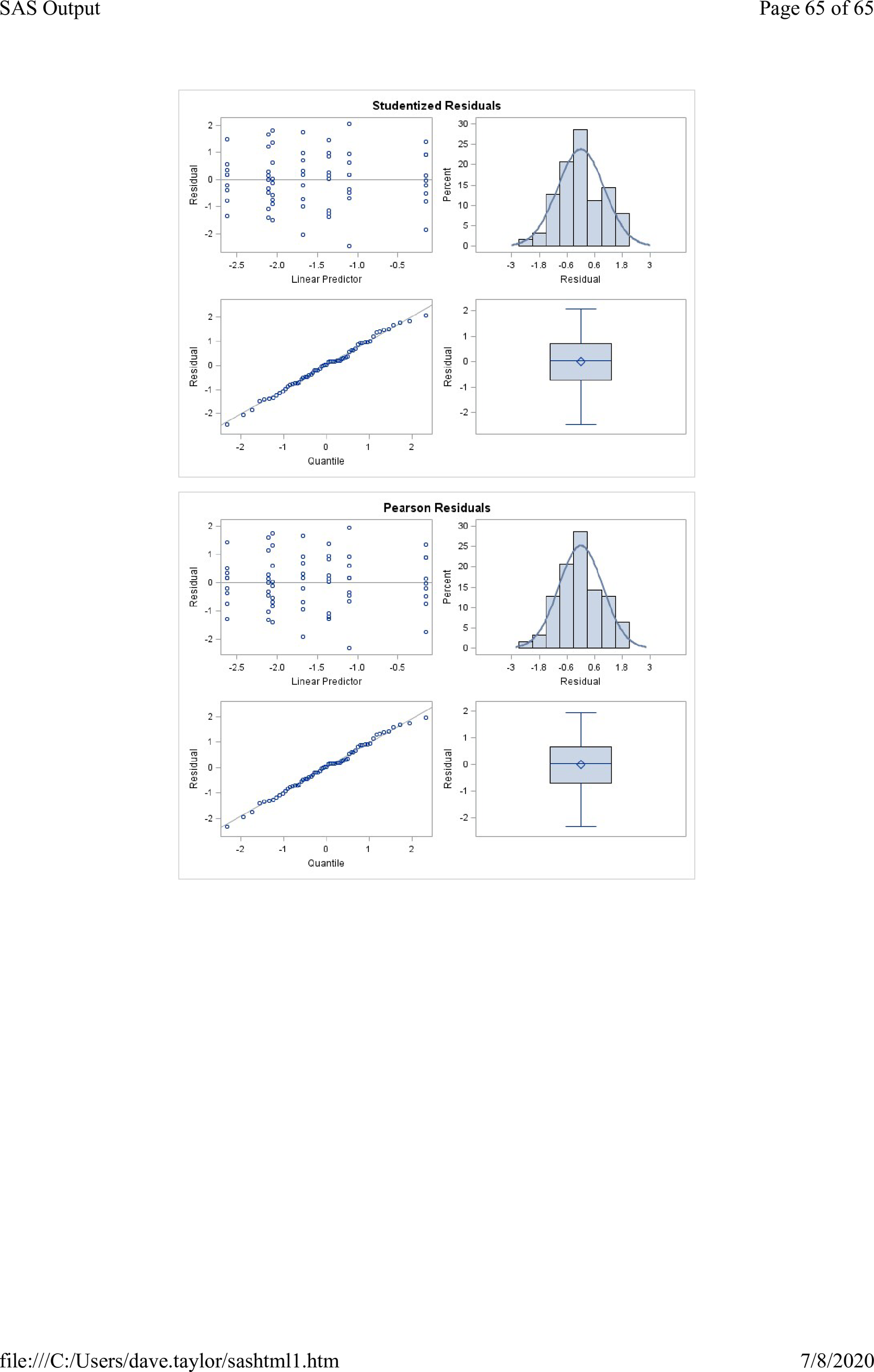

Supplement: S1 File — (ZIP) [file pone.0242794.s001.zip › PACE Corrected/S1_File.pdf.tif]
