# Supplementary material for: The combined effects of temperature and relative humidity parameters on the reproduction of Stomoxys species in a laboratory setting
Source: PLoS One. 2020 Dec 21;15(12):e0242794. doi: 10.1371/journal.pone.0242794 (PMC7751869; doi:10.1371/journal.pone.0242794)
Supplement: S2 File — (PDF) [file pone.0242794.s002.pdf]

The SAS System

Obs	Rep	Species	Temp	Humidity	Period_Egg	Eggs	Hatched	pHatched	Period_Larva	Larvae	Pupated	pPupated	Period_Pupa	Adults	pEmerged	F16
1	4	SC	v	v	29 (15-33)	150	101	67	240 (204-268)	151.020	74	49	144 (120-156)	50	33	
2	4	SS	v	v	27 (17-30)	150	138	92	240 (220-262)	150.877	86	57	132 (108-156)	67	45	
3	4	SI	v	v	23 (15-28)	150	113	75	240 (204-280)	150.000	78	52	120 (108-140)	69	46	
4	5	SC	v	v	29 (15-33)	150	131	87	240 (204-268)	149.123	85	57	144 (120-156)	64	43	
5	5	SS	v	v	27 (17-30)	150	120	80	240 (220-262)	150.000	96	64	132 (108-156)	79	53	
6	5	SI	v	v	23 (15-28)	150	127	85	240 (204-280)	150.794	95	63	120 (108-140)	79	53	
7	6	SC	v	v	29 (15-33)	150	114	76	240 (204-268)	150.000	93	62	144 (120-156)	71	47	
8	6	SS	v	v	27 (17-30)	150	134	89	240 (220-262)	150.000	108	72	132 (108-156)	84	56	
9	6	SI	v	v	23 (15-28)	150	119	79	240 (204-280)	150.847	89	59	120 (108-140)	61	41	
10	1	SC	32	90	25 (12-30)	150	110	73	240 (235-250)	148.148	40	27	144 (140-150)	14	9	
11	1	SS	32	90	28 (13-32)	150	116	77	240 (220-255)	123.810	26	21	120 (96-144)	7	5	
12	1	SI	32	90	22 (15-27)	150	140	93	264 (245-276)	217.647	37	17	120 (100-138)	9	6	
13	1	SC	27	90	27 (12-31)	150	106	71	216 (204-228)	151.852	41	27	48 (40-72)	21	14	
14	1	SS	27	90	24 (18-29)	150	59	39	216 (200-228)	147.826	34	23	72 (50-84)	20	13	
15	1	SI	27	90	24 (10-29)	150	76	51	240 (216-260)	150.000	63	42	48 (40-56)	19	13	
16	1	SC	22	90	30 (18-35)	150	89	59	312 (264-327)	158.974	62	39	52 (45-60)	16	16	
17	1	SS	22	90	26 (19-32)	150	62	41	288 (238-306)	127.586	37	29	72 (60-85)	8	20	
18	1	SI	22	90	27 (16-33)	150	95	63	288 (230-312)	132.258	41	31	72 (60-84)	18	18	
19	1	SC	32	70	30 (21-33)	150	91	61	240 (216-264)	151.351	56	37	96 (90-102)	11	7	
20	1	SS	32	70	26 (19-29)	150	103	69	240 (200-252)	151.724	44	29	96 (86-106)	16	11	
21	1	SI	32	70	25 (16-30)	150	81	54	240 (228-250)	151.429	53	35	96 (90-102)	13	9	
22	1	SC	27	70	24 (15-29)	150	75	50	240 (210-256)	150.000	51	34	72 (65-80)	34	23	
23	1	SS	27	70	24 (17-26)	150	104	69	240 (220-246)	151.429	53	35	96 (84-108)	31	21	
24	1	SI	27	70	24 (15-28)	150	86	57	216 (202-228)	150.000	42	28	72 (66-78)	15	37	
25	1	SC	22	70	26 (16-29)	150	94	63	288 (240-312)	150.000	27	18	72 (67-96)	12	8	
26	1	SS	22	70	34 (20-38)	150	90	60	288 (246-300)	151.852	41	27	72 (62-96)	18	12	
27	1	SI	22	70	31 (17-37)	150	120	80	264 (236-294)	152.941	26	17	72 (65-96)	3	2	
28	2	SC	32	90	25 (12-30)	150	128	85	240 (235-250)	148.718	58	39	144 (140-150)	24	16	
29	2	SS	32	90	28 (13-32)	150	123	82	240 (220-255)	150.000	39	26	120 (96-144)	17	11	
30	2	SI	32	90	22 (15-27)	150	133	89	264 (245-276)	151.220	62	41	120 (100-138)	18	12	
31	2	SC	27	90	27 (12-31)	150	101	67	216 (204-228)	150.000	63	42	48 (40-72)	33	22	
32	2	SS	27	90	24 (18-29)	150	76	51	216 (200-228)	148.387	46	31	72 (50-84)	39	26	
33	2	SI	27	90	24 (10-29)	150	95	63	240 (216-260)	151.064	71	47	48 (40-56)	38	25	
34	2	SC	22	90	30 (18-35)	150	105	70	312 (264-327)	148.649	55	37	52 (45-60)	25	17	
35	2	SS	22	90	26 (19-32)	150	80	53	288 (238-306)	150.000	45	30	72 (60-85)	29	19	
36	2	SI	22	90	27 (16-33)	150	103	69	288 (230-312)	151.613	47	31	72 (60-84)	26	17	
37	2	SC	32	70	30 (21-33)	150	102	68	240 (216-264)	150.000	42	28	96 (90-102)	17	11	
38	2	SS	32	70	26 (19-29)	150	79	53	240 (200-252)	150.000	36	24	96 (86-106)	29	19	
39	2	SI	32	70	25 (16-30)	150	111	74	240 (228-250)	151.852	41	27	96 (90-102)	7	5	
40	2	SC	27	70	24 (15-29)	150	86	57	240 (210-256)	151.220	62	41	72 (65-80)	43	27	
41	2	SS	27	70	24 (17-26)	150	92	61	240 (220-246)	150.000	72	48	96 (84-108)	39	26	
42	2	SI	27	70	24 (15-28)	150	68	45	216 (202-228)	148.571	52	35	72 (66-78)	39	26	
43	2	SC	22	70	26 (16-29)	150	111	74	288 (240-312)	152.174	35	23	72 (67-96)	8	5	
44	2	SS	22	70	34 (20-38)	150	101	67	288 (246-300)	148.485	49	33	72 (62-96)	13	9	
45	2	SI	22	70	31 (17-37)	150	116	77	264 (236-294)	147.826	34	23	72 (65-96)	11	7	
46	3	SC	32	90	25 (12-30)	150	120	80	240 (235-250)	150.000	48	32	144 (140-150)	27	18	
47	3	SS	32	90	28 (13-32)	150	131	87	240 (220-255)	147.619	31	21	120 (96-144)	13	9	
48	3	SI	32	90	22 (15-27)	150	128	85	264 (245-276)	150.000	54	36	120 (100-138)	16	11	
49	3	SC	27	90	27 (12-31)	150	81	54	216 (204-228)	148.889	67	45	48 (40-72)	43	29	
50	3	SS	27	90	24 (18-29)	150	82	55	216 (200-228)	151.429	53	35	72 (50-84)	32	21	
51	3	SI	27	90	24 (10-29)	150	89	59	240 (216-260)	151.613	47	31	48 (40-56)	31	21	
52	3	SC	22	90	30 (18-35)	150	102	68	312 (264-327)	151.282	59	39	52 (45-60)	31	21	
53	3	SS	22	90	26 (19-32)	150	72	48	288 (238-306)	150.000	51	34	72 (60-85)	22	15	
54	3	SI	22	90	27 (16-33)	150	85	57	288 (230-312)	150.000	54	36	72 (60-84)	37	25	

**55**	3	SC	32	70	30 (21-33)	150	112	75	240 (216-264)	152.174	35	23	96 (90-102)	26	17	
**56**	3	SS	32	70	26 (19-29)	150	84	56	240 (200-252)	150.000	36	24	96 (86-106)	21	14	
**57**	3	SI	32	70	25 (16-30)	150	88	59	240 (228-250)	150.000	60	40	96 (90-102)	12	8	
**58**	3	SC	27	70	24 (15-29)	150	90	60	240 (210-256)	151.020	74	49	72 (65-80)	56	37	
**59**	3	SS	27	70	24 (17-26)	150	84	56	240 (220-246)	150.000	81	54	96 (84-108)	46	31	
**60**	3	SI	27	70	24 (15-28)	150	72	48	216 (202-228)	150.000	60	40	72 (66-78)	33	22	
**61**	3	SC	22	70	26 (16-29)	150	97	65	288 (240-312)	151.852	41	27	72 (67-96)	6	4	
**62**	3	SS	22	70	34 (20-38)	150	97	65	288 (246-300)	150.000	30	20	72 (62-96)	9	6	
**63**	3	SI	22	70	31 (17-37)	150	99	66	264 (236-294)	151.613	47	31	72 (65-96)	11	7	

The SAS System

The GLIMMIX Procedure

Model Information	
Data Set	WORK.RAW1
Response Variable (Events)	Hatched
Response Variable (Trials)	Eggs
Response Distribution	Binomial
Link Function	Logit
Variance Function	Default
Variance Matrix	Diagonal
Estimation Technique	Maximum Likelihood
Degrees of Freedom Method	Residual

Class Level Information		
Class	Levels	Values
Species	3	SC SI SS
Temp	3	22 27 32
Humidity	2	70 90

Number of Observations Read	54
Number of Observations Used	54
Number of Events	5220
Number of Trials	8100

Dimensions	
Covariance Parameters	1
Columns in X	30
Columns in Z	0
Subjects (Blocks in V)	1
Max Obs per Subject	54

Optimization Information	
Optimization Technique	Newton-Raphson
Parameters in Optimization	14
Lower Boundaries	0
Upper Boundaries	0
Fixed Effects	Not Profiled

Iteration History					
Iteration	Restarts	Evaluations	Objective Function	Change	Max Gradient
0	0	4	218.44818376	.	23.81099
1	0	3	218.06422244	0.38396132	0.209795
2	0	3	218.06412608	0.00009636	0.000064
3	0	3	218.06412608	0.00000000	7.87E-12

Convergence criterion (GCONV=1E-8) satisfied.

Fit Statistics	
-2 Log Likelihood	436.13
AIC (smaller is better)	464.13
AICC (smaller is better)	474.90
BIC (smaller is better)	491.97
CAIC (smaller is better)	505.97
HQIC (smaller is better)	474.87
Pearson Chi-Square	150.56
Pearson Chi-Square / DF	3.76

Type III Tests of Fixed Effects				
Effect	Num DF	Den DF	F Value	Pr > F
Species	2	40	4.06	0.0249
Temp	2	40	25.10	<.0001
Humidity	1	40	6.47	0.0149
Species*Temp	4	40	1.43	0.2423
Species*Humidity	2	40	2.60	0.0869
Temp*Humidity	2	40	21.13	<.0001

Species Least Squares Means							
Species	Estimate	Standard Error	DF	t Value	Pr > |t|	Mean	Standard Error Mean
SC	0.7354	0.08190	40	8.98	<.0001	0.6760	0.01794
SI	0.7307	0.08312	40	8.79	<.0001	0.6750	0.01824
SS	0.4549	0.07864	40	5.78	<.0001	0.6118	0.01868

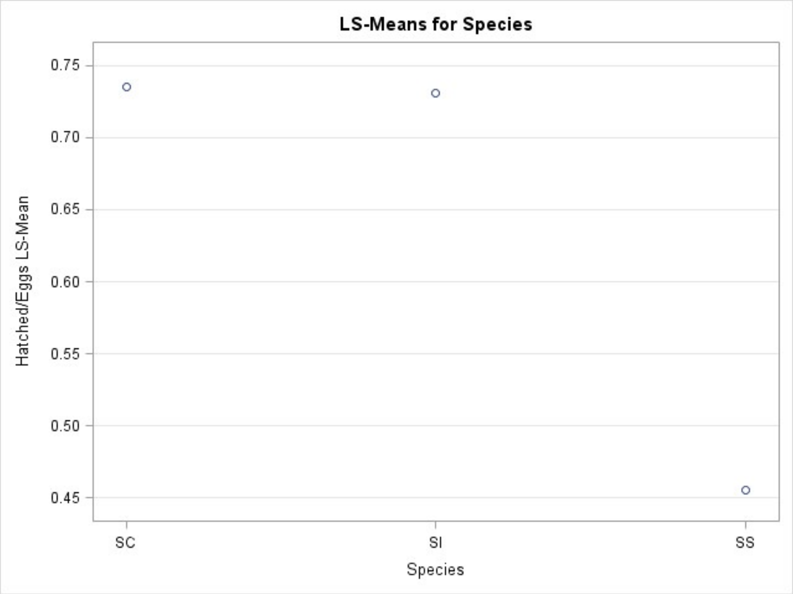

Differences of Species Least Squares Means Adjustment for Multiple Comparisons: Tukey-Kramer							
Species	Species	Estimate	Standard Error	DF	t Value	Pr > |t|	Adj P
SC	SI	0.004709	0.1161	40	0.04	0.9678	0.9991
SC	SS	0.2806	0.1132	40	2.48	0.0175	0.0452
SI	SS	0.2759	0.1140	40	2.42	0.0202	0.0517

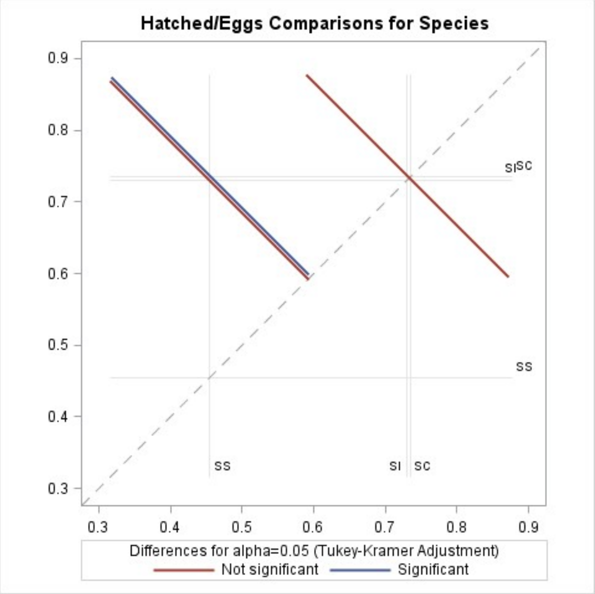

Temp Least Squares Means							
Temp	Estimate	Standard Error	DF	t Value	Pr > |t|	Mean	Standard Error Mean
22	0.5713	0.07886	40	7.24	<.0001	0.6391	0.01819
27	0.2576	0.07550	40	3.41	0.0015	0.5640	0.01857
32	1.0922	0.09056	40	12.06	<.0001	0.7488	0.01704

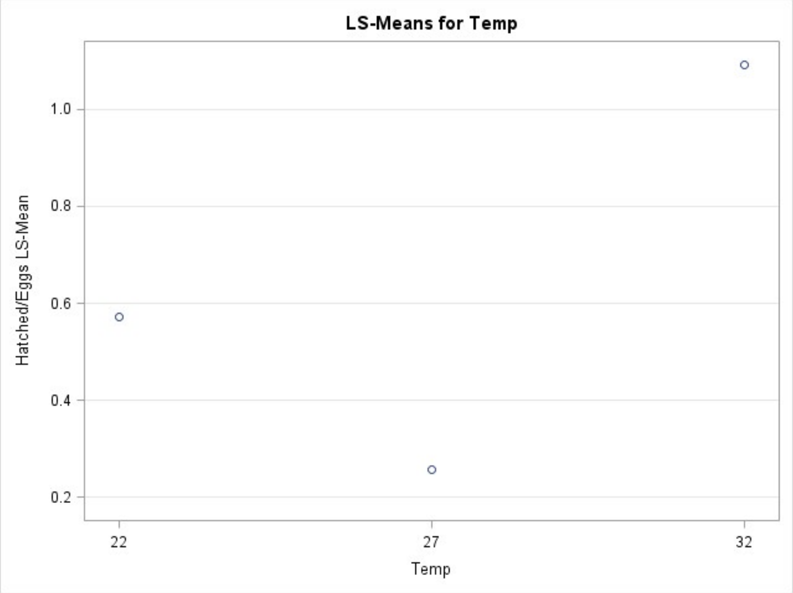

Differences of Temp Least Squares Means Adjustment for Multiple Comparisons: Tukey-Kramer							
Temp	Temp	Estimate	Standard Error	DF	t Value	Pr > |t|	Adj P
22	27	0.3137	0.1092	40	2.87	0.0065	0.0174
22	32	-0.5209	0.1201	40	-4.34	<.0001	0.0003
27	32	-0.8346	0.1179	40	-7.08	<.0001	<.0001

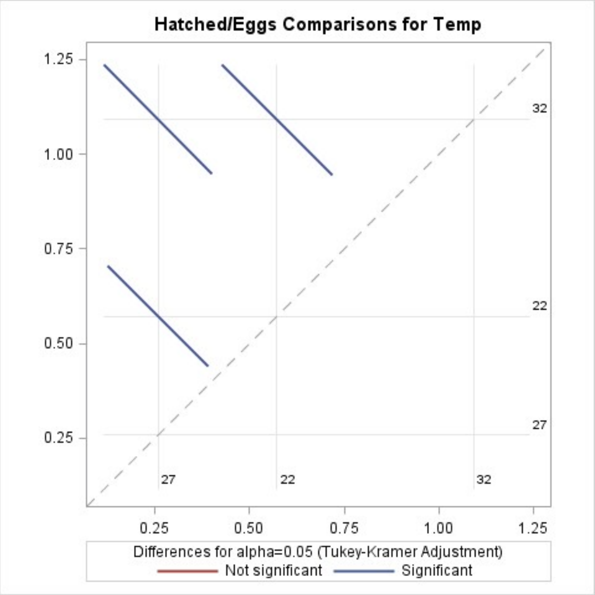

Humidity Least Squares Means							
Humidity	Estimate	Standard Error	DF	t Value	Pr > |t|	Mean	Standard Error Mean
70	0.5200	0.06354	40	8.18	<.0001	0.6272	0.01486
90	0.7607	0.07005	40	10.86	<.0001	0.6815	0.01520

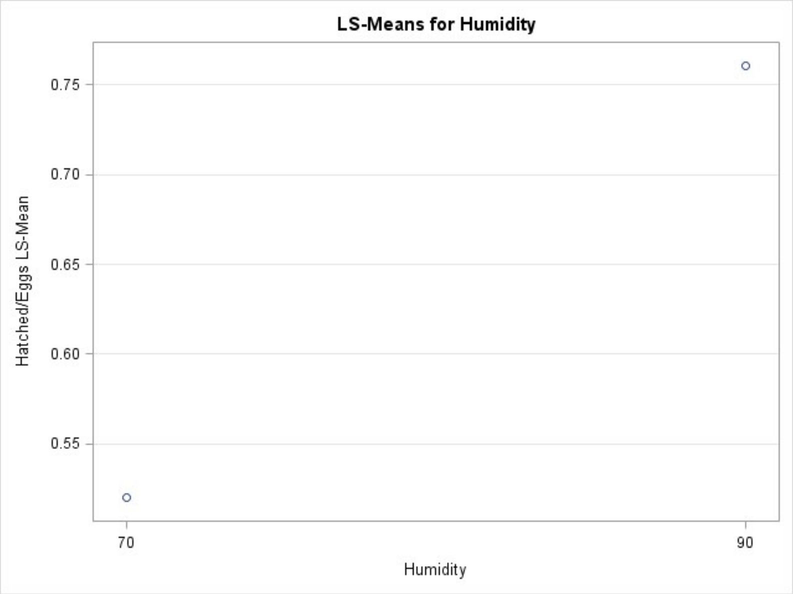

Differences of Humidity Least Squares Means Adjustment for Multiple Comparisons: Tukey-Kramer							
Humidity	Humidity	Estimate	Standard Error	DF	t Value	Pr > |t|	Adj P
70	90	-0.2406	0.09458	40	-2.54	0.0149	0.0149

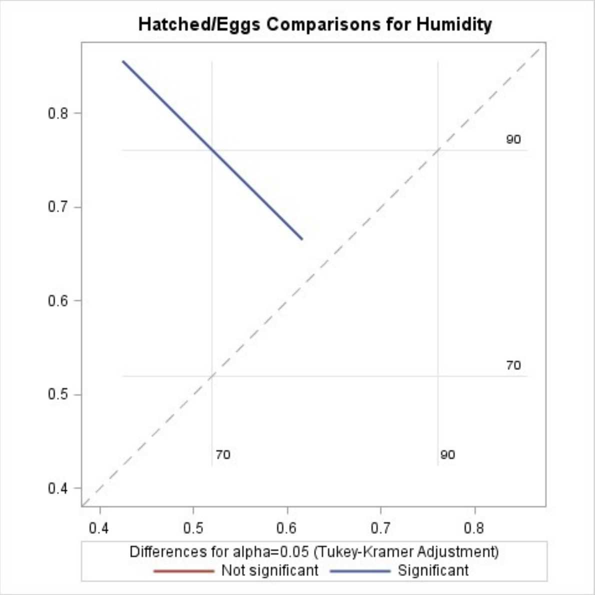

Temp*Humidity Least Squares Means								
Temp	Humidity	Estimate	Standard Error	DF	t Value	Pr > |t|	Mean	Standard Error Mean
22	70	0.7809	0.1140	40	6.85	<.0001	0.6859	0.02456
22	90	0.3617	0.1089	40	3.32	0.0019	0.5894	0.02636
27	70	0.2451	0.1066	40	2.30	0.0268	0.5610	0.02626
27	90	0.2700	0.1069	40	2.53	0.0156	0.5671	0.02625
32	70	0.5340	0.1094	40	4.88	<.0001	0.6304	0.02549
32	90	1.6503	0.1443	40	11.44	<.0001	0.8389	0.01950

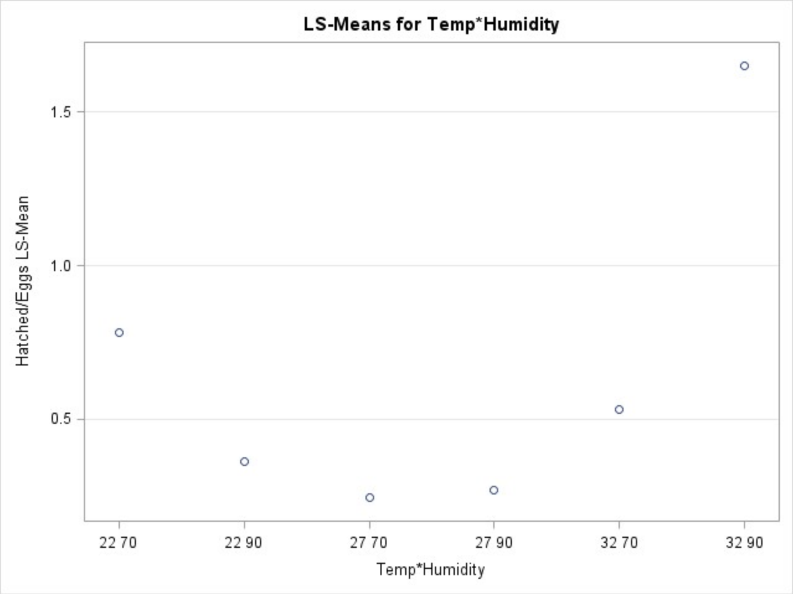

Differences of Temp*Humidity Least Squares Means Adjustment for Multiple Comparisons: Tukey-Kramer									
Temp	Humidity	Temp	Humidity	Estimate	Standard Error	DF	t Value	Pr > |t|	Adj P
22	70	22	90	0.4192	0.1576	40	2.66	0.0112	0.1064
22	70	27	70	0.5358	0.1561	40	3.43	0.0014	0.0165
22	70	27	90	0.5109	0.1563	40	3.27	0.0022	0.0252
22	70	32	70	0.2469	0.1580	40	1.56	0.1261	0.6271
22	70	32	90	-0.8694	0.1840	40	-4.73	<.0001	0.0004
22	90	27	70	0.1165	0.1524	40	0.76	0.4490	0.9719

22	90	27	90	0.09170	0.1526	40	0.60	0.5513	0.9904
22	90	32	70	-0.1724	0.1544	40	-1.12	0.2709	0.8717
22	90	32	90	-1.2887	0.1807	40	-7.13	<.0001	<.0001
27	70	27	90	-0.02483	0.1510	40	-0.16	0.8702	1.0000
27	70	32	70	-0.2889	0.1528	40	-1.89	0.0659	0.4225
27	70	32	90	-1.4052	0.1794	40	-7.83	<.0001	<.0001
27	90	32	70	-0.2641	0.1530	40	-1.73	0.0920	0.5233
27	90	32	90	-1.3804	0.1796	40	-7.69	<.0001	<.0001
32	70	32	90	-1.1163	0.1810	40	-6.17	<.0001	<.0001

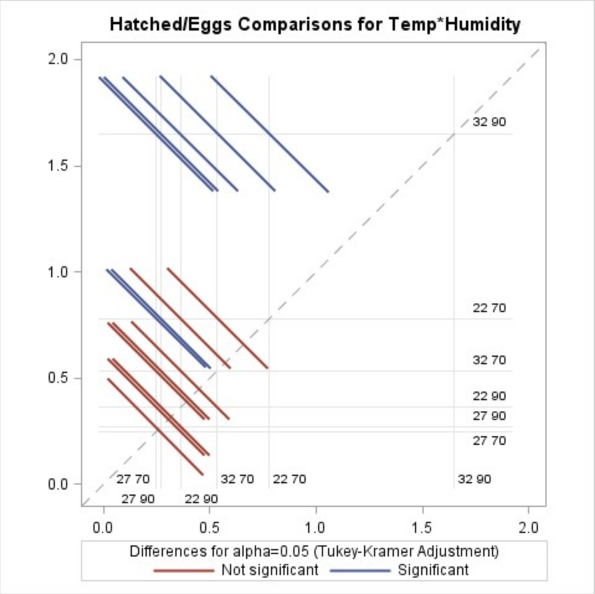

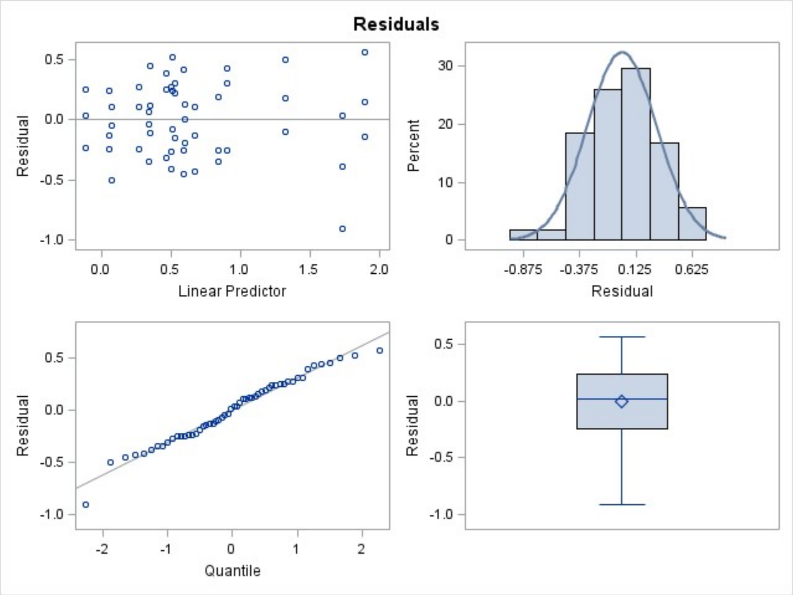

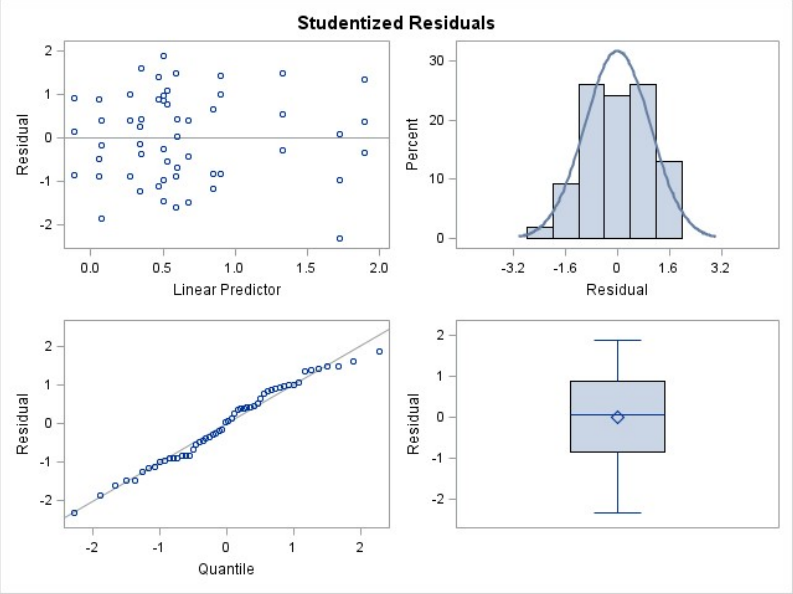
Studentized Residuals
Residual
Linear Predictor
Percent
Residual
Quantile

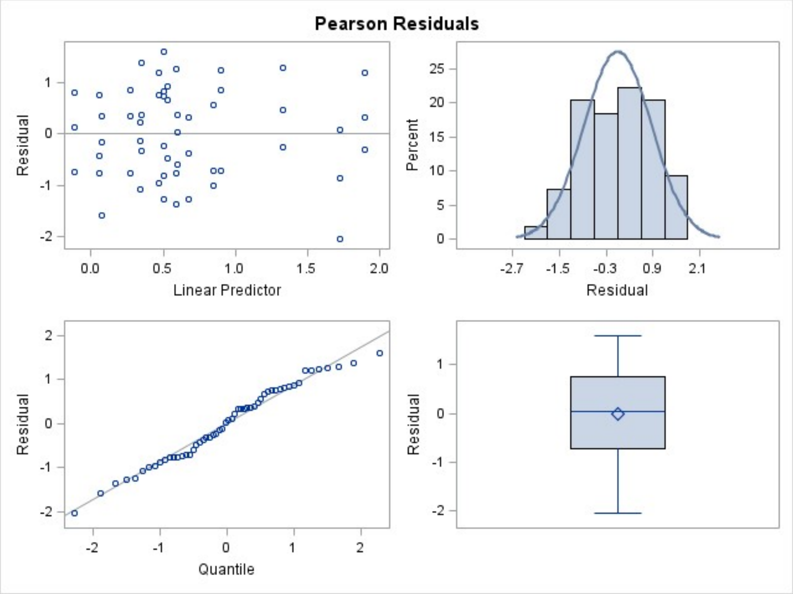
Pearson Residuals
Residual
Linear Predictor
Percent
Residual
Quantile

The SAS System

The GLIMMIX Procedure

Model Information	
Data Set	WORK.RAW1
Response Variable (Events)	Pupated
Response Variable (Trials)	Hatched
Response Distribution	Binomial
Link Function	Logit
Variance Function	Default
Variance Matrix	Diagonal
Estimation Technique	Maximum Likelihood
Degrees of Freedom Method	Residual

Class Level Information		
Class	Levels	Values
Species	3	SC SI SS
Temp	3	22 27 32
Humidity	2	70 90

Number of Observations Read	54
Number of Observations Used	54
Number of Events	2611
Number of Trials	5220

Dimensions	
Covariance Parameters	1
Columns in X	30
Columns in Z	0
Subjects (Blocks in V)	1
Max Obs per Subject	54

Optimization Information	
Optimization Technique	Newton-Raphson
Parameters in Optimization	14
Lower Boundaries	0
Upper Boundaries	0
Fixed Effects	Not Profiled

Iteration History					
Iteration	Restarts	Evaluations	Objective Function	Change	Max Gradient
0	0	4	268.98127843	.	23.97724
1	0	3	266.63918764	2.34209079	0.739898
2	0	3	266.63644906	0.00273858	0.00113
3	0	3	266.63644905	0.00000001	2.812E-9

Convergence criterion (GCONV=1E-8) satisfied.

Fit Statistics	
-2 Log Likelihood	533.27
AIC (smaller is better)	561.27
AICC (smaller is better)	572.04
BIC (smaller is better)	589.12
CAIC (smaller is better)	603.12
HQIC (smaller is better)	572.01
Pearson Chi-Square	265.55
Pearson Chi-Square / DF	6.64

Type III Tests of Fixed Effects				
Effect	Num DF	Den DF	F Value	Pr > F
Species	2	40	0.03	0.9715
Temp	2	40	19.25	<.0001
Humidity	1	40	0.02	0.8834
Species*Temp	4	40	1.21	0.3210
Species*Humidity	2	40	0.32	0.7277
Temp*Humidity	2	40	9.15	0.0005

Temp Least Squares Means							
Temp	Estimate	Standard Error	DF	t Value	Pr > |t|	Mean	Standard Error Mean
22	-0.1471	0.1289	40	-1.14	0.2605	0.4633	0.03206
27	0.7514	0.1429	40	5.26	<.0001	0.6795	0.03113
32	-0.3725	0.1205	40	-3.09	0.0036	0.4079	0.02911

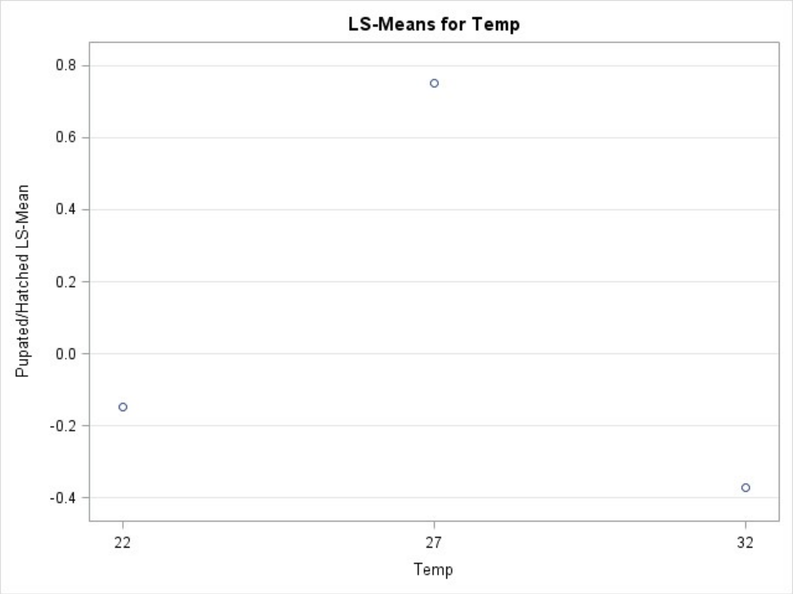

Differences of Temp Least Squares Means Adjustment for Multiple Comparisons: Tukey-Kramer							
Temp	Temp	Estimate	Standard Error	DF	t Value	Pr > |t|	Adj P
22	27	-0.8985	0.1923	40	-4.67	<.0001	<.0001
22	32	0.2254	0.1765	40	1.28	0.2089	0.4161
27	32	1.1239	0.1869	40	6.01	<.0001	<.0001

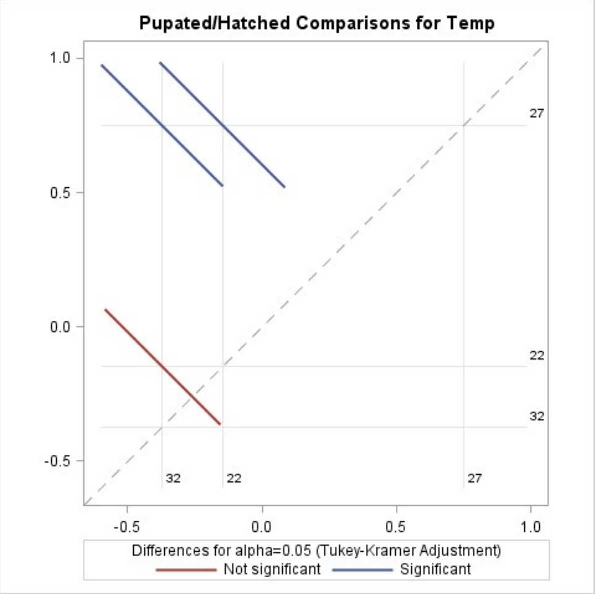

Temp*Humidity Least Squares Means								
Temp	Humidity	Estimate	Standard Error	DF	t Value	Pr > |t|	Mean	Standard Error Mean
22	70	-0.5832	0.1779	40	-3.28	0.0022	0.3582	0.04089
22	90	0.2889	0.1867	40	1.55	0.1297	0.5717	0.04572
27	70	0.9583	0.2099	40	4.57	<.0001	0.7228	0.04205
27	90	0.5445	0.1942	40	2.80	0.0078	0.6329	0.04512
32	70	-0.1098	0.1777	40	-0.62	0.5401	0.4726	0.04430
32	90	-0.6352	0.1627	40	-3.90	0.0004	0.3463	0.03683

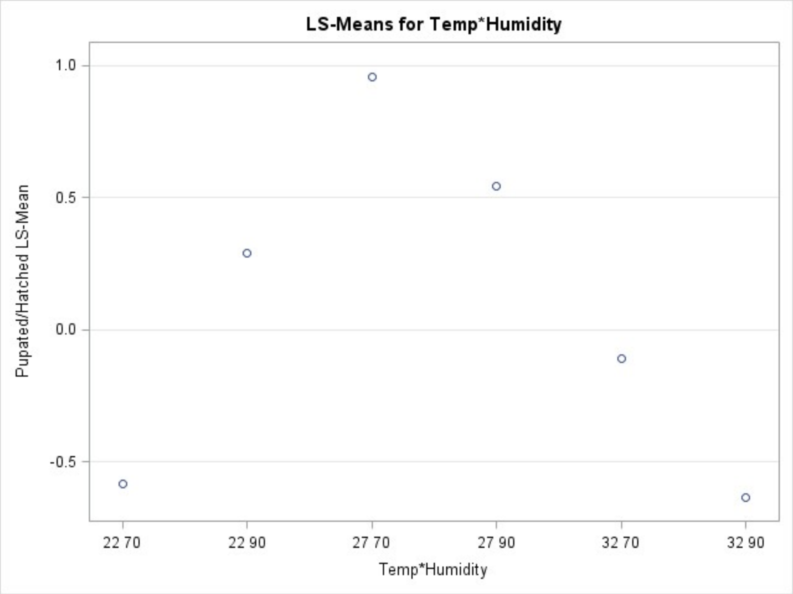

Differences of Temp*Humidity Least Squares Means Adjustment for Multiple Comparisons: Tukey-Kramer									
Temp	Humidity	Temp	Humidity	Estimate	Standard Error	DF	t Value	Pr > |t|	Adj P
22	70	22	90	-0.8721	0.2579	40	-3.38	0.0016	0.0188
22	70	27	70	-1.5414	0.2751	40	-5.60	<.0001	<.0001
22	70	27	90	-1.1277	0.2633	40	-4.28	0.0001	0.0015
22	70	32	70	-0.4733	0.2515	40	-1.88	0.0671	0.4276
22	70	32	90	0.05205	0.2411	40	0.22	0.8301	0.9999
22	90	27	70	-0.6694	0.2807	40	-2.38	0.0219	0.1861

22	90	27	90	-0.2556	0.2691	40	-0.95	0.3479	0.9307
22	90	32	70	0.3987	0.2580	40	1.55	0.1301	0.6378
22	90	32	90	0.9241	0.2473	40	3.74	0.0006	0.0072
27	70	27	90	0.4138	0.2860	40	1.45	0.1557	0.6988
27	70	32	70	1.0681	0.2750	40	3.88	0.0004	0.0047
27	70	32	90	1.5935	0.2654	40	6.01	<.0001	<.0001
27	90	32	70	0.6543	0.2634	40	2.48	0.0173	0.1532
27	90	32	90	1.1797	0.2532	40	4.66	<.0001	0.0005
32	70	32	90	0.5254	0.2408	40	2.18	0.0351	0.2688

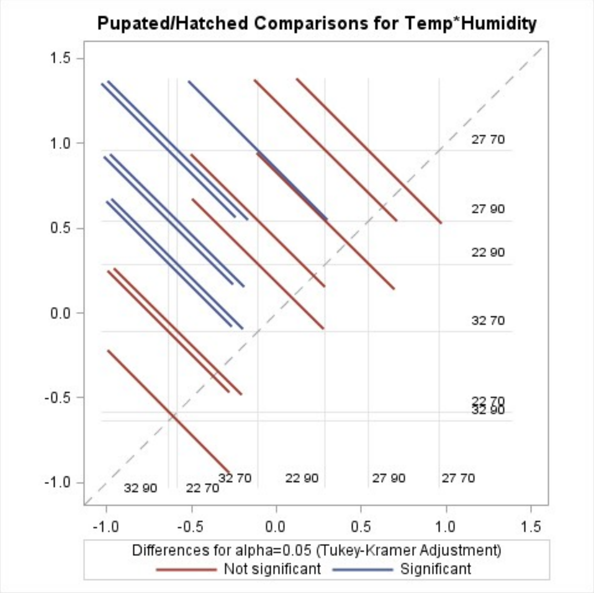

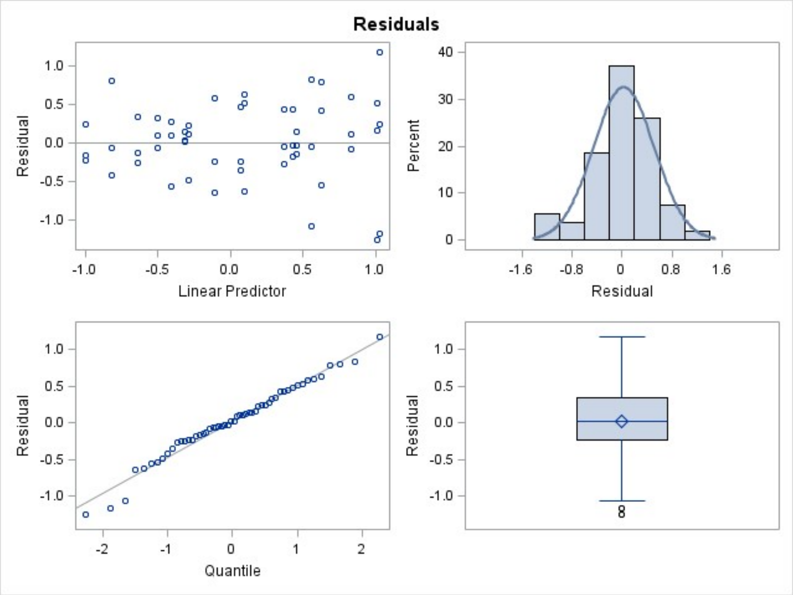

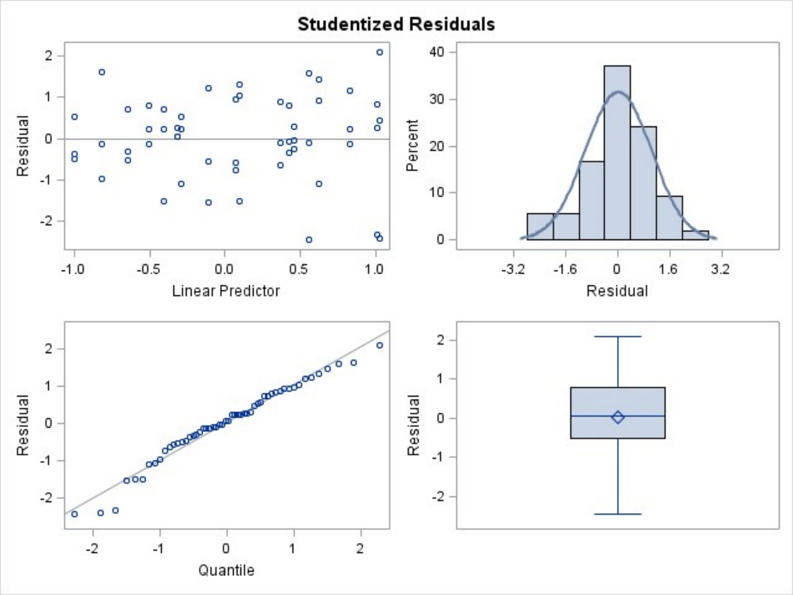
Studentized Residuals
Residual
Linear Predictor
Percent
Residual
Quantile

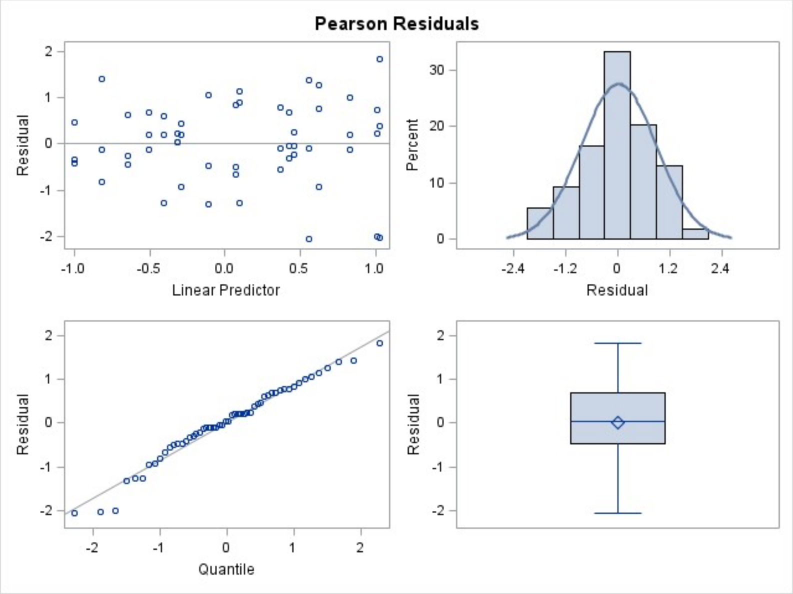
Pearson Residuals
Residual
Linear Predictor
Percent
Residual
Quantile

The SAS System

The GLIMMIX Procedure

Model Information	
Data Set	WORK.RAW1
Response Variable (Events)	Pupated
Response Variable (Trials)	Hatched
Response Distribution	Binomial
Link Function	Logit
Variance Function	Default
Variance Matrix	Diagonal
Estimation Technique	Maximum Likelihood
Degrees of Freedom Method	Residual

Class Level Information		
Class	Levels	Values
Species	3	SC SI SS
Temp	3	22 27 32
Humidity	2	70 90

Number of Observations Read	54
Number of Observations Used	54
Number of Events	2611
Number of Trials	5220

Dimensions	
Covariance Parameters	1
Columns in X	12
Columns in Z	0
Subjects (Blocks in V)	1
Max Obs per Subject	54

Optimization Information	
Optimization Technique	Newton-Raphson
Parameters in Optimization	6
Lower Boundaries	0
Upper Boundaries	0
Fixed Effects	Not Profiled

Iteration History					
Iteration	Restarts	Evaluations	Objective Function	Change	Max Gradient
0	0	4	288.12587042	.	22.01013
1	0	3	286.25998033	1.86589009	0.605048
2	0	3	286.25882174	0.00115858	0.000511
3	0	3	286.25882174	0.00000000	3.81E-10

Convergence criterion (GCONV=1E-8) satisfied.

Fit Statistics	
-2 Log Likelihood	572.52
AIC (smaller is better)	584.52
AICC (smaller is better)	586.30
BIC (smaller is better)	596.45
CAIC (smaller is better)	602.45
HQIC (smaller is better)	589.12
Pearson Chi-Square	301.18
Pearson Chi-Square / DF	6.27

Type III Tests of Fixed Effects

Effect	Num DF	Den DF	F Value	Pr > F
Temp	2	48	20.45	<.0001
Humidity	1	48	0.02	0.8997
Temp*Humidity	2	48	9.59	0.0003

Temp Least Squares Means

Temp	Estimate	Standard Error	DF	t Value	Pr > |t|	Mean	Standard Error Mean
22	-0.1564	0.1243	48	-1.26	0.2144	0.4610	0.03089
27	0.7534	0.1385	48	5.44	<.0001	0.6799	0.03014
32	-0.3627	0.1162	48	-3.12	0.0030	0.4103	0.02811

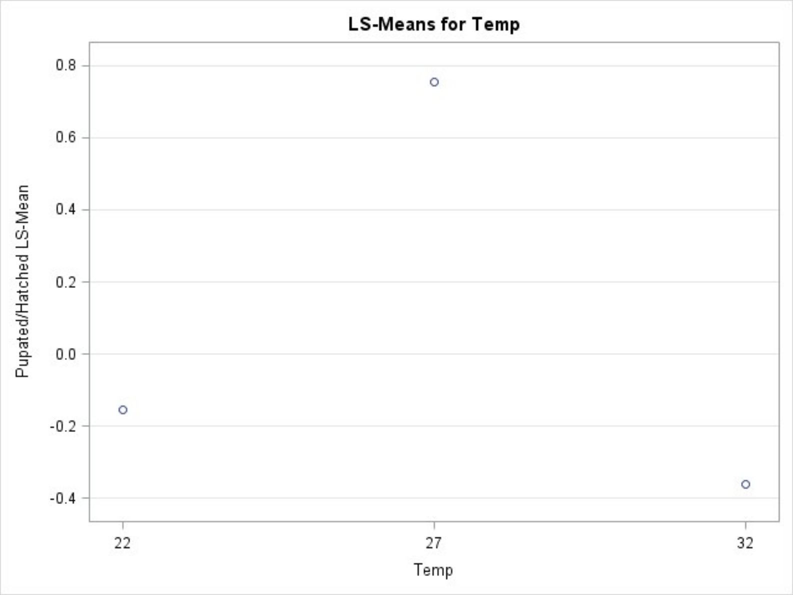

Differences of Temp Least Squares Means
Adjustment for Multiple Comparisons: Tukey-Kramer

Temp	Temp	Estimate	Standard Error	DF	t Value	Pr > |t|	Adj P
22	27	-0.9098	0.1861	48	-4.89	<.0001	<.0001
22	32	0.2063	0.1702	48	1.21	0.2312	0.4517
27	32	1.1161	0.1808	48	6.17	<.0001	<.0001

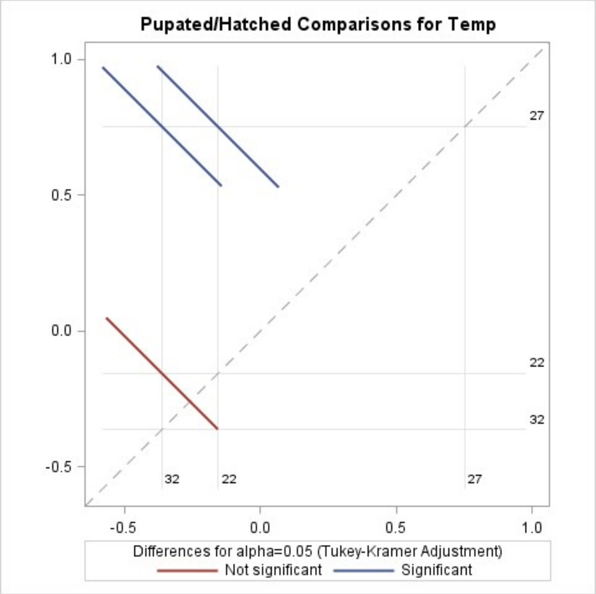

Temp*Humidity Least Squares Means								
Temp	Humidity	Estimate	Standard Error	DF	t Value	Pr > |t|	Mean	Standard Error Mean
22	70	-0.5895	0.1719	48	-3.43	0.0013	0.3568	0.03945
22	90	0.2767	0.1796	48	1.54	0.1300	0.5687	0.04405
27	70	0.9573	0.2033	48	4.71	<.0001	0.7226	0.04076
27	90	0.5494	0.1880	48	2.92	0.0053	0.6340	0.04363
32	70	-0.1059	0.1720	48	-0.62	0.5411	0.4736	0.04287
32	90	-0.6196	0.1563	48	-3.96	0.0002	0.3499	0.03555

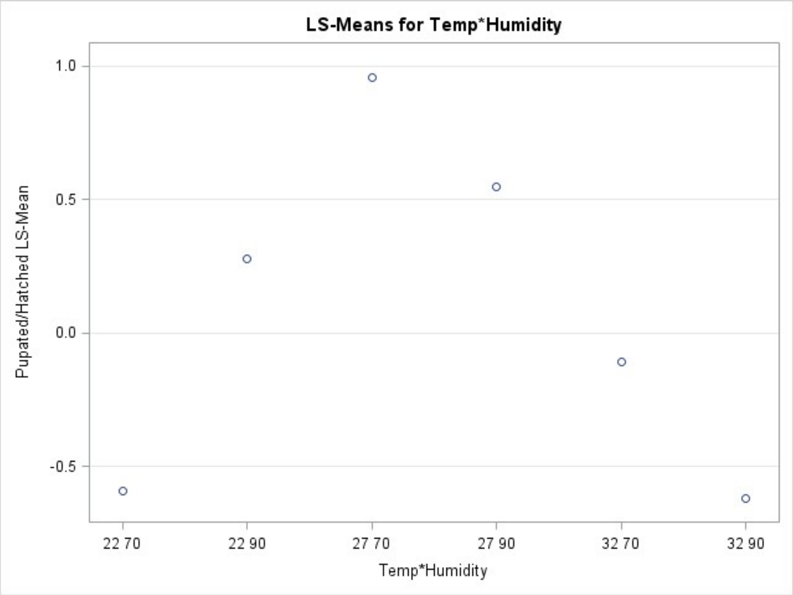

Differences of Temp*Humidity Least Squares Means Adjustment for Multiple Comparisons: Tukey-Kramer									
Temp	Humidity	Temp	Humidity	Estimate	Standard Error	DF	t Value	Pr > |t|	Adj P
22	70	22	90	-0.8661	0.2486	48	-3.48	0.0011	0.0129
22	70	27	70	-1.5468	0.2663	48	-5.81	<.0001	<.0001
22	70	27	90	-1.1388	0.2548	48	-4.47	<.0001	0.0006
22	70	32	70	-0.4836	0.2432	48	-1.99	0.0524	0.3637
22	70	32	90	0.03015	0.2324	48	0.13	0.8973	1.0000
22	90	27	70	-0.6807	0.2713	48	-2.51	0.0155	0.1419
22	90	27	90	-0.2727	0.2600	48	-1.05	0.2995	0.8987
22	90	32	70	0.3825	0.2487	48	1.54	0.1306	0.6419
22	90	32	90	0.8963	0.2381	48	3.76	0.0005	0.0057
27	70	27	90	0.4080	0.2769	48	1.47	0.1472	0.6825
27	70	32	70	1.0632	0.2663	48	3.99	0.0002	0.0029
27	70	32	90	1.5770	0.2565	48	6.15	<.0001	<.0001
27	90	32	70	0.6552	0.2548	48	2.57	0.0133	0.1244
27	90	32	90	1.1690	0.2445	48	4.78	<.0001	0.0002
32	70	32	90	0.5138	0.2324	48	2.21	0.0319	0.2519

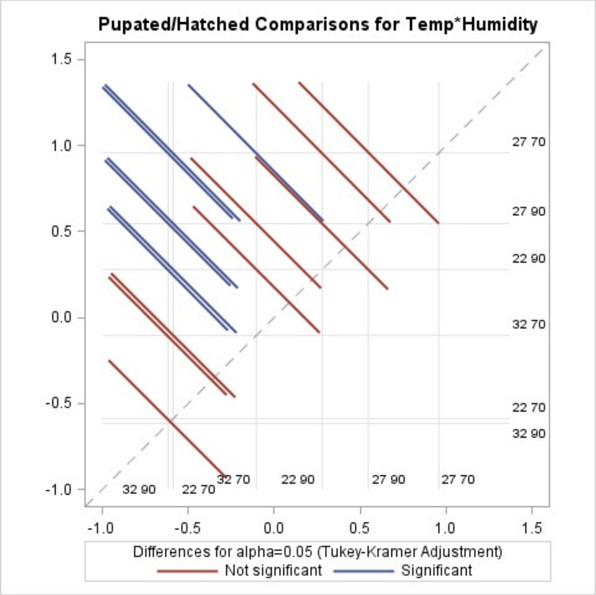
Pupated/Hatched Comparisons for Temp*Humidity
1.5
1.0
0.5
0.0
-0.5
-1.0
27 70
27 90
22 90
32 70
22 70
32 90
32 90
22 70
32 70
22 90
27 90
27 70
-1.0
-0.5
0.0
0.5
1.0
1.5
Differences for alpha=0.05 (Tukey-Kramer Adjustment)
Not significant
Significant

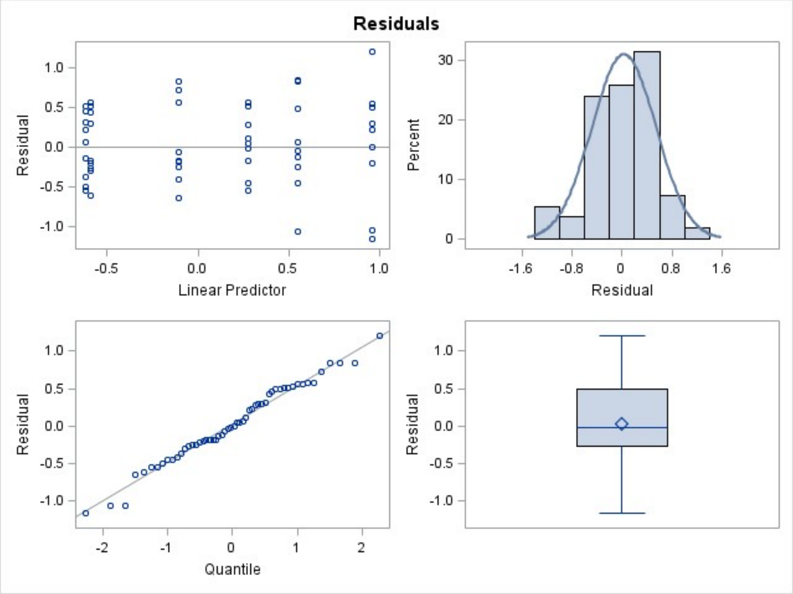
Residuals
Residual
Linear Predictor
Percent
Residual
Residual
Quantile
Residual

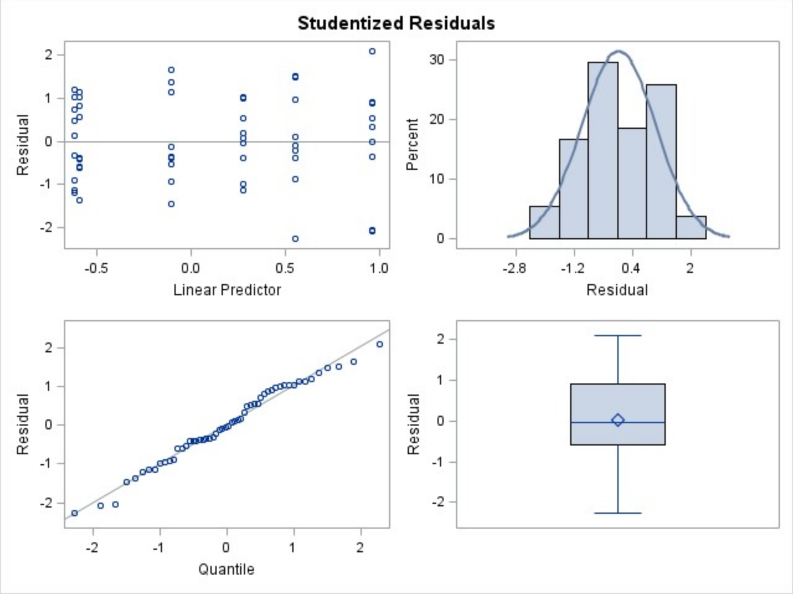
Studentized Residuals
Residual
Linear Predictor
Percent
Residual
Quantile

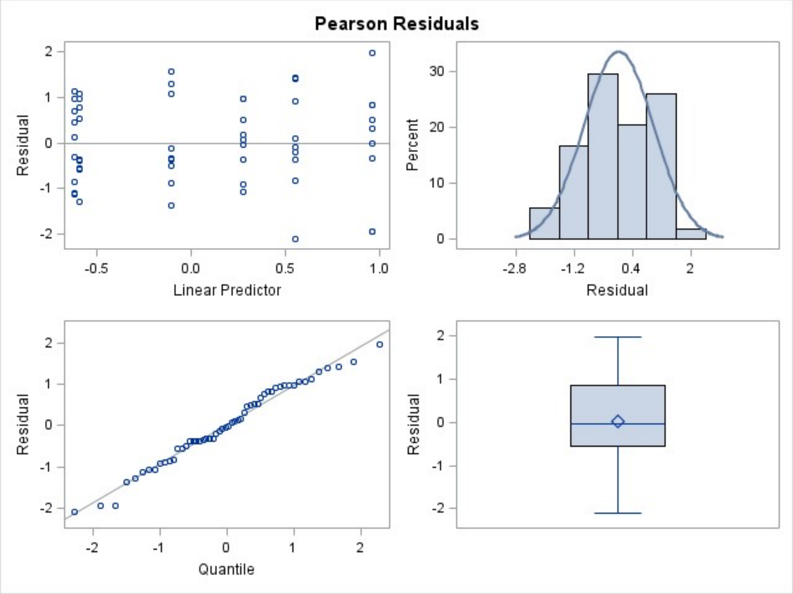
Pearson Residuals
Residual
Linear Predictor
Percent
Residual
Quantile

The SAS System

The GLIMMIX Procedure

Model Information	
Data Set	WORK.RAW1
Response Variable (Events)	Adults
Response Variable (Trials)	Pupated
Response Distribution	Binomial
Link Function	Logit
Variance Function	Default
Variance Matrix	Diagonal
Estimation Technique	Maximum Likelihood
Degrees of Freedom Method	Residual

Class Level Information		
Class	Levels	Values
Species	3	SC SI SS
Temp	3	22 27 32
Humidity	2	70 90

Number of Observations Read	54
Number of Observations Used	54
Number of Events	1212
Number of Trials	2611

Dimensions	
Covariance Parameters	1
Columns in X	30
Columns in Z	0
Subjects (Blocks in V)	1
Max Obs per Subject	54

Optimization Information	
Optimization Technique	Newton-Raphson
Parameters in Optimization	14
Lower Boundaries	0
Upper Boundaries	0
Fixed Effects	Not Profiled

Iteration History					
Iteration	Restarts	Evaluations	Objective Function	Change	Max Gradient
0	0	4	196.1548467	.	1.579923
1	0	3	195.82397777	0.33086893	0.027079
2	0	3	195.82395708	0.00002069	6.543E-6

Convergence criterion (ABSGCONV=0.00001) satisfied.

Fit Statistics	
-2 Log Likelihood	391.65
AIC (smaller is better)	419.65
AICC (smaller is better)	430.42
BIC (smaller is better)	447.49
CAIC (smaller is better)	461.49
HQIC (smaller is better)	430.39
Pearson Chi-Square	161.85
Pearson Chi-Square / DF	4.05

Type III Tests of Fixed Effects				

Effect	Num DF	Den DF	F Value	Pr > F
Species	2	40	2.49	0.0955
Temp	2	40	13.67	<.0001
Humidity	1	40	1.85	0.1810
Species*Temp	4	40	1.79	0.1500
Species*Humidity	2	40	0.47	0.6256
Temp*Humidity	2	40	3.50	0.0398

Temp Least Squares Means

Temp	Estimate	Standard Error	DF	t Value	Pr > |t|	Mean	Standard Error Mean
22	-0.5358	0.1567	40	-3.42	0.0015	0.3692	0.03649
27	0.3661	0.1288	40	2.84	0.0070	0.5905	0.03115
32	-0.4946	0.1519	40	-3.26	0.0023	0.3788	0.03574

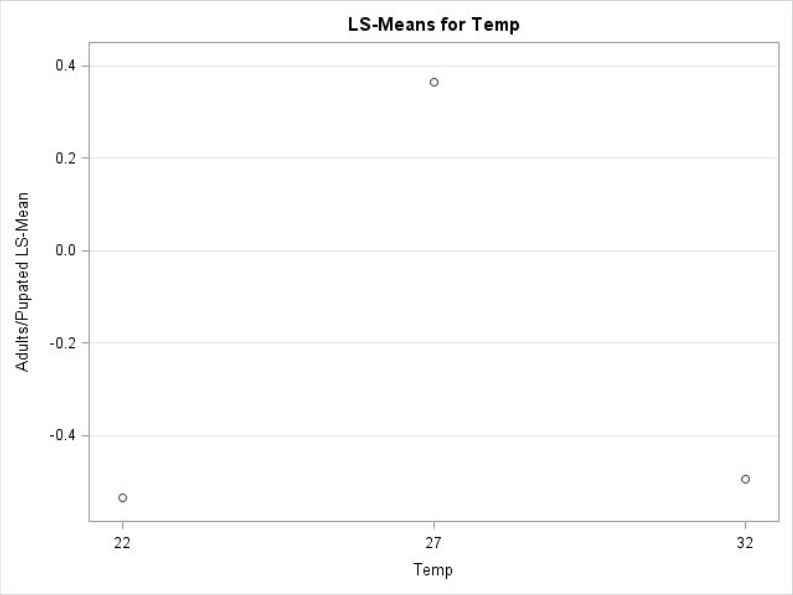

Differences of Temp Least Squares Means
Adjustment for Multiple Comparisons: Tukey-Kramer

Temp	Temp	Estimate	Standard Error	DF	t Value	Pr > |t|	Adj P
22	27	-0.9018	0.2026	40	-4.45	<.0001	0.0002
22	32	-0.04120	0.2181	40	-0.19	0.8511	0.9805
27	32	0.8606	0.1989	40	4.33	<.0001	0.0003

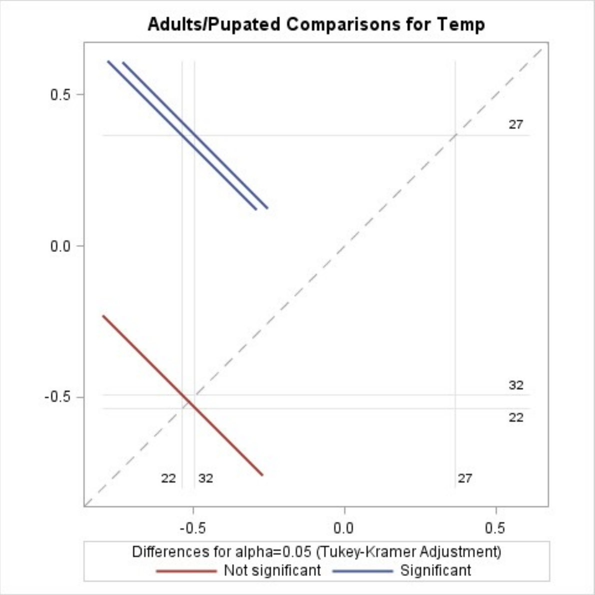

Temp*Humidity Least Squares Means								
**Temp**	**Humidity**	**Estimate**	**Standard Error**	**DF**	**t Value**	**Pr > |t|**	**Mean**	**Standard Error Mean**
22	70	-0.9713	0.2486	40	-3.91	0.0004	0.2746	0.04952
22	90	-0.1002	0.1917	40	-0.52	0.6039	0.4750	0.04780
27	70	0.4465	0.1789	40	2.50	0.0168	0.6098	0.04257
27	90	0.2856	0.1861	40	1.54	0.1326	0.5709	0.04558
32	70	-0.4848	0.2139	40	-2.27	0.0289	0.3811	0.05044
32	90	-0.5043	0.2152	40	-2.34	0.0242	0.3765	0.05052

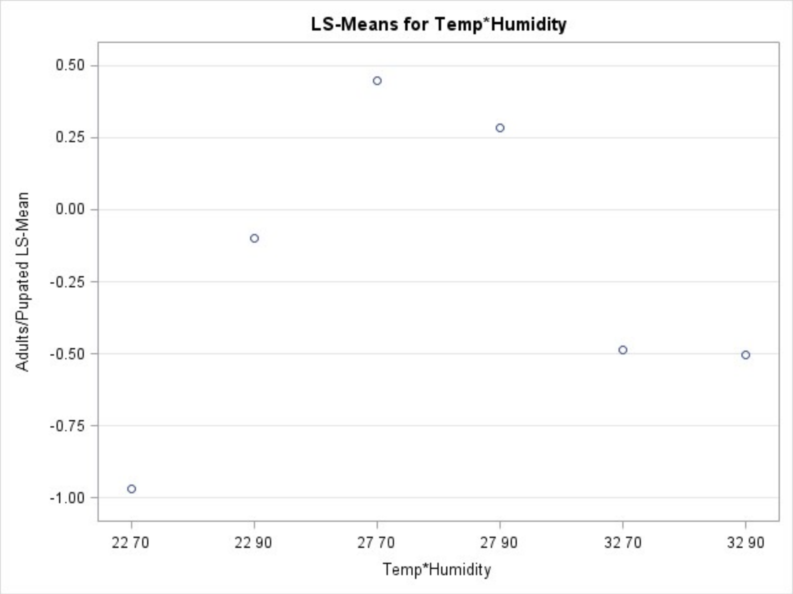

Differences of Temp*Humidity Least Squares Means Adjustment for Multiple Comparisons: Tukey-Kramer									
**Temp**	**Humidity**	**Temp**	**Humidity**	**Estimate**	**Standard Error**	**DF**	**t Value**	**Pr > |t|**	**Adj P**
22	70	22	90	-0.8711	0.3144	40	-2.77	0.0084	0.0835
22	70	27	70	-1.4178	0.3061	40	-4.63	<.0001	0.0005
22	70	27	90	-1.2569	0.3102	40	-4.05	0.0002	0.0029
22	70	32	70	-0.4865	0.3280	40	-1.48	0.1459	0.6767
22	70	32	90	-0.4670	0.3284	40	-1.42	0.1627	0.7136
22	90	27	70	-0.5467	0.2621	40	-2.09	0.0434	0.3150

22	90	27	90	-0.3859	0.2670	40	-1.45	0.1562	0.6997
22	90	32	70	0.3846	0.2873	40	1.34	0.1882	0.7620
22	90	32	90	0.4041	0.2879	40	1.40	0.1682	0.7248
27	70	27	90	0.1609	0.2586	40	0.62	0.5374	0.9887
27	70	32	70	0.9313	0.2788	40	3.34	0.0018	0.0209
27	70	32	90	0.9508	0.2796	40	3.40	0.0015	0.0179
27	90	32	70	0.7705	0.2834	40	2.72	0.0097	0.0937
27	90	32	90	0.7899	0.2841	40	2.78	0.0082	0.0816
32	70	32	90	0.01944	0.3030	40	0.06	0.9492	1.0000

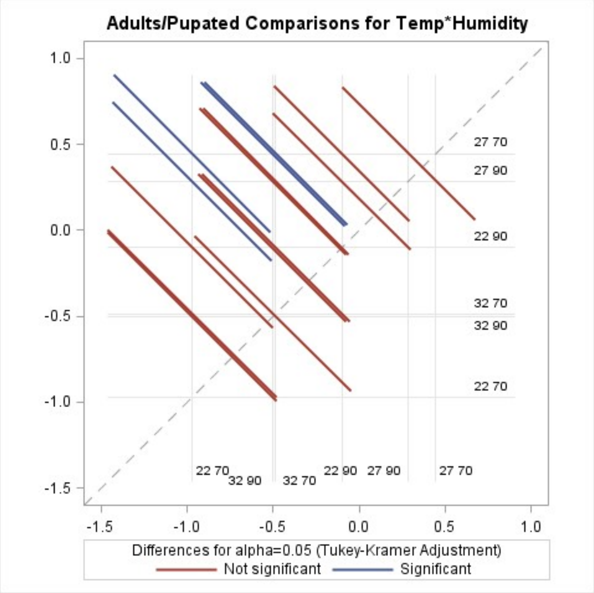

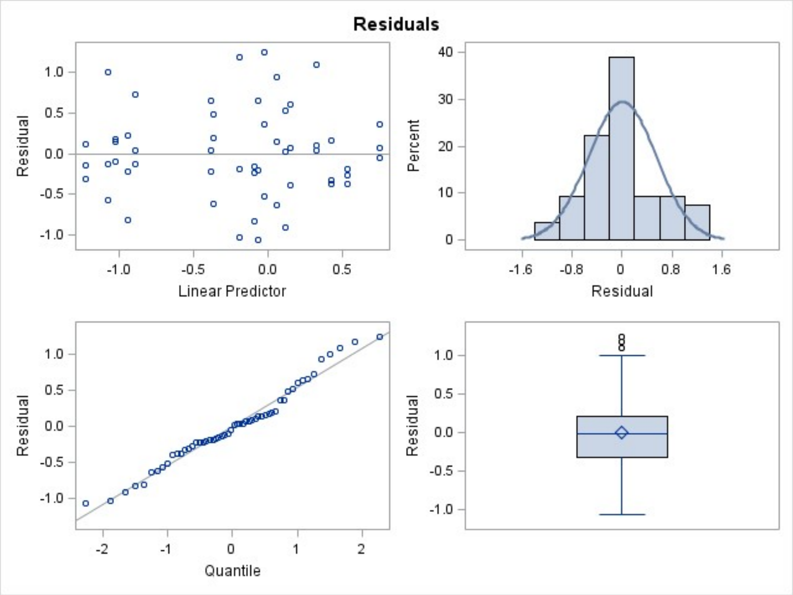

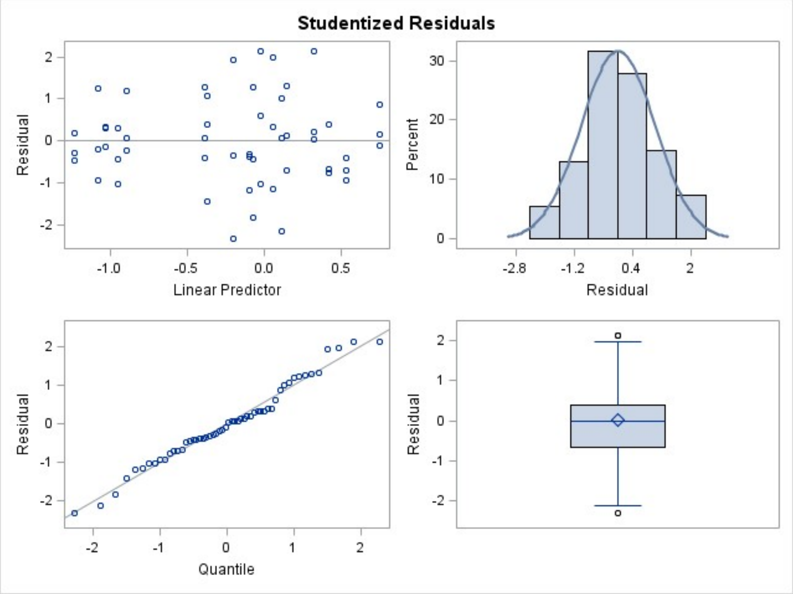
Studentized Residuals
Residual
Linear Predictor
Percent
Residual
Quantile

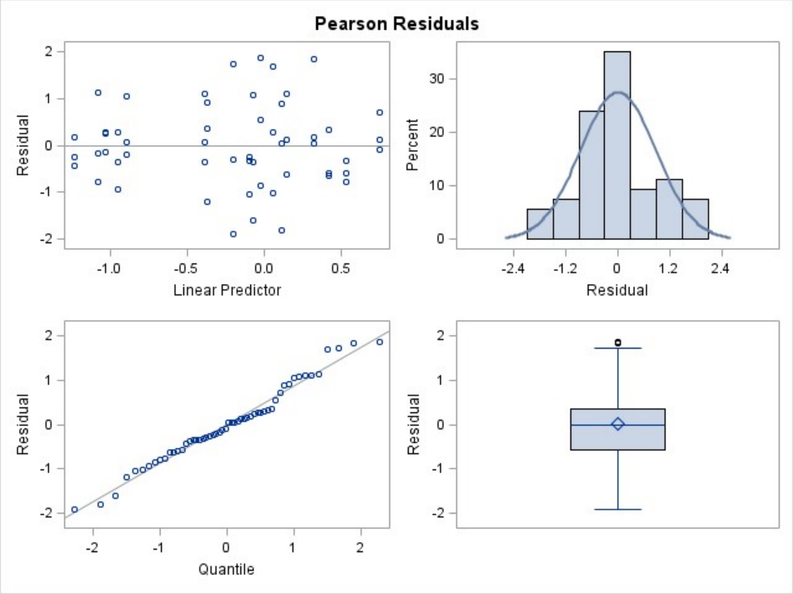
Pearson Residuals
Residual
Linear Predictor
Percent
Residual
Quantile

The SAS System

The GLIMMIX Procedure

Model Information	
Data Set	WORK.RAW1
Response Variable (Events)	Adults
Response Variable (Trials)	Pupated
Response Distribution	Binomial
Link Function	Logit
Variance Function	Default
Variance Matrix	Diagonal
Estimation Technique	Maximum Likelihood
Degrees of Freedom Method	Residual

Class Level Information		
Class	Levels	Values
Species	3	SC SI SS
Temp	3	22 27 32
Humidity	2	70 90

Number of Observations Read	54
Number of Observations Used	54
Number of Events	1212
Number of Trials	2611

Dimensions	
Covariance Parameters	1
Columns in X	12
Columns in Z	0
Subjects (Blocks in V)	1
Max Obs per Subject	54

Optimization Information	
Optimization Technique	Newton-Raphson
Parameters in Optimization	6
Lower Boundaries	0
Upper Boundaries	0
Fixed Effects	Not Profiled

Iteration History					
Iteration	Restarts	Evaluations	Objective Function	Change	Max Gradient
0	0	4	224.2006742	.	2.742828
1	0	3	224.02266121	0.17801299	0.035223
2	0	3	224.02264939	0.00001181	5.2E-6

Convergence criterion (ABSGCONV=0.00001) satisfied.

Fit Statistics	
-2 Log Likelihood	448.05
AIC (smaller is better)	460.05
AICC (smaller is better)	461.83
BIC (smaller is better)	471.98
CAIC (smaller is better)	477.98
HQIC (smaller is better)	464.65
Pearson Chi-Square	219.63
Pearson Chi-Square / DF	4.58

Type III Tests of Fixed Effects

Effect	Num DF	Den DF	F Value	Pr > F
Temp	2	48	13.01	<.0001
Humidity	1	48	1.34	0.2524
Temp*Humidity	2	48	3.16	0.0515

Temp Least Squares Means							
Temp	Estimate	Standard Error	DF	t Value	Pr > |t|	Mean	Standard Error Mean
22	-0.5427	0.1659	48	-3.27	0.0020	0.3676	0.03858
27	0.3717	0.1358	48	2.74	0.0087	0.5919	0.03281
32	-0.5231	0.1567	48	-3.34	0.0016	0.3721	0.03661

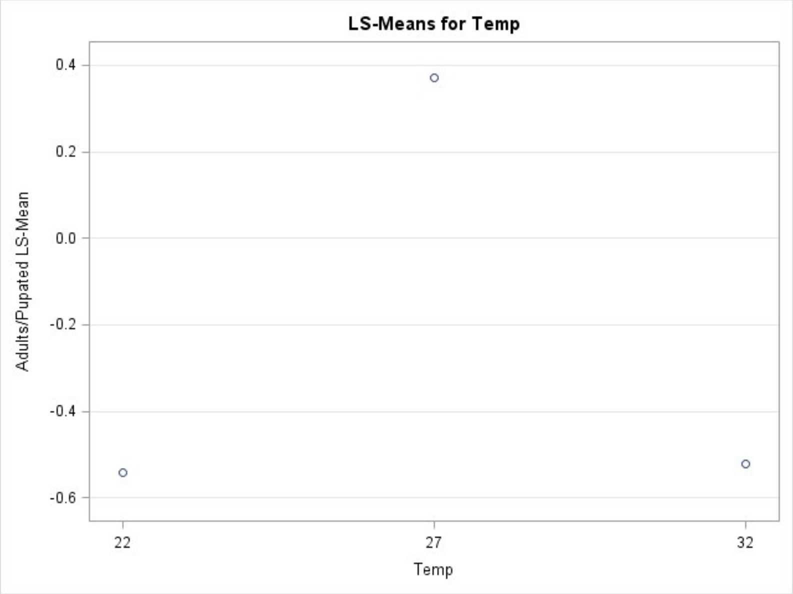

Differences of Temp Least Squares Means Adjustment for Multiple Comparisons: Tukey-Kramer							
Temp	Temp	Estimate	Standard Error	DF	t Value	Pr > |t|	Adj P
22	27	-0.9144	0.2144	48	-4.26	<.0001	0.0003
22	32	-0.01959	0.2282	48	-0.09	0.9320	0.9959
27	32	0.8948	0.2073	48	4.32	<.0001	0.0002

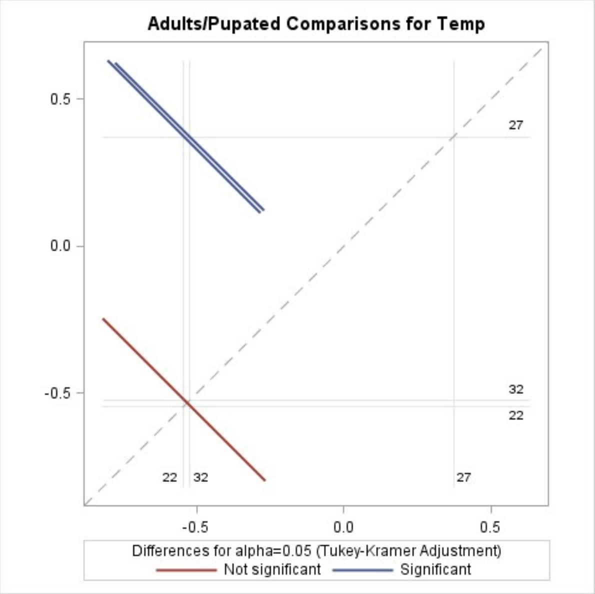

Temp*Humidity Least Squares Means

Temp	Humidity	Estimate	Standard Error	DF	t Value	Pr > |t|	Mean	Standard Error Mean
22	70	-0.9656	0.2635	48	-3.66	0.0006	0.2758	0.05262
22	90	-0.1199	0.2018	48	-0.59	0.5553	0.4701	0.05027
27	70	0.4653	0.1879	48	2.48	0.0169	0.6143	0.04452
27	90	0.2781	0.1961	48	1.42	0.1627	0.5691	0.04810
32	70	-0.5016	0.2198	48	-2.28	0.0270	0.3772	0.05164
32	90	-0.5447	0.2233	48	-2.44	0.0185	0.3671	0.05188

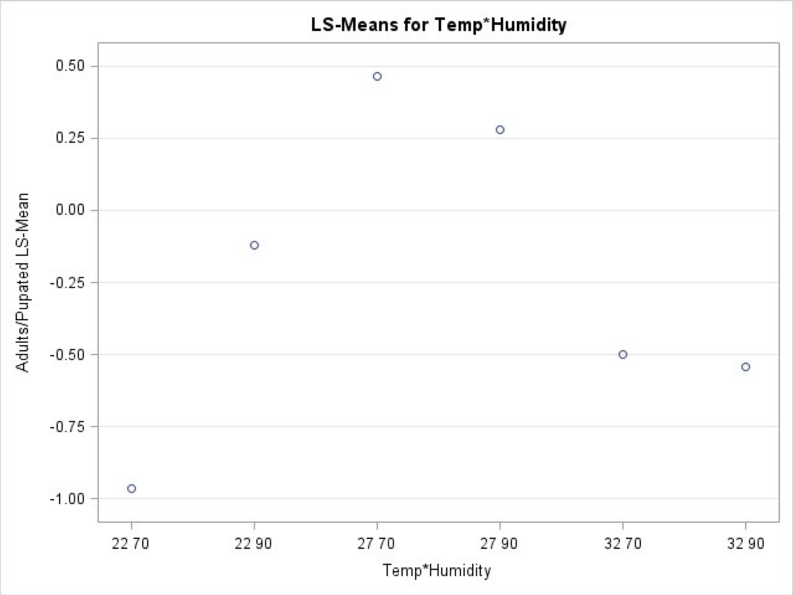

Differences of Temp*Humidity Least Squares Means
Adjustment for Multiple Comparisons: Tukey-Kramer

Temp	Humidity	Temp	Humidity	Estimate	Standard Error	DF	t Value	Pr > |t|	Adj P
22	70	22	90	-0.8457	0.3319	48	-2.55	0.0141	0.1307
22	70	27	70	-1.4309	0.3236	48	-4.42	<.0001	0.0008
22	70	27	90	-1.2437	0.3285	48	-3.79	0.0004	0.0054
22	70	32	70	-0.4640	0.3432	48	-1.35	0.1826	0.7545
22	70	32	90	-0.4209	0.3454	48	-1.22	0.2289	0.8257
22	90	27	70	-0.5851	0.2757	48	-2.12	0.0390	0.2936
22	90	27	90	-0.3979	0.2814	48	-1.41	0.1638	0.7185
22	90	32	70	0.3817	0.2984	48	1.28	0.2070	0.7948
22	90	32	90	0.4248	0.3010	48	1.41	0.1645	0.7199
27	70	27	90	0.1872	0.2716	48	0.69	0.4940	0.9823
27	70	32	70	0.9668	0.2892	48	3.34	0.0016	0.0189
27	70	32	90	1.0100	0.2918	48	3.46	0.0011	0.0137
27	90	32	70	0.7796	0.2946	48	2.65	0.0110	0.1058
27	90	32	90	0.8228	0.2972	48	2.77	0.0080	0.0804
32	70	32	90	0.04315	0.3134	48	0.14	0.8910	1.0000

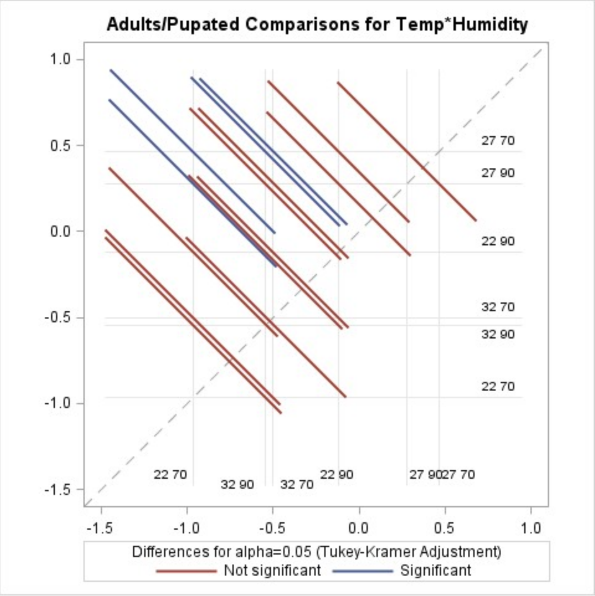
Adults/Pupated Comparisons for Temp*Humidity
27 70
27 90
22 90
32 70
32 90
22 70
22 70
32 90
32 70
22 90
27 90
27 70
Differences for alpha=0.05 (Tukey-Kramer Adjustment)
Not significant
Significant

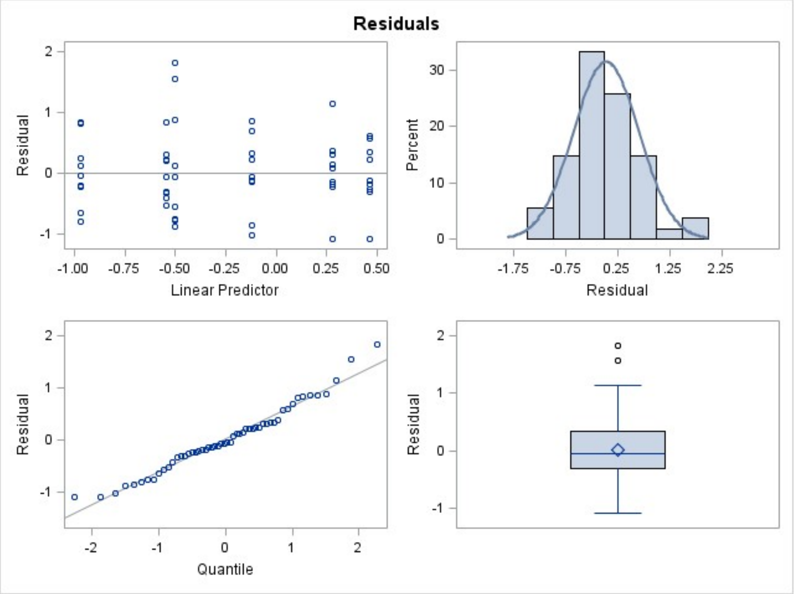
Residuals
Residual
Linear Predictor
Percent
Residual
Residual
Quantile
Residual

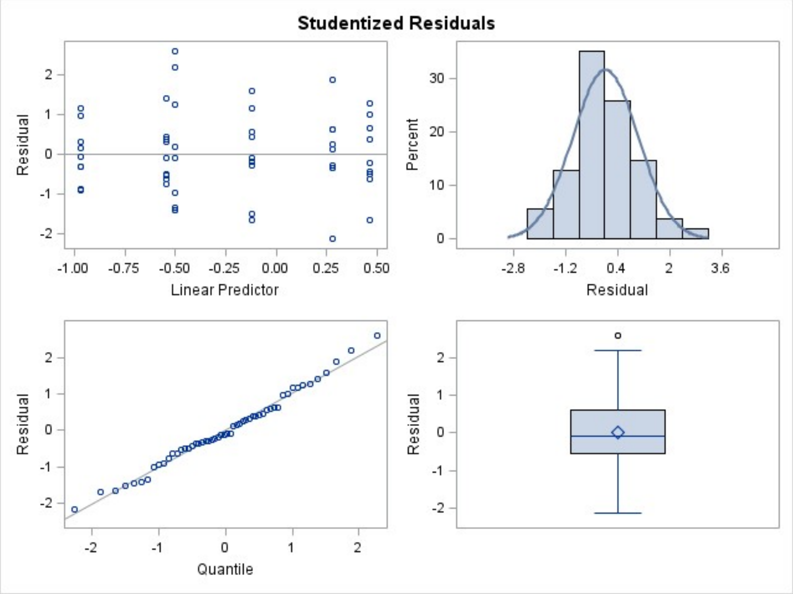
Studentized Residuals
Residual
Linear Predictor
-1.00 -0.75 -0.50 -0.25 0.00 0.25 0.50
Percent
Residual
-2.8 -1.2 0.4 2 3.6
Quantile
-2 -1 0 1 2

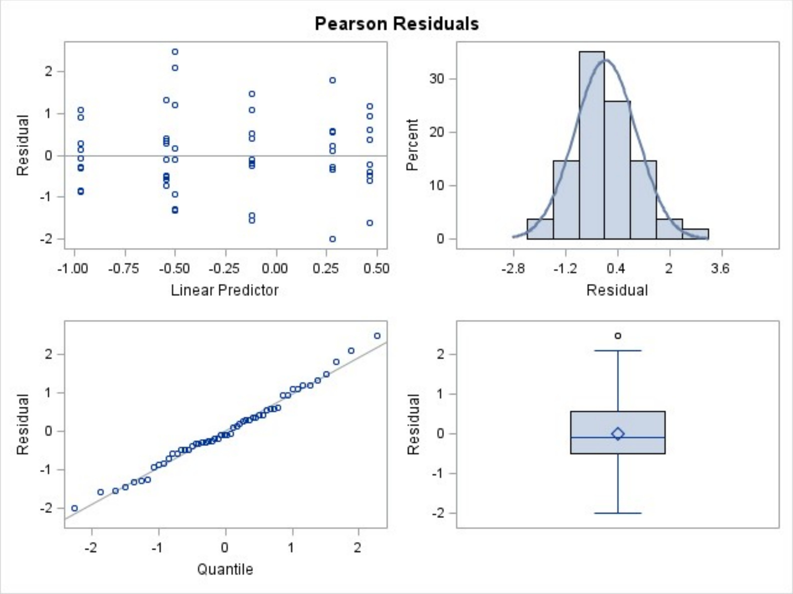
Pearson Residuals
Residual
Linear Predictor
-1.00 -0.75 -0.50 -0.25 0.00 0.25 0.50
Percent
Residual
-2.8 -1.2 0.4 2 3.6
Quantile
-2 -1 0 1 2

The SAS System

The GLIMMIX Procedure

Model Information	
Data Set	WORK.RAW
Response Variable (Events)	Hatched
Response Variable (Trials)	Eggs
Response Distribution	Binomial
Link Function	Logit
Variance Function	Default
Variance Matrix	Diagonal
Estimation Technique	Maximum Likelihood
Degrees of Freedom Method	Residual

Class Level Information		
Class	Levels	Values
Species	3	SC SI SS
Temp	4	22 27 32 v
Humidity	3	70 90 v

Number of Observations Read	63
Number of Observations Used	63
Number of Events	6317
Number of Trials	9450

Dimensions	
Covariance Parameters	1
Columns in X	39
Columns in Z	0
Subjects (Blocks in V)	1
Max Obs per Subject	63

Optimization Information	
Optimization Technique	Newton-Raphson
Parameters in Optimization	17
Lower Boundaries	0
Upper Boundaries	0
Fixed Effects	Not Profiled

Iteration History					
Iteration	Restarts	Evaluations	Objective Function	Change	Max Gradient
0	0	4	256.76447557	.	33.80603
1	0	3	256.08304257	0.68143300	0.387397
2	0	3	256.08282509	0.00021748	0.000145
3	0	3	256.08282509	0.00000000	3.46E-11

Convergence criterion (GCONV=1E-8) satisfied.

Fit Statistics	
-2 Log Likelihood	512.17
AIC (smaller is better)	546.17
AICC (smaller is better)	559.77
BIC (smaller is better)	582.60
CAIC (smaller is better)	599.60
HQIC (smaller is better)	560.50
Pearson Chi-Square	182.23
Pearson Chi-Square / DF	3.96

Type III Tests of Fixed Effects				
**Effect**	**Num DF**	**Den DF**	**F Value**	**Pr > F**
Species	2	46	0.07	0.9293
Temp	2	46	23.85	<.0001
Humidity	1	46	6.15	0.0169
Species*Temp	4	46	1.36	0.2635
Species*Humidity	2	46	2.47	0.0958
Temp*Humidity	2	46	20.08	<.0001

Species Least Squares Means							
**Species**	**Estimate**	**Standard Error**	**DF**	**t Value**	**Pr > |t|**	**Mean**	**Standard Error Mean**
SC	Non-est	.	.	.	.	Non-est	.
SI	Non-est	.	.	.	.	Non-est	.
SS	Non-est	.	.	.	.	Non-est	.

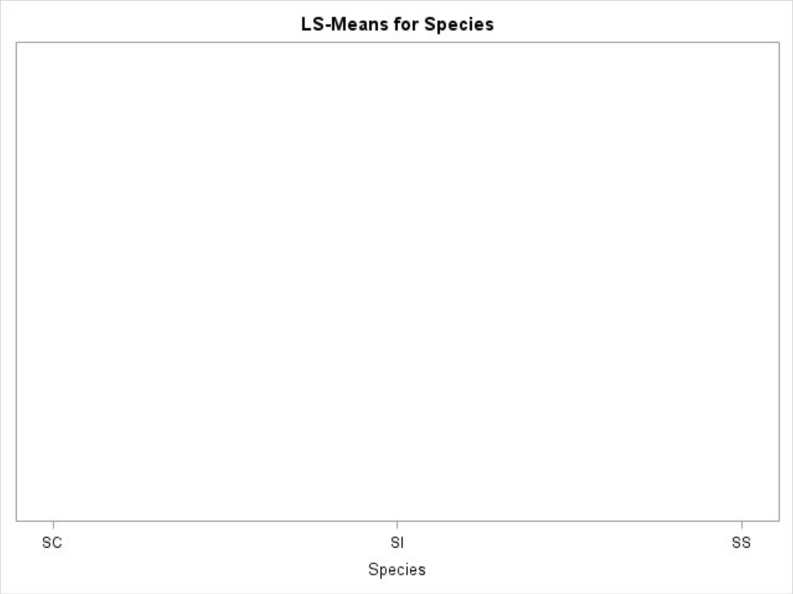

Differences of Species Least Squares Means Adjustment for Multiple Comparisons: Tukey							
**Species**	**Species**	**Estimate**	**Standard Error**	**DF**	**t Value**	**Pr > |t|**	**Adj P**
SC	SI	Non-est	.	.	.	.	.
SC	SS	Non-est	.	.	.	.	.
SI	SS	Non-est	.	.	.	.	.

Temp Least Squares Means							
**Temp**	**Estimate**	**Standard Error**	**DF**	**t Value**	**Pr > |t|**	**Mean**	**Standard Error Mean**
22	Non-est	.	.	.	.	Non-est	.
27	Non-est	.	.	.	.	Non-est	.
32	Non-est	.	.	.	.	Non-est	.
v	Non-est	.	.	.	.	Non-est	.

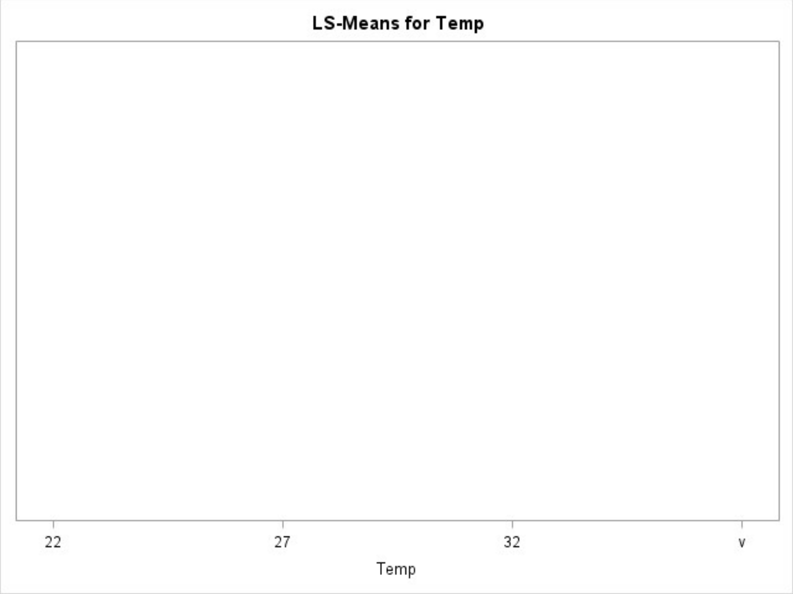

Differences of Temp Least Squares Means<br>Adjustment for Multiple Comparisons: Tukey							
**Temp**	**Temp**	**Estimate**	**Standard Error**	**DF**	**t Value**	**Pr > |t|**	**Adj P**
22	27	0.3137	0.1120	46	2.80	0.0074	0.0199
22	32	-0.5209	0.1232	46	-4.23	0.0001	0.0003
22	v	Non-est	.	.	.	.	.
27	32	-0.8346	0.1209	46	-6.90	<.0001	<.0001
27	v	Non-est	.	.	.	.	.
32	v	Non-est	.	.	.	.	.

Humidity Least Squares Means							
**Humidity**	**Estimate**	**Standard Error**	**DF**	**t Value**	**Pr > |t|**	**Mean**	**Standard Error Mean**
70	Non-est	.	.	.	.	Non-est	.
90	Non-est	.	.	.	.	Non-est	.
v	Non-est	.	.	.	.	Non-est	.

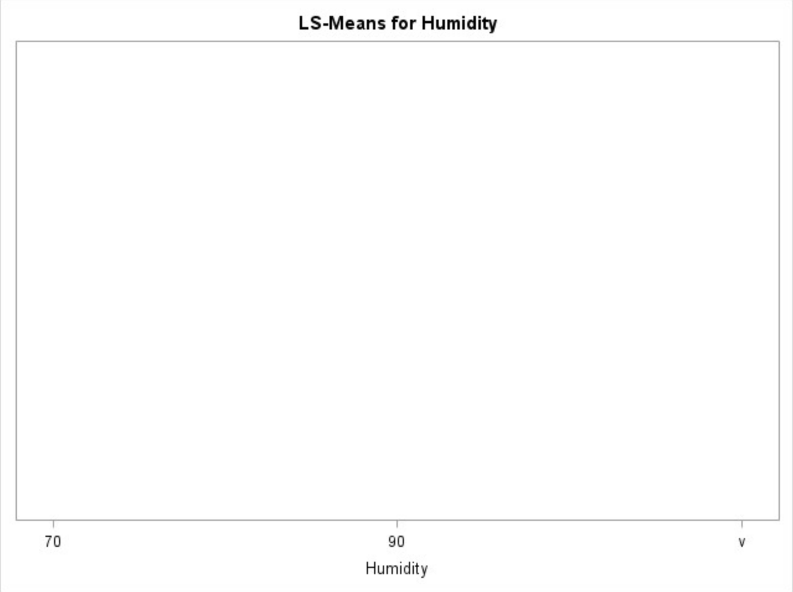

Differences of Humidity Least Squares Means
Adjustment for Multiple Comparisons: Tukey

Humidity	Humidity	Estimate	Standard Error	DF	t Value	Pr > |t|	Adj P
70	90	-0.2406	0.09702	46	-2.48	0.0169	0.0169
70	v	Non-est	.	.	.	.	.
90	v	Non-est	.	.	.	.	.

Temp*Humidity Least Squares Means

Temp	Humidity	Estimate	Standard Error	DF	t Value	Pr > |t|	Mean	Standard Error Mean
22	70	0.7809	0.1170	46	6.68	<.0001	0.6859	0.02520
22	90	0.3617	0.1117	46	3.24	0.0022	0.5894	0.02704
27	70	0.2451	0.1094	46	2.24	0.0299	0.5610	0.02694
27	90	0.2700	0.1097	46	2.46	0.0176	0.5671	0.02693
32	70	0.5340	0.1123	46	4.76	<.0001	0.6304	0.02615
32	90	1.6503	0.1480	46	11.15	<.0001	0.8389	0.02000
v	v	1.4951	0.1424	46	10.50	<.0001	0.8168	0.02131

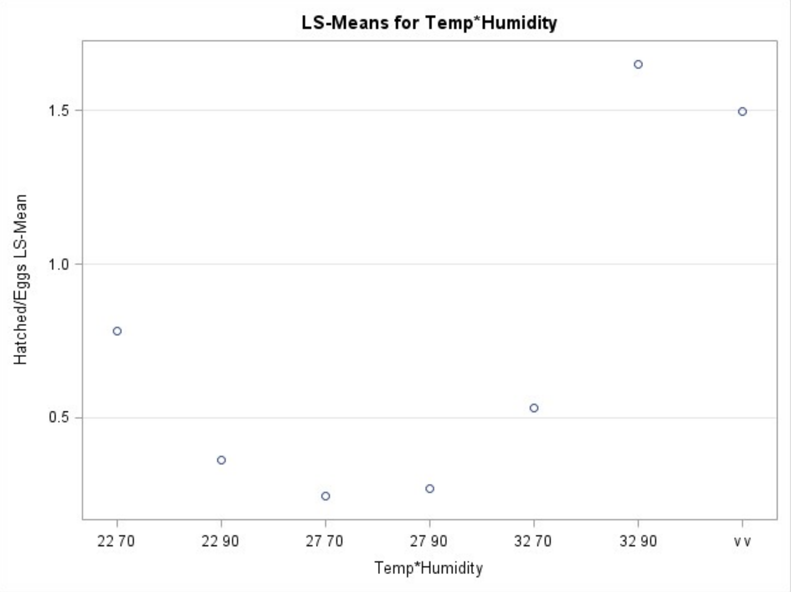

Differences of Temp*Humidity Least Squares Means
Adjustment for Multiple Comparisons: Tukey-Kramer

Temp	Humidity	Temp	Humidity	Estimate	Standard Error	DF	t Value	Pr > |t|	Adj P
22	70	22	90	0.4192	0.1617	46	2.59	0.0127	0.1520
22	70	27	70	0.5358	0.1602	46	3.35	0.0016	0.0255
22	70	27	90	0.5109	0.1603	46	3.19	0.0026	0.0385
22	70	32	70	0.2469	0.1621	46	1.52	0.1346	0.7298
22	70	32	90	-0.8694	0.1888	46	-4.61	<.0001	0.0006
22	70	v	v	-0.7142	0.1843	46	-3.88	0.0003	0.0058
22	90	27	70	0.1165	0.1564	46	0.75	0.4599	0.9888
22	90	27	90	0.09170	0.1566	46	0.59	0.5609	0.9969
22	90	32	70	-0.1724	0.1584	46	-1.09	0.2821	0.9284
22	90	32	90	-1.2887	0.1854	46	-6.95	<.0001	<.0001
22	90	v	v	-1.1334	0.1810	46	-6.26	<.0001	<.0001
27	70	27	90	-0.02483	0.1549	46	-0.16	0.8734	1.0000
27	70	32	70	-0.2889	0.1567	46	-1.84	0.0718	0.5271
27	70	32	90	-1.4052	0.1840	46	-7.64	<.0001	<.0001
27	70	v	v	-1.2500	0.1796	46	-6.96	<.0001	<.0001
27	90	32	70	-0.2641	0.1569	46	-1.68	0.0992	0.6307
27	90	32	90	-1.3804	0.1842	46	-7.49	<.0001	<.0001
27	90	v	v	-1.2251	0.1797	46	-6.82	<.0001	<.0001
32	70	32	90	-1.1163	0.1857	46	-6.01	<.0001	<.0001

32	70	v	v	-0.9611	0.1813	46	-5.30	<.0001	<.0001
32	90	v	v	0.1552	0.2054	46	0.76	0.4537	0.9879

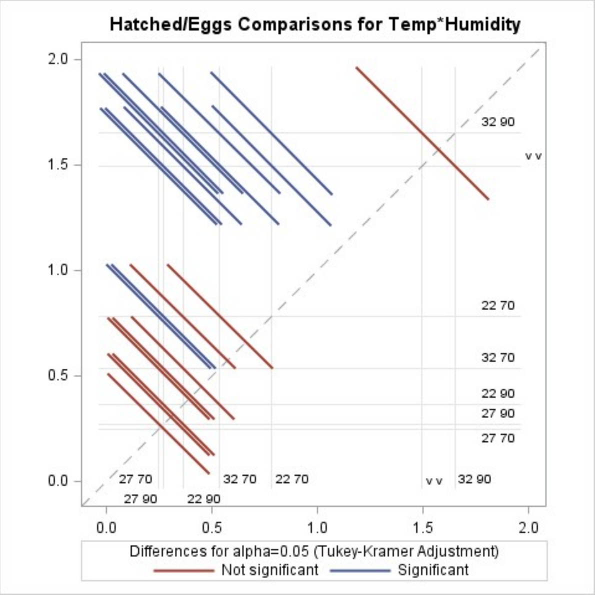

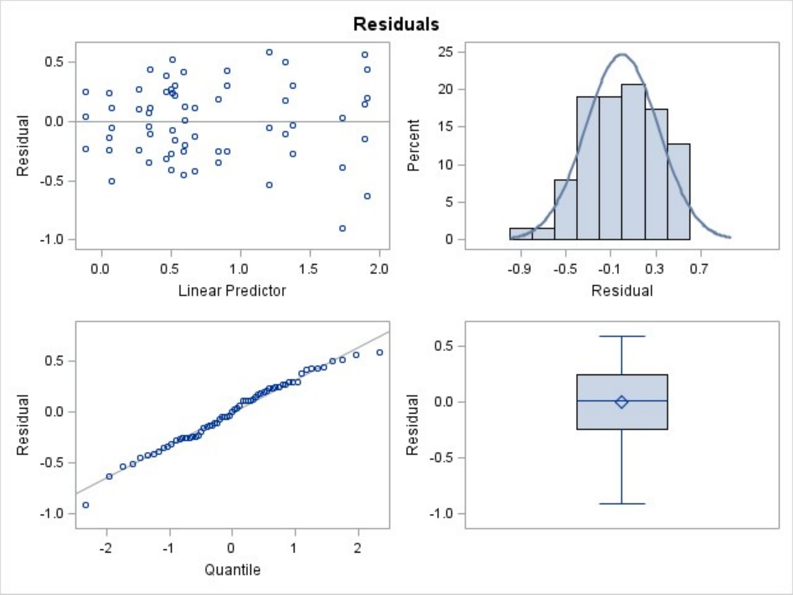

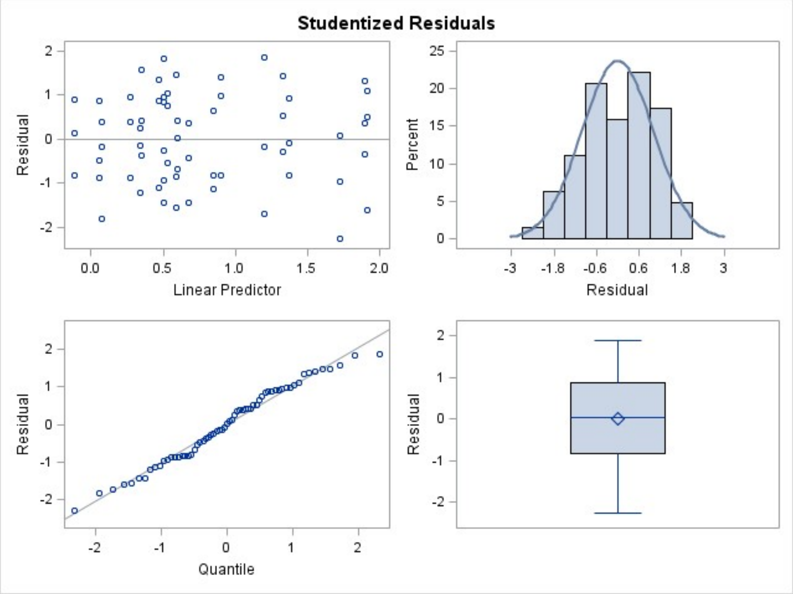
Studentized Residuals
Residual
Linear Predictor
Percent
Residual
Residual
Quantile
Residual

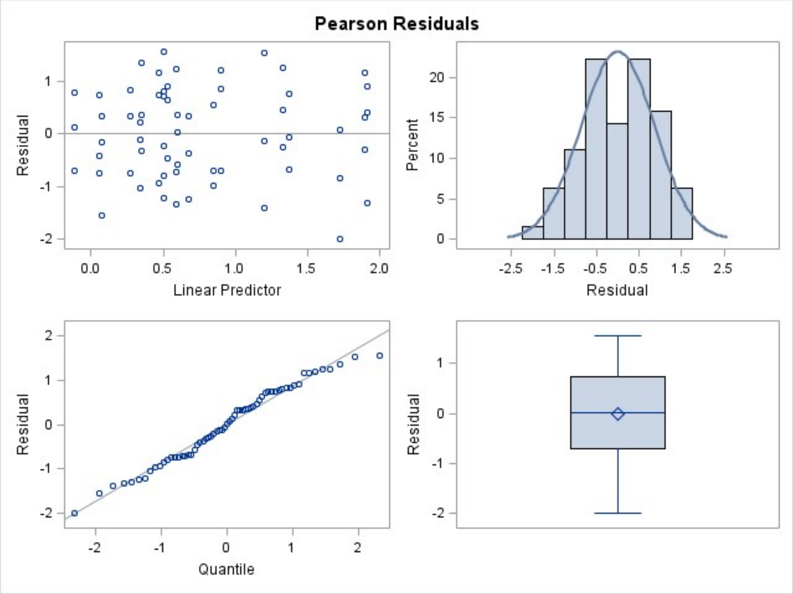
Pearson Residuals
Residual
Linear Predictor
Percent
Residual
Residual
Quantile
Residual

The SAS System

The GLIMMIX Procedure

Model Information	
Data Set	WORK.RAW
Response Variable (Events)	Pupated
Response Variable (Trials)	Hatched
Response Distribution	Binomial
Link Function	Logit
Variance Function	Default
Variance Matrix	Diagonal
Estimation Technique	Maximum Likelihood
Degrees of Freedom Method	Residual

Class Level Information		
Class	Levels	Values
Species	3	SC SI SS
Temp	4	22 27 32 v
Humidity	3	70 90 v

Number of Observations Read	63
Number of Observations Used	63
Number of Events	3415
Number of Trials	6317

Dimensions	
Covariance Parameters	1
Columns in X	39
Columns in Z	0
Subjects (Blocks in V)	1
Max Obs per Subject	63

Optimization Information	
Optimization Technique	Newton-Raphson
Parameters in Optimization	17
Lower Boundaries	0
Upper Boundaries	0
Fixed Effects	Not Profiled

Iteration History					
Iteration	Restarts	Evaluations	Objective Function	Change	Max Gradient
0	0	4	303.79646797	.	29.36716
1	0	3	301.36173905	2.43472892	0.782257
2	0	3	301.3589937	0.00274535	0.001133
3	0	3	301.35899369	0.00000001	2.812E-9

Convergence criterion (GCONV=1E-8) satisfied.

Fit Statistics	
-2 Log Likelihood	602.72
AIC (smaller is better)	636.72
AICC (smaller is better)	650.32
BIC (smaller is better)	673.15
CAIC (smaller is better)	690.15
HQIC (smaller is better)	651.05
Pearson Chi-Square	290.51
Pearson Chi-Square / DF	6.32

Type III Tests of Fixed Effects				
**Effect**	**Num DF**	**Den DF**	**F Value**	**Pr > F**
**Species**	2	46	0.00	0.9957
**Temp**	2	46	20.24	<.0001
**Humidity**	1	46	0.02	0.8804
**Species*Temp**	4	46	1.27	0.2941
**Species*Humidity**	2	46	0.34	0.7158
**Temp*Humidity**	2	46	9.61	0.0003

Temp Least Squares Means							
**Temp**	**Estimate**	**Standard Error**	**DF**	**t Value**	**Pr > |t|**	**Mean**	**Standard Error Mean**
22	Non-est	.	.	.	.	Non-est	.
27	Non-est	.	.	.	.	Non-est	.
32	Non-est	.	.	.	.	Non-est	.
v	Non-est	.	.	.	.	Non-est	.

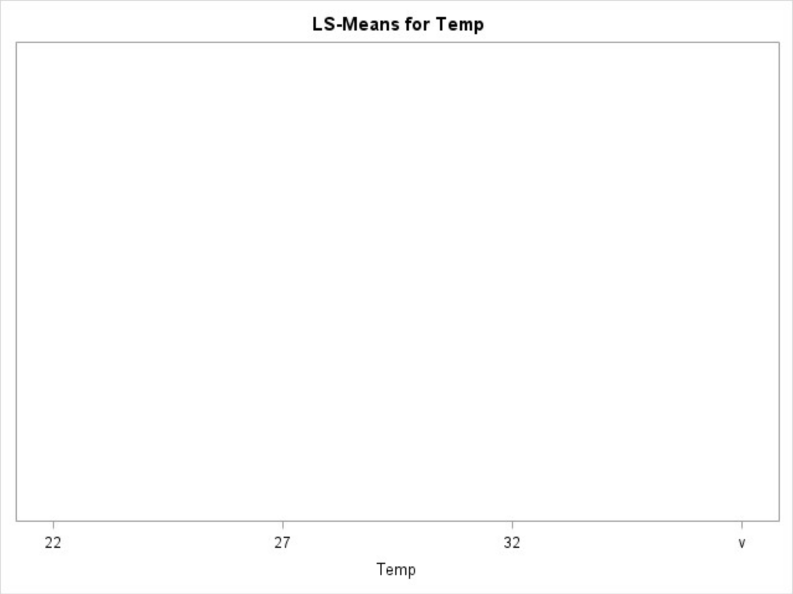

Differences of Temp Least Squares Means Adjustment for Multiple Comparisons: Tukey							
**Temp**	**Temp**	**Estimate**	**Standard Error**	**DF**	**t Value**	**Pr > |t|**	**Adj P**
22	27	-0.8985	0.1876	46	-4.79	<.0001	<.0001
22	32	0.2254	0.1721	46	1.31	0.1968	0.3972
22	v	Non-est	.	.	.	.	.
27	32	1.1239	0.1823	46	6.16	<.0001	<.0001
27	v	Non-est	.	.	.	.	.
32	v	Non-est	.	.	.	.	.

Temp*Humidity Least Squares Means								
**Temp**	**Humidity**	**Estimate**	**Standard Error**	**DF**	**t Value**	**Pr > |t|**	**Mean**	**Standard Error Mean**
22	70	-0.5832	0.1735	46	-3.36	0.0016	0.3582	0.03989
22	90	0.2889	0.1821	46	1.59	0.1195	0.5717	0.04459
27	70	0.9583	0.2047	46	4.68	<.0001	0.7228	0.04101
27	90	0.5445	0.1894	46	2.87	0.0061	0.6329	0.04401
32	70	-0.1098	0.1734	46	-0.63	0.5295	0.4726	0.04321
32	90	-0.6352	0.1587	46	-4.00	0.0002	0.3463	0.03592
v	v	1.0082	0.1716	46	5.87	<.0001	0.7327	0.03362

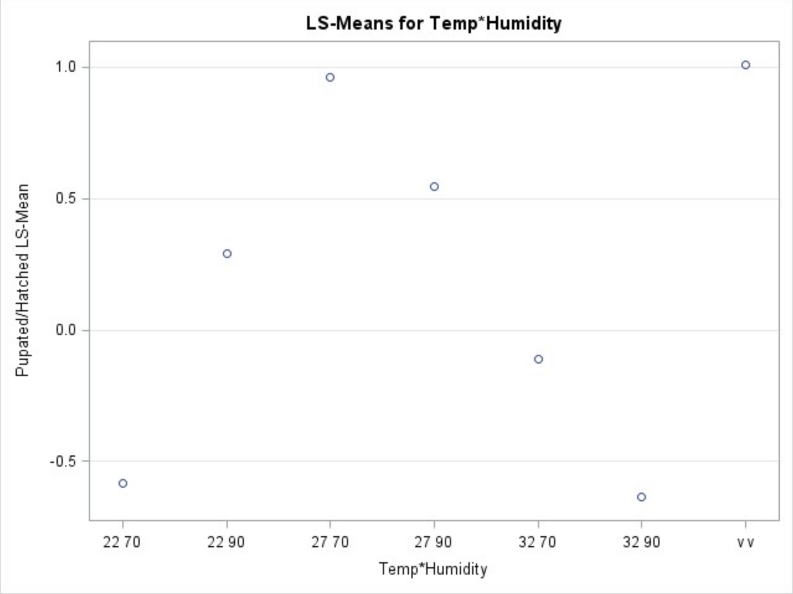

Differences of Temp*Humidity Least Squares Means
Adjustment for Multiple Comparisons: Tukey-Kramer

Temp	Humidity	Temp	Humidity	Estimate	Standard Error	DF	t Value	Pr > |t|	Adj P
22	70	22	90	-0.8721	0.2516	46	-3.47	0.0012	0.0184
22	70	27	70	-1.5414	0.2683	46	-5.74	<.0001	<.0001
22	70	27	90	-1.1277	0.2568	46	-4.39	<.0001	0.0012
22	70	32	70	-0.4733	0.2453	46	-1.93	0.0598	0.4720
22	70	32	90	0.05205	0.2351	46	0.22	0.8258	1.0000
22	70	v	v	-1.5914	0.2441	46	-6.52	<.0001	<.0001
22	90	27	70	-0.6694	0.2738	46	-2.44	0.0184	0.2039
22	90	27	90	-0.2556	0.2624	46	-0.97	0.3352	0.9571
22	90	32	70	0.3987	0.2516	46	1.58	0.1199	0.6925
22	90	32	90	0.9241	0.2412	46	3.83	0.0004	0.0066
22	90	v	v	-0.7193	0.2503	46	-2.87	0.0061	0.0822
27	70	27	90	0.4138	0.2789	46	1.48	0.1448	0.7530
27	70	32	70	1.0681	0.2682	46	3.98	0.0002	0.0042
27	70	32	90	1.5935	0.2588	46	6.16	<.0001	<.0001
27	70	v	v	-0.04995	0.2671	46	-0.19	0.8525	1.0000
27	90	32	70	0.6543	0.2569	46	2.55	0.0143	0.1669
27	90	32	90	1.1797	0.2470	46	4.78	<.0001	0.0004
27	90	v	v	-0.4637	0.2556	46	-1.81	0.0762	0.5456
32	70	32	90	0.5254	0.2349	46	2.24	0.0302	0.2968
32	70	v	v	-1.1181	0.2440	46	-4.58	<.0001	0.0007
32	90	v	v	-1.6434	0.2338	46	-7.03	<.0001	<.0001

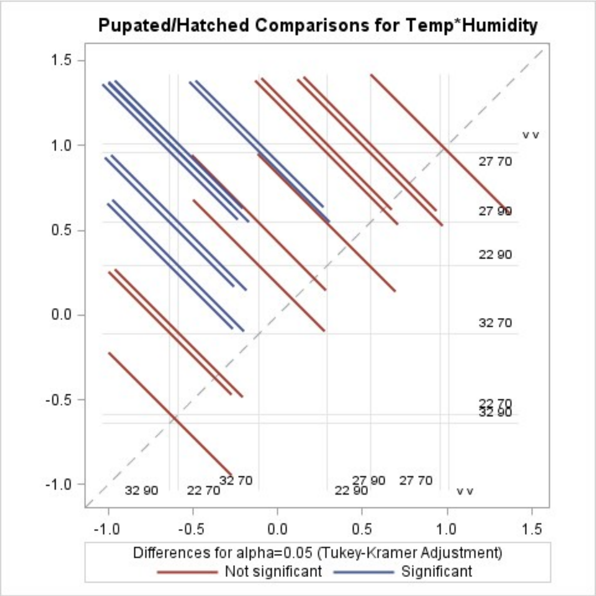
Pupated/Hatched Comparisons for Temp*Humidity
27 70
27 90
22 90
32 70
22 70
32 90
Differences for alpha=0.05 (Tukey-Kramer Adjustment)
Not significant
Significant

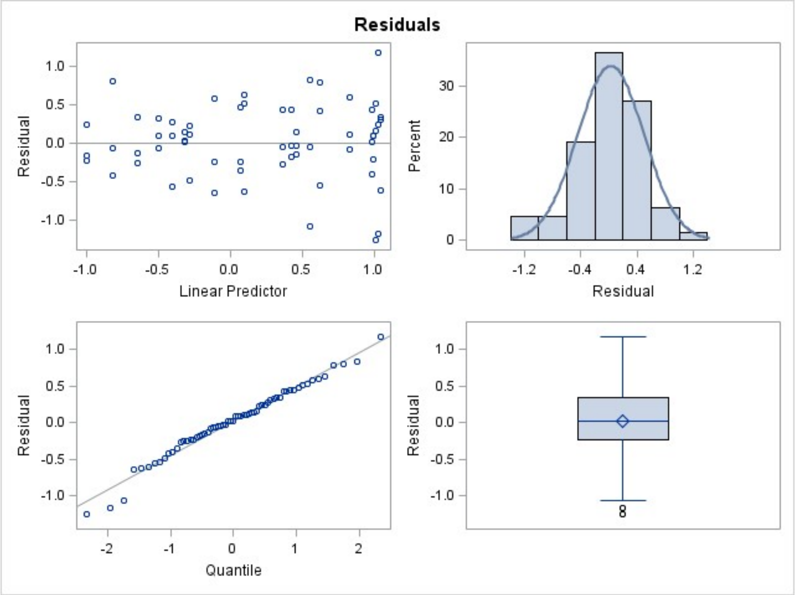
Residuals
Residual
Linear Predictor
Percent
Residual
Quantile

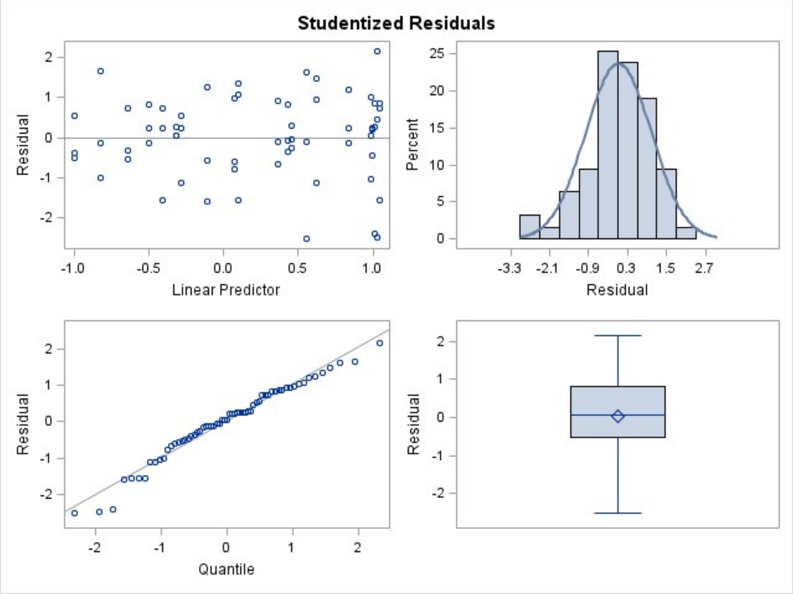
Studentized Residuals
Residual
Linear Predictor
Percent
Residual
Quantile
-3.3 -2.1 -0.9 0.3 1.5 2.7

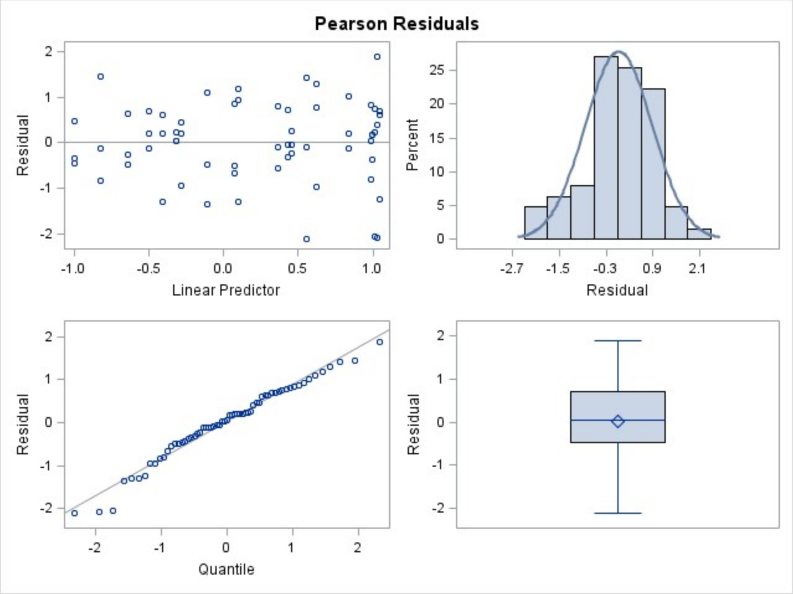
Pearson Residuals
Residual
Linear Predictor
Percent
Residual
Quantile
-2.7 -1.5 -0.3 0.9 2.1

The SAS System

The GLIMMIX Procedure

Model Information	
Data Set	WORK.RAW
Response Variable (Events)	Pupated
Response Variable (Trials)	Hatched
Response Distribution	Binomial
Link Function	Logit
Variance Function	Default
Variance Matrix	Diagonal
Estimation Technique	Maximum Likelihood
Degrees of Freedom Method	Residual

Class Level Information		
Class	Levels	Values
Species	3	SC SI SS
Temp	4	22 27 32 v
Humidity	3	70 90 v

Number of Observations Read	63
Number of Observations Used	63
Number of Events	3415
Number of Trials	6317

Dimensions	
Covariance Parameters	1
Columns in X	15
Columns in Z	0
Subjects (Blocks in V)	1
Max Obs per Subject	63

Optimization Information	
Optimization Technique	Newton-Raphson
Parameters in Optimization	7
Lower Boundaries	0
Upper Boundaries	0
Fixed Effects	Not Profiled

Iteration History					
Iteration	Restarts	Evaluations	Objective Function	Change	Max Gradient
0	0	4	322.99153731	.	27.43693
1	0	3	321.05760753	1.93392977	0.636055
2	0	3	321.05644671	0.00116082	0.000512
3	0	3	321.05644671	0.00000000	3.81E-10

Convergence criterion (GCONV=1E-8) satisfied.

Fit Statistics	
-2 Log Likelihood	642.11
AIC (smaller is better)	656.11
AICC (smaller is better)	658.15
BIC (smaller is better)	671.11
CAIC (smaller is better)	678.11
HQIC (smaller is better)	662.01
Pearson Chi-Square	326.14
Pearson Chi-Square / DF	5.82

Type III Tests of Fixed Effects				
**Effect**	**Num DF**	**Den DF**	**F Value**	**Pr > F**
**Temp**	2	56	22.03	<.0001
**Humidity**	1	56	0.02	0.8958
**Temp*Humidity**	2	56	10.33	0.0002

Temp Least Squares Means							
**Temp**	**Estimate**	**Standard Error**	**DF**	**t Value**	**Pr > |t|**	**Mean**	**Standard Error Mean**
22	Non-est	.	.	.	.	Non-est	.
27	Non-est	.	.	.	.	Non-est	.
32	Non-est	.	.	.	.	Non-est	.
v	Non-est	.	.	.	.	Non-est	.

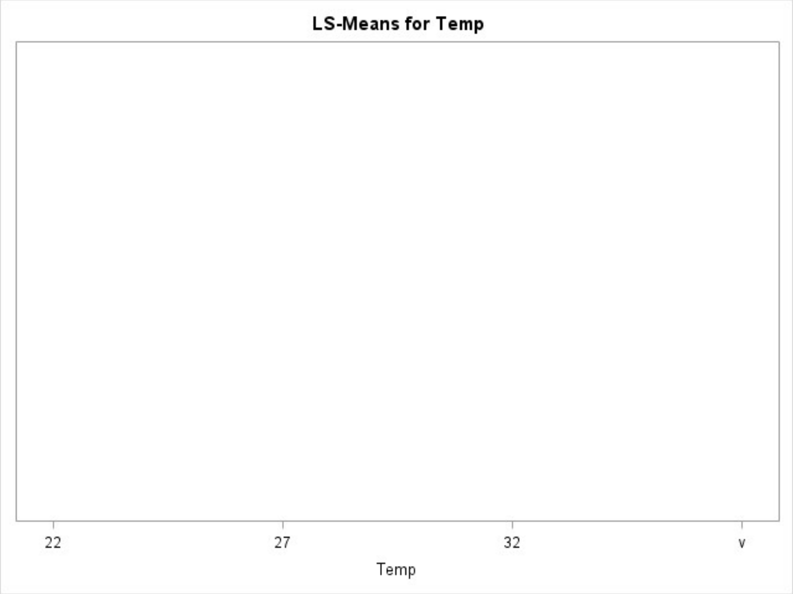

Differences of Temp Least Squares Means Adjustment for Multiple Comparisons: Tukey							
**Temp**	**Temp**	**Estimate**	**Standard Error**	**DF**	**t Value**	**Pr > |t|**	**Adj P**
22	27	-0.9098	0.1793	56	-5.07	<.0001	<.0001
22	32	0.2063	0.1639	56	1.26	0.2134	0.4244
22	v	Non-est	.	.	.	.	.
27	32	1.1161	0.1742	56	6.41	<.0001	<.0001
27	v	Non-est	.	.	.	.	.
32	v	Non-est	.	.	.	.	.

Temp*Humidity Least Squares Means								
**Temp**	**Humidity**	**Estimate**	**Standard Error**	**DF**	**t Value**	**Pr > |t|**	**Mean**	**Standard Error Mean**
22	70	-0.5895	0.1656	56	-3.56	0.0008	0.3568	0.03801
22	90	0.2767	0.1730	56	1.60	0.1155	0.5687	0.04244
27	70	0.9573	0.1959	56	4.89	<.0001	0.7226	0.03927
27	90	0.5494	0.1811	56	3.03	0.0037	0.6340	0.04203
32	70	-0.1059	0.1657	56	-0.64	0.5255	0.4736	0.04131
32	90	-0.6196	0.1506	56	-4.11	0.0001	0.3499	0.03425
v	v	1.0094	0.1647	56	6.13	<.0001	0.7329	0.03224

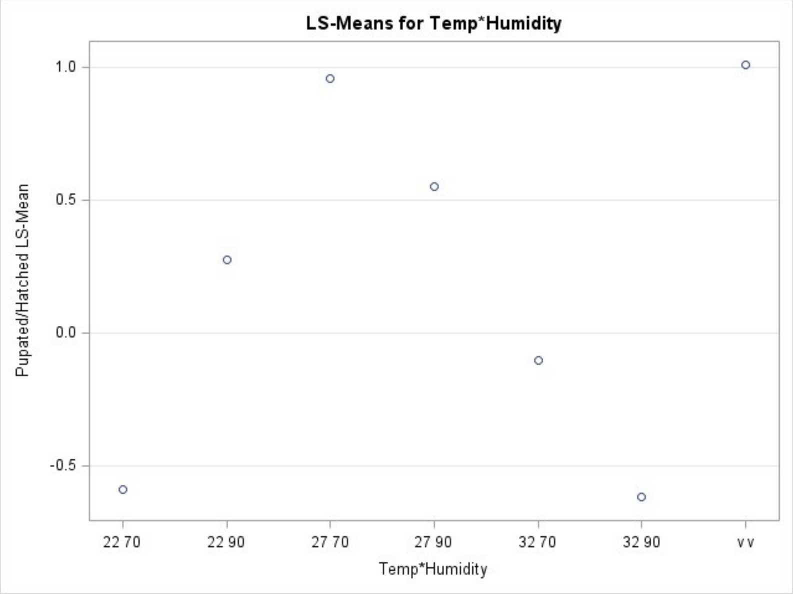

Differences of Temp*Humidity Least Squares Means
Adjustment for Multiple Comparisons: Tukey-Kramer

Temp	Humidity	Temp	Humidity	Estimate	Standard Error	DF	t Value	Pr > |t|	Adj P
22	70	22	90	-0.8661	0.2395	56	-3.62	0.0006	0.0108
22	70	27	70	-1.5468	0.2565	56	-6.03	<.0001	<.0001
22	70	27	90	-1.1388	0.2454	56	-4.64	<.0001	0.0004
22	70	32	70	-0.4836	0.2343	56	-2.06	0.0436	0.3877
22	70	32	90	0.03015	0.2239	56	0.13	0.8933	1.0000
22	70	v	v	-1.5989	0.2336	56	-6.85	<.0001	<.0001
22	90	27	70	-0.6807	0.2614	56	-2.60	0.0118	0.1444
22	90	27	90	-0.2727	0.2505	56	-1.09	0.2810	0.9289
22	90	32	70	0.3825	0.2396	56	1.60	0.1160	0.6849
22	90	32	90	0.8963	0.2294	56	3.91	0.0003	0.0045
22	90	v	v	-0.7328	0.2389	56	-3.07	0.0033	0.0488
27	70	27	90	0.4080	0.2668	56	1.53	0.1319	0.7263
27	70	32	70	1.0632	0.2566	56	4.14	0.0001	0.0021
27	70	32	90	1.5770	0.2471	56	6.38	<.0001	<.0001
27	70	v	v	-0.05209	0.2559	56	-0.20	0.8395	1.0000
27	90	32	70	0.6552	0.2455	56	2.67	0.0099	0.1255
27	90	32	90	1.1690	0.2356	56	4.96	<.0001	0.0001
27	90	v	v	-0.4601	0.2448	56	-1.88	0.0654	0.5022
32	70	32	90	0.5138	0.2239	56	2.29	0.0255	0.2648
32	70	v	v	-1.1153	0.2336	56	-4.77	<.0001	0.0003
32	90	v	v	-1.6290	0.2232	56	-7.30	<.0001	<.0001

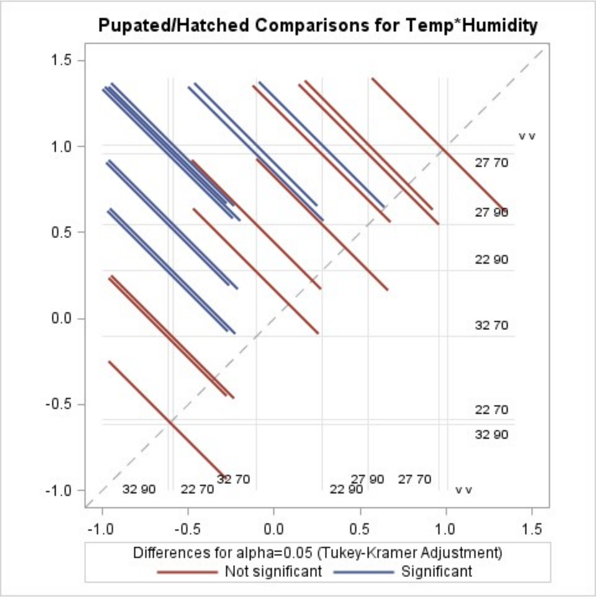
Pupated/Hatched Comparisons for Temp*Humidity
27 70
27 90
22 90
32 70
22 70
32 90
32 90
22 70
32 70
22 90
27 90
27 70
v v
Differences for alpha=0.05 (Tukey-Kramer Adjustment)
Not significant
Significant

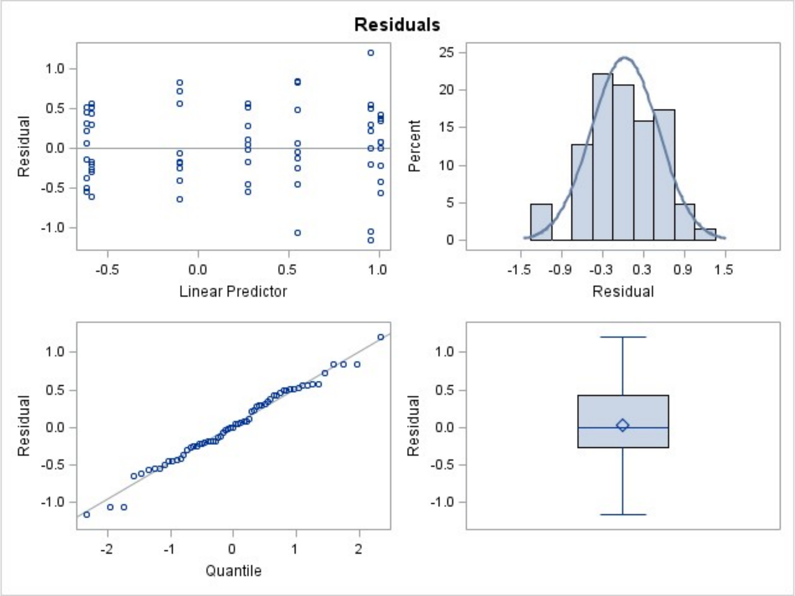
Residuals
Residual
Linear Predictor
Percent
Residual
Residual
Quantile
Residual

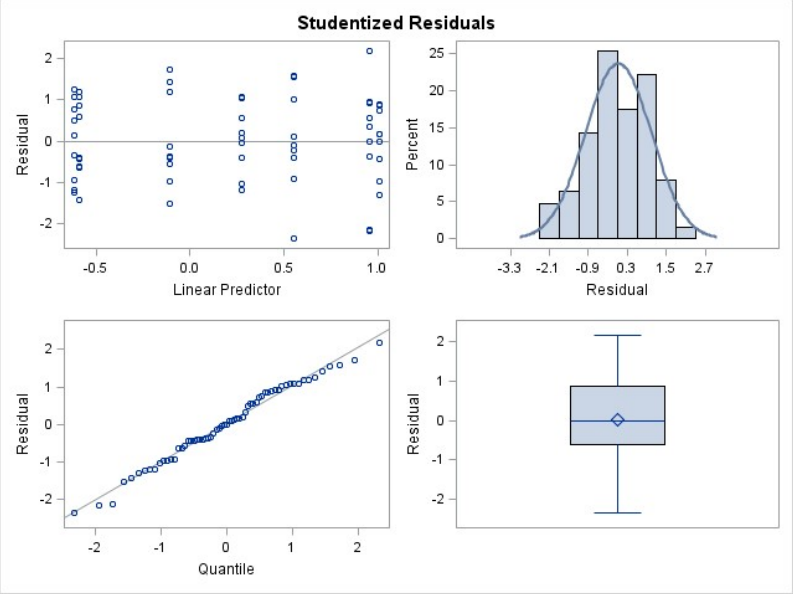
Studentized Residuals
Residual
Linear Predictor
Percent
Residual
Quantile

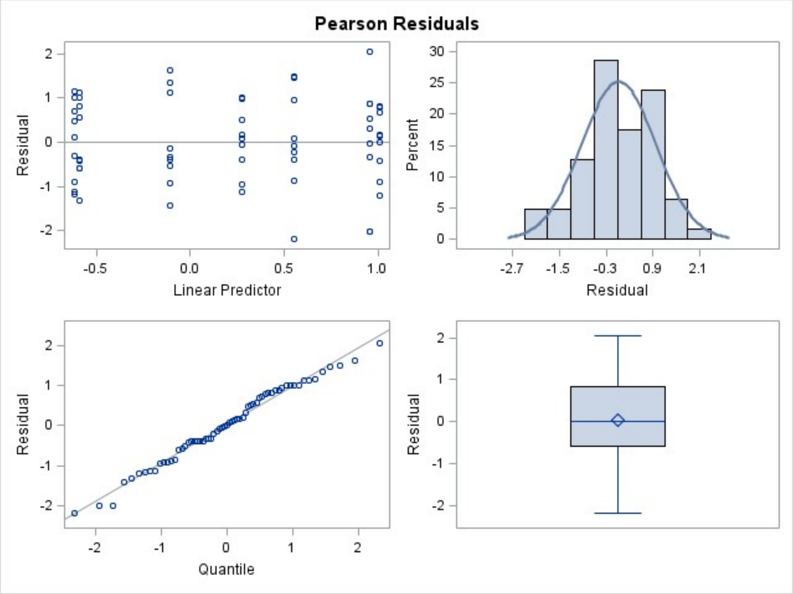
Pearson Residuals
Residual
Linear Predictor
Percent
Residual
Quantile

The SAS System

The GLIMMIX Procedure

Model Information	
Data Set	WORK.RAW
Response Variable (Events)	Adults
Response Variable (Trials)	Pupated
Response Distribution	Binomial
Link Function	Logit
Variance Function	Default
Variance Matrix	Diagonal
Estimation Technique	Maximum Likelihood
Degrees of Freedom Method	Residual

Class Level Information		
Class	Levels	Values
Species	3	SC SI SS
Temp	4	22 27 32 v
Humidity	3	70 90 v

Number of Observations Read	63
Number of Observations Used	63
Number of Events	1836
Number of Trials	3415

Dimensions	
Covariance Parameters	1
Columns in X	39
Columns in Z	0
Subjects (Blocks in V)	1
Max Obs per Subject	63

Optimization Information	
Optimization Technique	Newton-Raphson
Parameters in Optimization	17
Lower Boundaries	0
Upper Boundaries	0
Fixed Effects	Not Profiled

Iteration History					
Iteration	Restarts	Evaluations	Objective Function	Change	Max Gradient
0	0	4	223.55141412	.	3.305801
1	0	3	223.11068847	0.44072565	0.034809
2	0	3	223.11062393	0.00006453	0.00002
3	0	3	223.11062393	0.00000000	4.67E-12

Convergence criterion (GCONV=1E-8) satisfied.

Fit Statistics	
-2 Log Likelihood	446.22
AIC (smaller is better)	480.22
AICC (smaller is better)	493.82
BIC (smaller is better)	516.65
CAIC (smaller is better)	533.65
HQIC (smaller is better)	494.55
Pearson Chi-Square	175.77
Pearson Chi-Square / DF	3.82

Type III Tests of Fixed Effects				
**Effect**	**Num DF**	**Den DF**	**F Value**	**Pr > F**
**Species**	2	46	1.33	0.2739
**Temp**	2	46	14.48	<.0001
**Humidity**	1	46	1.96	0.1680
**Species*Temp**	4	46	1.89	0.1275
**Species*Humidity**	2	46	0.50	0.6082
**Temp*Humidity**	2	46	3.70	0.0322

Temp Least Squares Means							
**Temp**	**Estimate**	**Standard Error**	**DF**	**t Value**	**Pr > |t|**	**Mean**	**Standard Error Mean**
22	Non-est	.	.	.	.	Non-est	.
27	Non-est	.	.	.	.	Non-est	.
32	Non-est	.	.	.	.	Non-est	.
v	Non-est	.	.	.	.	Non-est	.

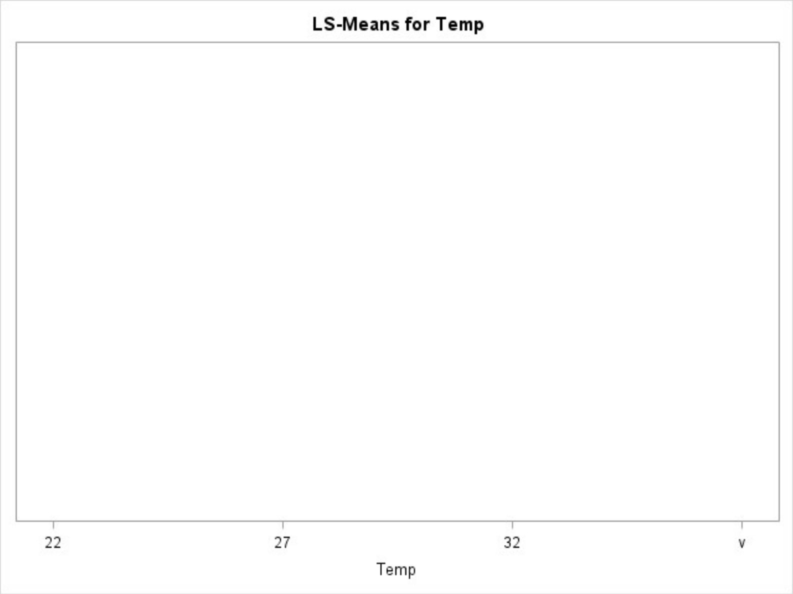

Differences of Temp Least Squares Means Adjustment for Multiple Comparisons: Tukey							
**Temp**	**Temp**	**Estimate**	**Standard Error**	**DF**	**t Value**	**Pr > |t|**	**Adj P**
22	27	-0.9018	0.1969	46	-4.58	<.0001	0.0001
22	32	-0.04120	0.2119	46	-0.19	0.8467	0.9794
22	v	Non-est	.	.	.	.	.
27	32	0.8606	0.1933	46	4.45	<.0001	0.0002
27	v	Non-est	.	.	.	.	.
32	v	Non-est	.	.	.	.	.

Temp*Humidity Least Squares Means								
**Temp**	**Humidity**	**Estimate**	**Standard Error**	**DF**	**t Value**	**Pr > |t|**	**Mean**	**Standard Error Mean**
22	70	-0.9713	0.2415	46	-4.02	0.0002	0.2746	0.04812
22	90	-0.1002	0.1863	46	-0.54	0.5931	0.4750	0.04645
27	70	0.4465	0.1738	46	2.57	0.0135	0.6098	0.04136
27	90	0.2856	0.1808	46	1.58	0.1210	0.5709	0.04429
32	70	-0.4848	0.2078	46	-2.33	0.0241	0.3811	0.04902
32	90	-0.5043	0.2091	46	-2.41	0.0199	0.3765	0.04910
v	v	1.2438	0.1661	46	7.49	<.0001	0.7762	0.02885

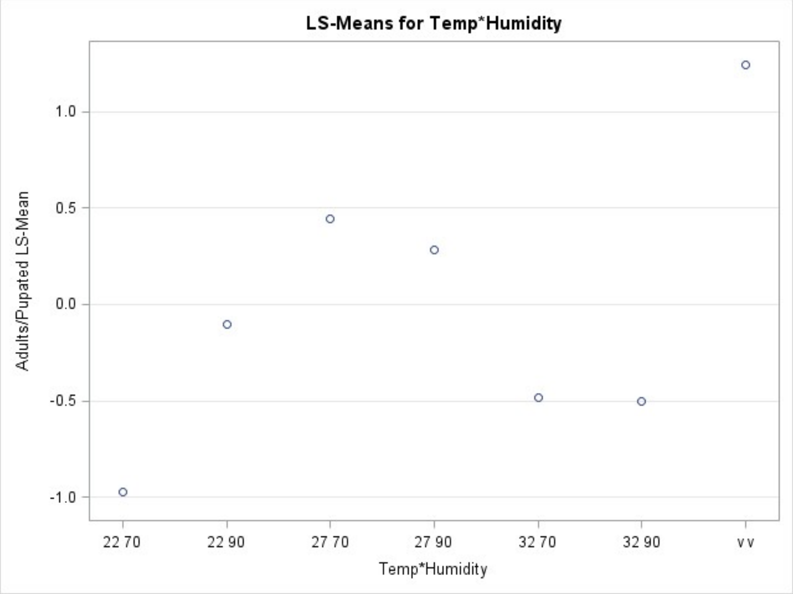

Differences of Temp*Humidity Least Squares Means
Adjustment for Multiple Comparisons: Tukey-Kramer

Temp	Humidity	Temp	Humidity	Estimate	Standard Error	DF	t Value	Pr > |t|	Adj P
22	70	22	90	-0.8711	0.3055	46	-2.85	0.0065	0.0867
22	70	27	70	-1.4178	0.2974	46	-4.77	<.0001	0.0004
22	70	27	90	-1.2569	0.3014	46	-4.17	0.0001	0.0024
22	70	32	70	-0.4865	0.3188	46	-1.53	0.1338	0.7280
22	70	32	90	-0.4670	0.3191	46	-1.46	0.1501	0.7643
22	70	v	v	-2.2151	0.2932	46	-7.56	<.0001	<.0001
22	90	27	70	-0.5467	0.2547	46	-2.15	0.0371	0.3440
22	90	27	90	-0.3859	0.2594	46	-1.49	0.1438	0.7507
22	90	32	70	0.3846	0.2792	46	1.38	0.1750	0.8107
22	90	32	90	0.4041	0.2798	46	1.44	0.1555	0.7751
22	90	v	v	-1.3440	0.2496	46	-5.39	<.0001	<.0001
27	70	27	90	0.1609	0.2513	46	0.64	0.5252	0.9950
27	70	32	70	0.9313	0.2709	46	3.44	0.0013	0.0199
27	70	32	90	0.9508	0.2717	46	3.50	0.0010	0.0168
27	70	v	v	-0.7973	0.2405	46	-3.32	0.0018	0.0276
27	90	32	70	0.7705	0.2754	46	2.80	0.0075	0.0979
27	90	32	90	0.7899	0.2760	46	2.86	0.0063	0.0846
27	90	v	v	-0.9582	0.2455	46	-3.90	0.0003	0.0053
32	70	32	90	0.01944	0.2945	46	0.07	0.9476	1.0000
32	70	v	v	-1.7287	0.2661	46	-6.50	<.0001	<.0001
32	90	v	v	-1.7481	0.2671	46	-6.55	<.0001	<.0001

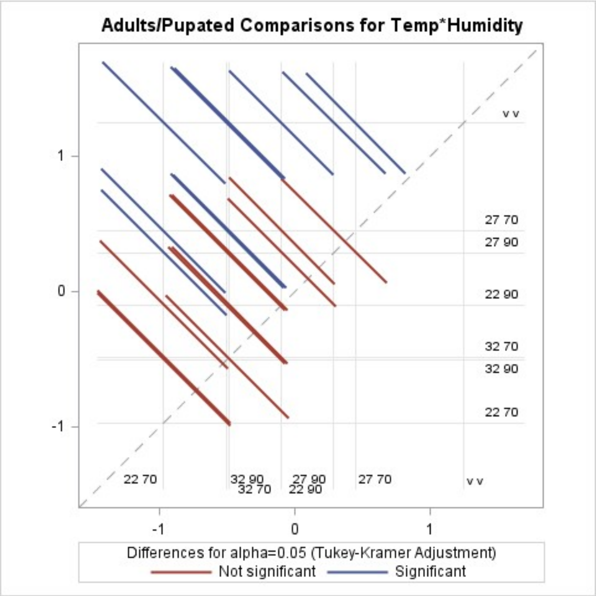
Adults/Pupated Comparisons for Temp*Humidity
27 70
27 90
22 90
32 70
32 90
22 70
Differences for alpha=0.05 (Tukey-Kramer Adjustment)
Not significant
Significant

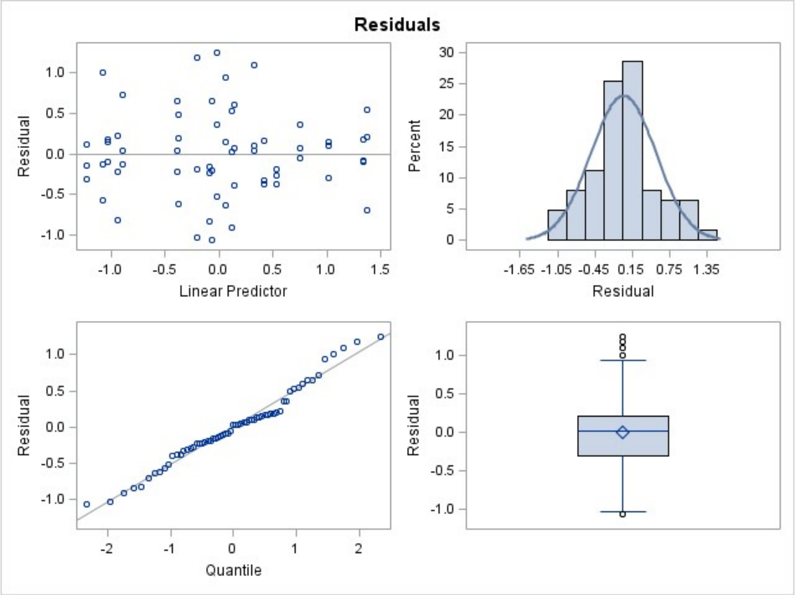
Residuals
Residual
Linear Predictor
Percent
Residual
Residual
Quantile
Residual

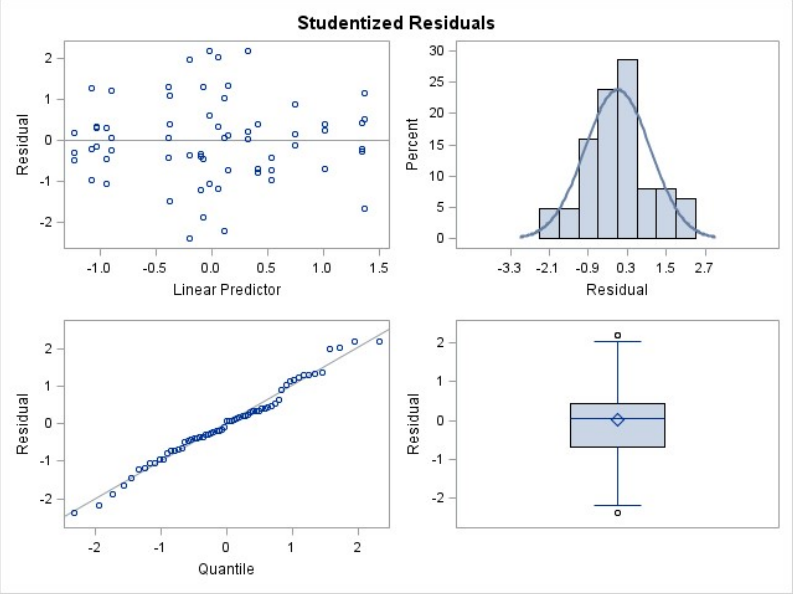
Studentized Residuals
Residual
Linear Predictor
Percent
Residual
Residual
Quantile
Residual

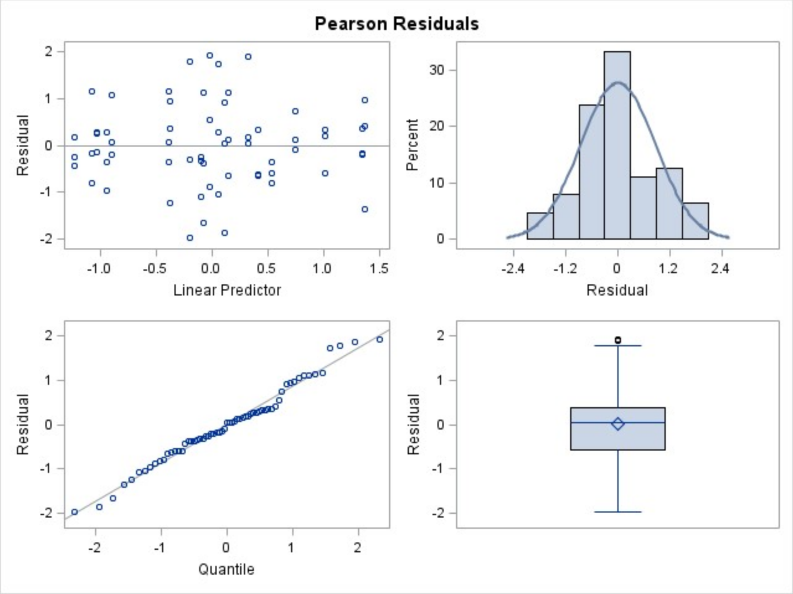
Pearson Residuals
Residual
Linear Predictor
Percent
Residual
Residual
Quantile
Residual

The SAS System

The GLIMMIX Procedure

Model Information	
Data Set	WORK.RAW
Response Variable (Events)	Adults
Response Variable (Trials)	Pupated
Response Distribution	Binomial
Link Function	Logit
Variance Function	Default
Variance Matrix	Diagonal
Estimation Technique	Maximum Likelihood
Degrees of Freedom Method	Residual

Class Level Information		
Class	Levels	Values
Species	3	SC SI SS
Temp	4	22 27 32 v
Humidity	3	70 90 v

Number of Observations Read	63
Number of Observations Used	63
Number of Events	1836
Number of Trials	3415

Dimensions	
Covariance Parameters	1
Columns in X	15
Columns in Z	0
Subjects (Blocks in V)	1
Max Obs per Subject	63

Optimization Information	
Optimization Technique	Newton-Raphson
Parameters in Optimization	7
Lower Boundaries	0
Upper Boundaries	0
Fixed Effects	Not Profiled

Iteration History					
Iteration	Restarts	Evaluations	Objective Function	Change	Max Gradient
0	0	4	253.39729325	.	1.941385
1	0	3	253.14170399	0.25558925	0.014434
2	0	3	253.14168595	0.00001805	1.787E-6

Convergence criterion (ABSGCONV=0.00001) satisfied.

Fit Statistics	
-2 Log Likelihood	506.28
AIC (smaller is better)	520.28
AICC (smaller is better)	522.32
BIC (smaller is better)	535.29
CAIC (smaller is better)	542.29
HQIC (smaller is better)	526.18
Pearson Chi-Square	236.67
Pearson Chi-Square / DF	4.23

Type III Tests of Fixed Effects

Effect	Num DF	Den DF	F Value	Pr > F
Temp	2	56	14.08	<.0001
Humidity	1	56	1.45	0.2331
Temp*Humidity	2	56	3.42	0.0398

Temp Least Squares Means

Temp	Estimate	Standard Error	DF	t Value	Pr > |t|	Mean	Standard Error Mean
22	Non-est	.	.	.	.	Non-est	.
27	Non-est	.	.	.	.	Non-est	.
32	Non-est	.	.	.	.	Non-est	.
v	Non-est	.	.	.	.	Non-est	.

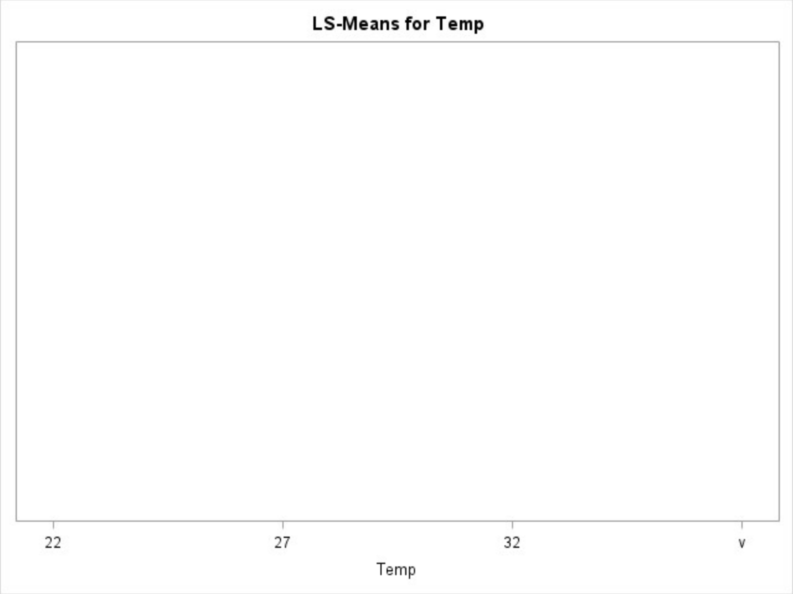

Differences of Temp Least Squares Means
Adjustment for Multiple Comparisons: Tukey

Temp	Temp	Estimate	Standard Error	DF	t Value	Pr > |t|	Adj P
22	27	-0.9144	0.2061	56	-4.44	<.0001	0.0001
22	32	-0.01959	0.2193	56	-0.09	0.9291	0.9956
22	v	Non-est	.	.	.	.	.
27	32	0.8948	0.1993	56	4.49	<.0001	0.0001
27	v	Non-est	.	.	.	.	.
32	v	Non-est	.	.	.	.	.

Temp*Humidity Least Squares Means

Temp	Humidity	Estimate	Standard Error	DF	t Value	Pr > |t|	Mean	Standard Error Mean
22	70	-0.9656	0.2532	56	-3.81	0.0003	0.2758	0.05057
22	90	-0.1199	0.1940	56	-0.62	0.5390	0.4701	0.04831
27	70	0.4653	0.1806	56	2.58	0.0126	0.6143	0.04279
27	90	0.2781	0.1885	56	1.48	0.1458	0.5691	0.04623
32	70	-0.5016	0.2113	56	-2.37	0.0211	0.3772	0.04963
32	90	-0.5447	0.2146	56	-2.54	0.0139	0.3671	0.04986
v	v	1.2432	0.1739	56	7.15	<.0001	0.7761	0.03022

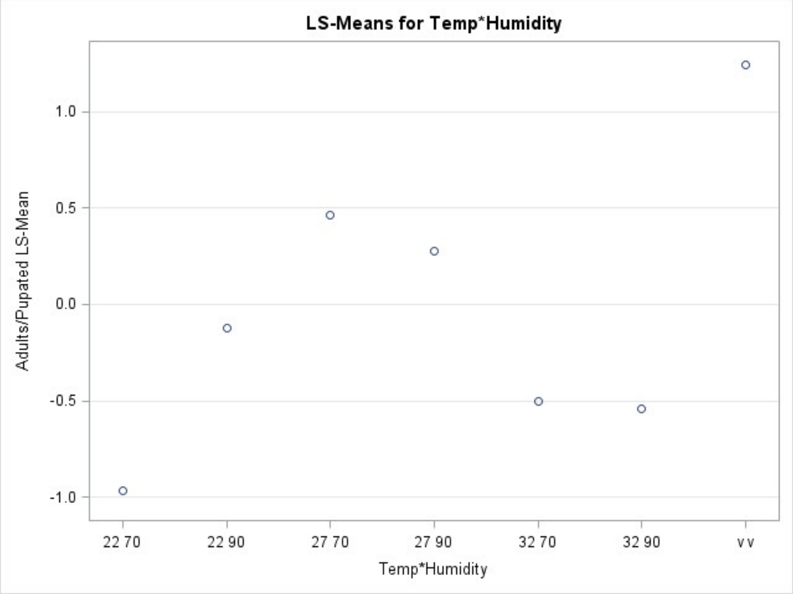

Differences of Temp*Humidity Least Squares Means
Adjustment for Multiple Comparisons: Tukey-Kramer

Temp	Humidity	Temp	Humidity	Estimate	Standard Error	DF	t Value	Pr > |t|	Adj P
22	70	22	90	-0.8457	0.3190	56	-2.65	0.0104	0.1305
22	70	27	70	-1.4309	0.3110	56	-4.60	<.0001	0.0005
22	70	27	90	-1.2437	0.3157	56	-3.94	0.0002	0.0041
22	70	32	70	-0.4640	0.3298	56	-1.41	0.1650	0.7958
22	70	32	90	-0.4209	0.3319	56	-1.27	0.2101	0.8634
22	70	v	v	-2.2088	0.3072	56	-7.19	<.0001	<.0001
22	90	27	70	-0.5851	0.2650	56	-2.21	0.0314	0.3079
22	90	27	90	-0.3979	0.2705	56	-1.47	0.1468	0.7602
22	90	32	70	0.3817	0.2868	56	1.33	0.1886	0.8345
22	90	32	90	0.4248	0.2893	56	1.47	0.1475	0.7617
22	90	v	v	-1.3631	0.2605	56	-5.23	<.0001	<.0001
27	70	27	90	0.1872	0.2610	56	0.72	0.4763	0.9910
27	70	32	70	0.9668	0.2779	56	3.48	0.0010	0.0161
27	70	32	90	1.0100	0.2805	56	3.60	0.0007	0.0113
27	70	v	v	-0.7779	0.2507	56	-3.10	0.0030	0.0446
27	90	32	70	0.7796	0.2832	56	2.75	0.0079	0.1040
27	90	32	90	0.8228	0.2856	56	2.88	0.0056	0.0774
27	90	v	v	-0.9651	0.2565	56	-3.76	0.0004	0.0070
32	70	32	90	0.04315	0.3012	56	0.14	0.8866	1.0000
32	70	v	v	-1.7448	0.2737	56	-6.38	<.0001	<.0001
32	90	v	v	-1.7879	0.2762	56	-6.47	<.0001	<.0001

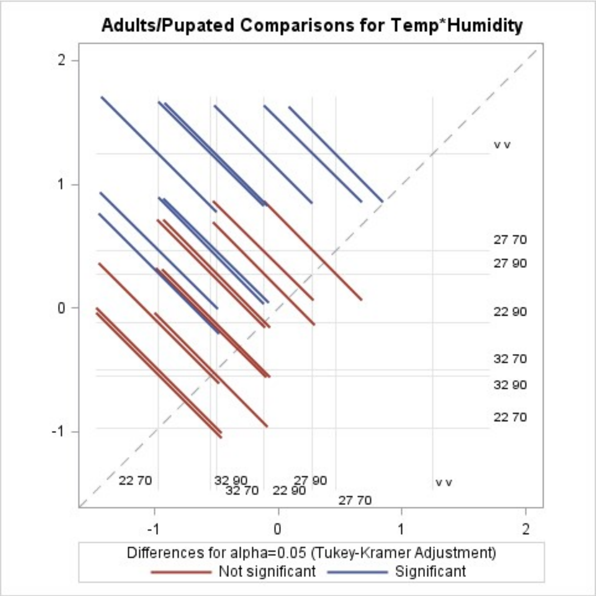
Adults/Pupated Comparisons for Temp*Humidity
v v
27 70
27 90
22 90
32 70
32 90
22 70
Differences for alpha=0.05 (Tukey-Kramer Adjustment)
Not significant
Significant

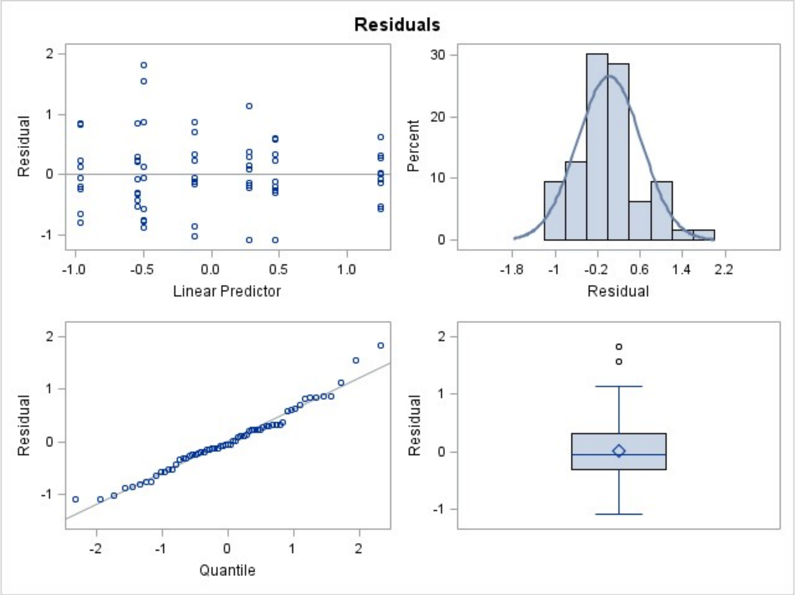
Residuals
Residual
Linear Predictor
Percent
Residual
Residual
Quantile
Residual

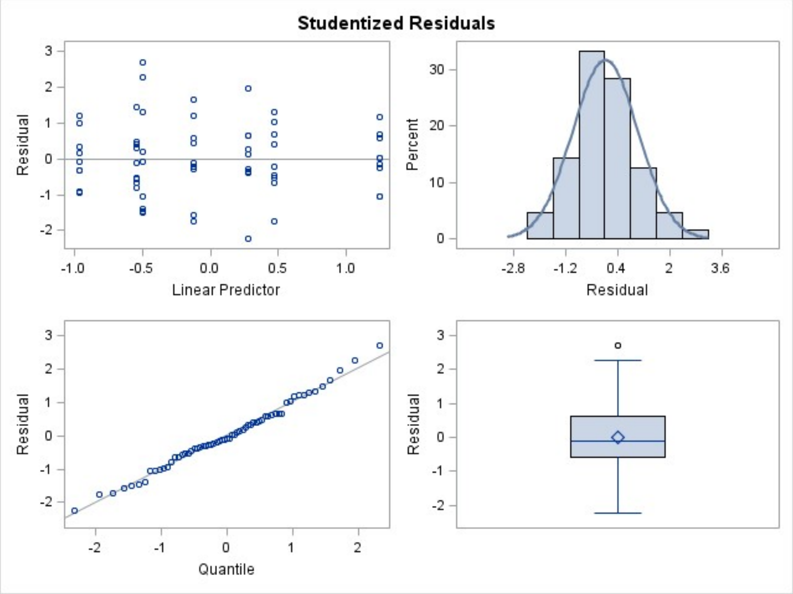
Studentized Residuals
Residual
Linear Predictor
Percent
Residual
Quantile

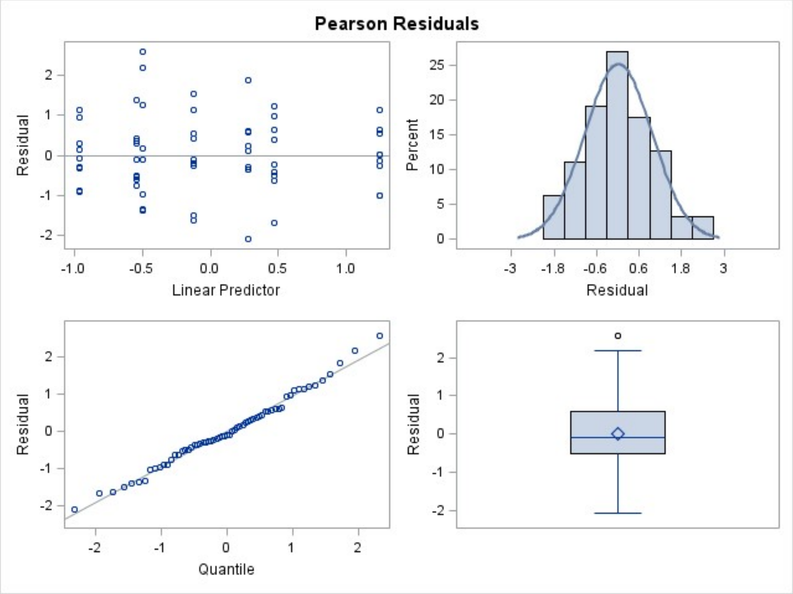
Pearson Residuals
Residual
Linear Predictor
Percent
Residual
Quantile

The SAS System

The GLIMMIX Procedure

Model Information	
Data Set	WORK.RAW
Response Variable (Events)	Adults
Response Variable (Trials)	Eggs
Response Distribution	Binomial
Link Function	Logit
Variance Function	Default
Variance Matrix	Diagonal
Estimation Technique	Maximum Likelihood
Degrees of Freedom Method	Residual

Class Level Information		
Class	Levels	Values
Species	3	SC SI SS
Temp	4	22 27 32 v
Humidity	3	70 90 v

Number of Observations Read	63
Number of Observations Used	63
Number of Events	1836
Number of Trials	9450

Dimensions	
Covariance Parameters	1
Columns in X	39
Columns in Z	0
Subjects (Blocks in V)	1
Max Obs per Subject	63

Optimization Information	
Optimization Technique	Newton-Raphson
Parameters in Optimization	17
Lower Boundaries	0
Upper Boundaries	0
Fixed Effects	Not Profiled

Iteration History

Iteration	Restarts	Evaluations	Objective Function	Change	Max Gradient
0	0	4	222.74754871	.	44.63651
1	0	3	221.55805276	1.18949595	0.809699
2	0	3	221.55738481	0.00066794	0.000496
3	0	3	221.55738481	0.00000000	3.51E-10

Convergence criterion (GCONV=1E-8) satisfied.

Fit Statistics	
-2 Log Likelihood	443.11
AIC (smaller is better)	477.11
AICC (smaller is better)	490.71
BIC (smaller is better)	513.55
CAIC (smaller is better)	530.55
HQIC (smaller is better)	491.44
Pearson Chi-Square	138.22
Pearson Chi-Square / DF	3.00

Type III Tests of Fixed Effects				
**Effect**	**Num DF**	**Den DF**	**F Value**	**Pr > F**
**Species**	2	46	1.24	0.2978
**Temp**	2	46	31.67	<.0001
**Humidity**	1	46	3.65	0.0624
**Species*Temp**	4	46	0.58	0.6766
**Species*Humidity**	2	46	2.59	0.0858
**Temp*Humidity**	2	46	9.34	0.0004

Temp Least Squares Means							
**Temp**	**Estimate**	**Standard Error**	**DF**	**t Value**	**Pr > |t|**	**Mean**	**Standard Error Mean**
22	Non-est	.	.	.	.	Non-est	.
27	Non-est	.	.	.	.	Non-est	.
32	Non-est	.	.	.	.	Non-est	.
v	Non-est	.	.	.	.	Non-est	.

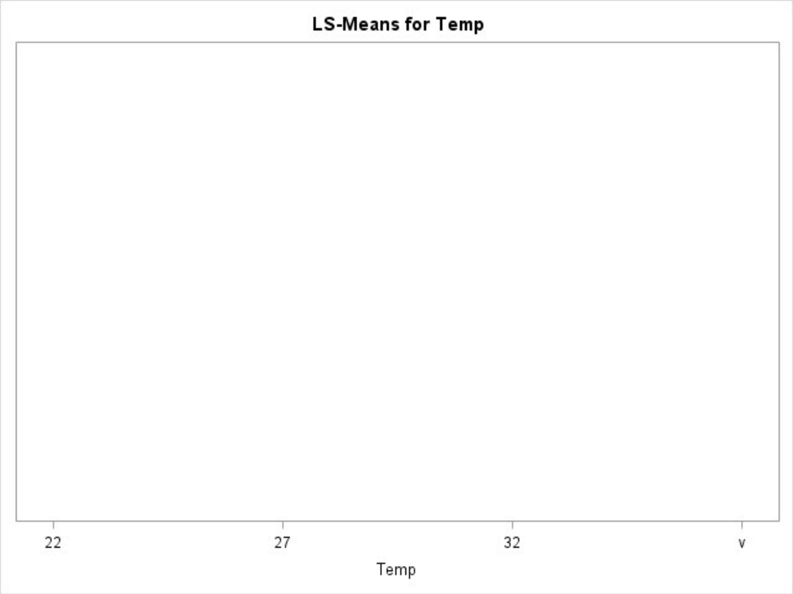

Differences of Temp Least Squares Means Adjustment for Multiple Comparisons: Tukey							
**Temp**	**Temp**	**Estimate**	**Standard Error**	**DF**	**t Value**	**Pr > |t|**	**Adj P**
22	27	-0.9194	0.1399	46	-6.57	<.0001	<.0001
22	32	-0.04563	0.1577	46	-0.29	0.7736	0.9549
22	v	Non-est	.	.	.	.	.
27	32	0.8738	0.1351	46	6.47	<.0001	<.0001
27	v	Non-est	.	.	.	.	.
32	v	Non-est	.	.	.	.	.

Temp*Humidity Least Squares Means								
**Temp**	**Humidity**	**Estimate**	**Standard Error**	**DF**	**t Value**	**Pr > |t|**	**Mean**	**Standard Error Mean**
22	70	-2.6368	0.1891	46	-13.95	<.0001	0.06681	0.01179
22	90	-1.6852	0.1301	46	-12.95	<.0001	0.1564	0.01717
27	70	-1.1188	0.1103	46	-10.14	<.0001	0.2462	0.02047
27	90	-1.3644	0.1174	46	-11.62	<.0001	0.2035	0.01904
32	70	-2.1011	0.1527	46	-13.76	<.0001	0.1090	0.01483
32	90	-2.1296	0.1535	46	-13.87	<.0001	0.1063	0.01458
v	v	-0.1525	0.09496	46	-1.61	0.1152	0.4620	0.02360

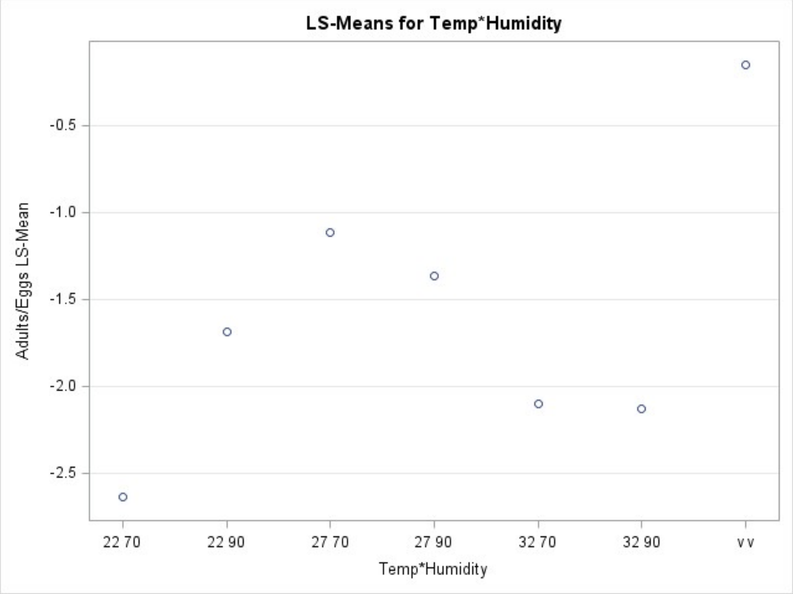

Differences of Temp*Humidity Least Squares Means
Adjustment for Multiple Comparisons: Tukey-Kramer

Temp	Humidity	Temp	Humidity	Estimate	Standard Error	DF	t Value	Pr > |t|	Adj P
22	70	22	90	-0.9515	0.2299	46	-4.14	0.0001	0.0026
22	70	27	70	-1.5180	0.2187	46	-6.94	<.0001	<.0001
22	70	27	90	-1.2724	0.2225	46	-5.72	<.0001	<.0001
22	70	32	70	-0.5356	0.2426	46	-2.21	0.0323	0.3115
22	70	32	90	-0.5072	0.2435	46	-2.08	0.0428	0.3796
22	70	v	v	-2.4843	0.2116	46	-11.74	<.0001	<.0001
22	90	27	70	-0.5665	0.1705	46	-3.32	0.0018	0.0271
22	90	27	90	-0.3208	0.1752	46	-1.83	0.0736	0.5350
22	90	32	70	0.4159	0.2005	46	2.07	0.0436	0.3844
22	90	32	90	0.4444	0.2012	46	2.21	0.0322	0.3112
22	90	v	v	-1.5328	0.1611	46	-9.52	<.0001	<.0001
27	70	27	90	0.2456	0.1611	46	1.52	0.1342	0.7288
27	70	32	70	0.9824	0.1879	46	5.23	<.0001	<.0001
27	70	32	90	1.0109	0.1892	46	5.34	<.0001	<.0001
27	70	v	v	-0.9663	0.1455	46	-6.64	<.0001	<.0001
27	90	32	70	0.7367	0.1927	46	3.82	0.0004	0.0067
27	90	32	90	0.7652	0.1931	46	3.96	0.0003	0.0045
27	90	v	v	-1.2119	0.1510	46	-8.02	<.0001	<.0001
32	70	32	90	0.02849	0.2158	46	0.13	0.8956	1.0000
32	70	v	v	-1.9487	0.1799	46	-10.83	<.0001	<.0001
32	90	v	v	-1.9771	0.1805	46	-10.95	<.0001	<.0001

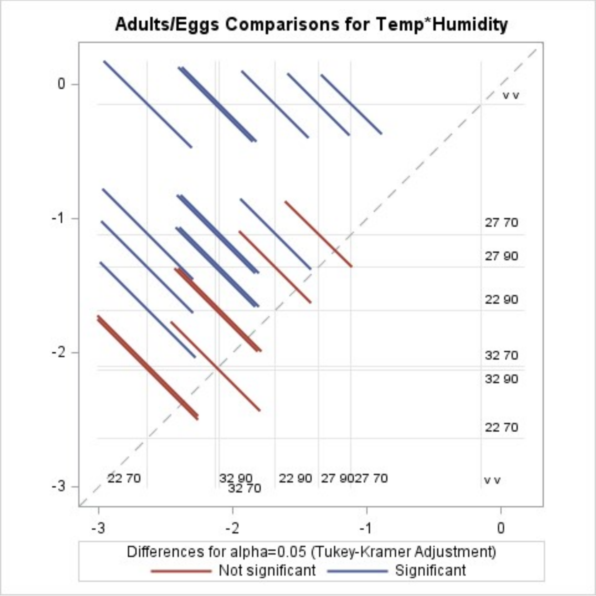
Adults/Eggs Comparisons for Temp*Humidity
27 70
27 90
22 90
32 70
32 90
22 70
22 70
32 90
32 70
22 90
27 90
27 70
Differences for alpha=0.05 (Tukey-Kramer Adjustment)
Not significant
Significant

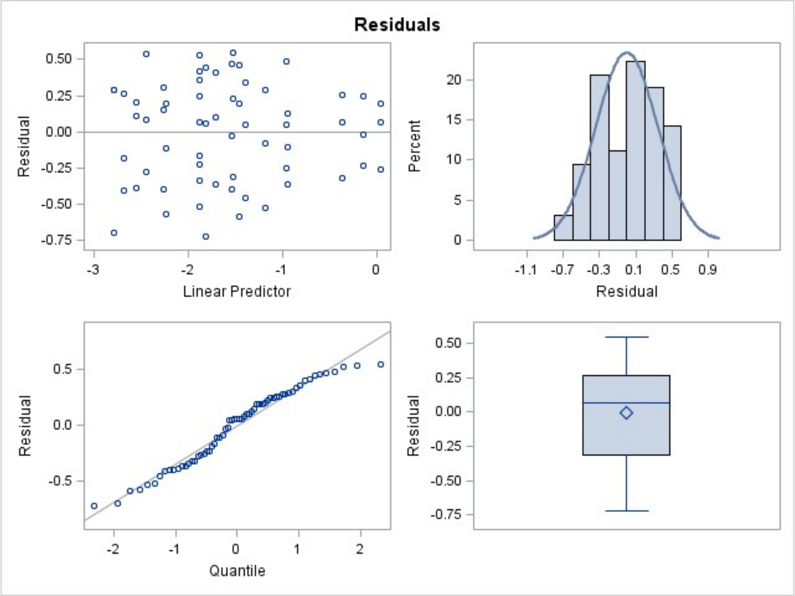
Residuals
Residual
Linear Predictor
Percent
Residual
Residual
Quantile
Residual

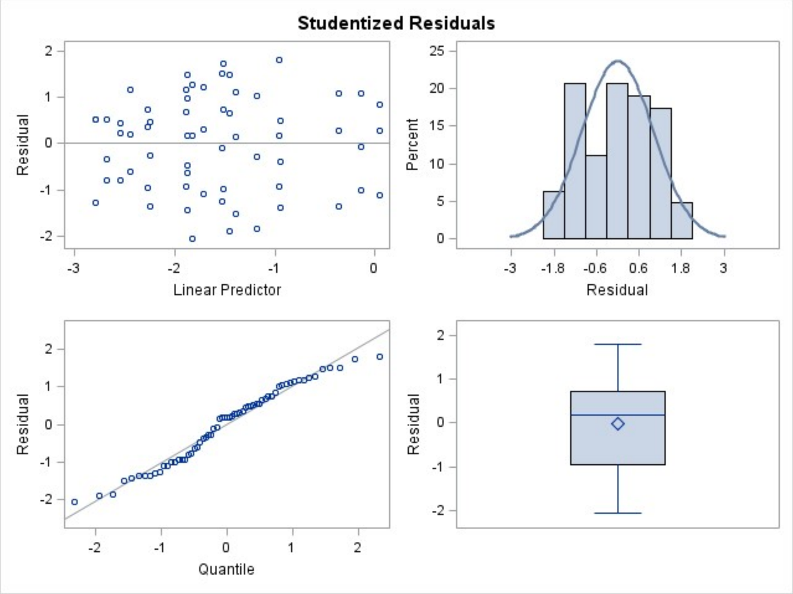
Studentized Residuals
Residual
Linear Predictor
Percent
Residual
Residual
Quantile
Residual

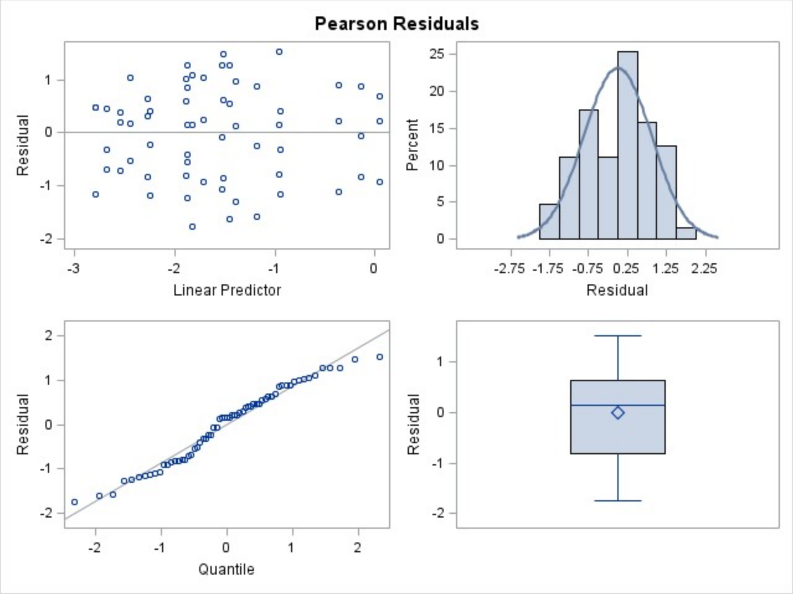
Pearson Residuals
Residual
Linear Predictor
Percent
Residual
Residual
Quantile
Residual

The SAS System

The GLIMMIX Procedure

Model Information	
Data Set	WORK.RAW
Response Variable (Events)	Adults
Response Variable (Trials)	Eggs
Response Distribution	Binomial
Link Function	Logit
Variance Function	Default
Variance Matrix	Diagonal
Estimation Technique	Maximum Likelihood
Degrees of Freedom Method	Residual

Class Level Information		
Class	Levels	Values
Species	3	SC SI SS
Temp	4	22 27 32 v
Humidity	3	70 90 v

Number of Observations Read	63
Number of Observations Used	63
Number of Events	1836
Number of Trials	9450

Dimensions	
Covariance Parameters	1
Columns in X	15
Columns in Z	0
Subjects (Blocks in V)	1
Max Obs per Subject	63

Optimization Information	
Optimization Technique	Newton-Raphson
Parameters in Optimization	7
Lower Boundaries	0
Upper Boundaries	0
Fixed Effects	Not Profiled

Iteration History					
Iteration	Restarts	Evaluations	Objective Function	Change	Max Gradient
0	0	4	246.55043556	.	58.64167
1	0	3	244.81814392	1.73229164	1.186998
2	0	3	244.81712875	0.00101517	0.000788
3	0	3	244.81712875	0.00000000	5.87E-10

Convergence criterion (GCONV=1E-8) satisfied.

Fit Statistics	
-2 Log Likelihood	489.63
AIC (smaller is better)	503.63
AICC (smaller is better)	505.67
BIC (smaller is better)	518.64
CAIC (smaller is better)	525.64
HQIC (smaller is better)	509.53
Pearson Chi-Square	183.46
Pearson Chi-Square / DF	3.28

Type III Tests of Fixed Effects				
**Effect**	**Num DF**	**Den DF**	**F Value**	**Pr > F**
**Temp**	2	56	29.10	<.0001
**Humidity**	1	56	3.04	0.0866
**Temp*Humidity**	2	56	8.78	0.0005

Temp Least Squares Means

Temp	Estimate	Standard Error	DF	t Value	Pr > |t|	Mean	Standard Error Mean
22	Non-est	.	.	.	.	Non-est	.
27	Non-est	.	.	.	.	Non-est	.
32	Non-est	.	.	.	.	Non-est	.
v	Non-est	.	.	.	.	Non-est	.

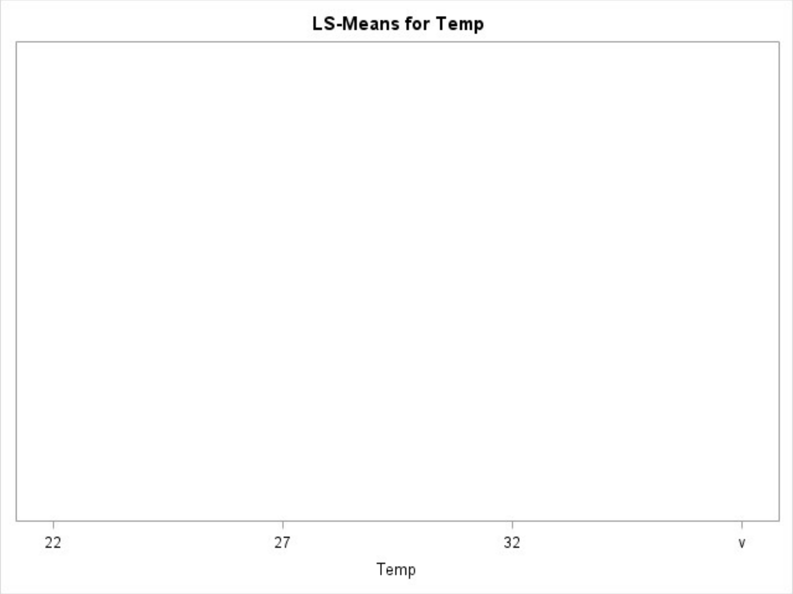

Differences of Temp Least Squares Means
Adjustment for Multiple Comparisons: Tukey

Temp	Temp	Estimate	Standard Error	DF	t Value	Pr > |t|	Adj P
22	27	-0.9222	0.1456	56	-6.33	<.0001	<.0001
22	32	-0.06281	0.1632	56	-0.38	0.7018	0.9217
22	v	Non-est	.	.	.	.	.
27	32	0.8594	0.1392	56	6.17	<.0001	<.0001
27	v	Non-est	.	.	.	.	.
32	v	Non-est	.	.	.	.	.

Temp*Humidity Least Squares Means

Temp	Humidity	Estimate	Standard Error	DF	t Value	Pr > |t|	Mean	Standard Error Mean
22	70	-2.6272	0.1965	56	-13.37	<.0001	0.06741	0.01235
22	90	-1.6804	0.1354	56	-12.41	<.0001	0.1570	0.01792
27	70	-1.1045	0.1139	56	-9.69	<.0001	0.2489	0.02130
27	90	-1.3587	0.1221	56	-11.12	<.0001	0.2044	0.01987
32	70	-2.0645	0.1558	56	-13.25	<.0001	0.1126	0.01557
32	90	-2.1175	0.1591	56	-13.31	<.0001	0.1074	0.01525
v	v	-0.1514	0.09881	56	-1.53	0.1311	0.4622	0.02456

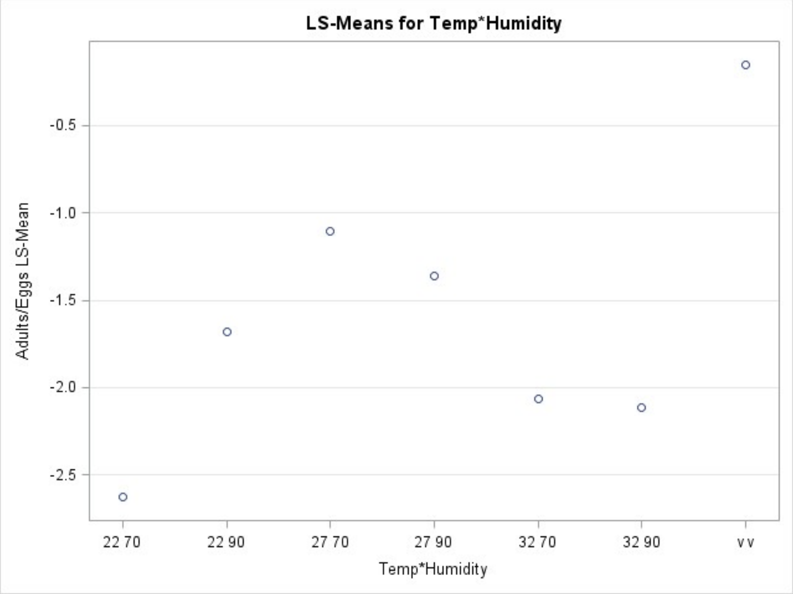

Differences of Temp*Humidity Least Squares Means
Adjustment for Multiple Comparisons: Tukey-Kramer

Temp	Humidity	Temp	Humidity	Estimate	Standard Error	DF	t Value	Pr > |t|	Adj P
22	70	22	90	-0.9468	0.2386	56	-3.97	0.0002	0.0037
22	70	27	70	-1.5227	0.2271	56	-6.70	<.0001	<.0001
22	70	27	90	-1.2685	0.2314	56	-5.48	<.0001	<.0001
22	70	32	70	-0.5627	0.2508	56	-2.24	0.0288	0.2896
22	70	32	90	-0.5097	0.2528	56	-2.02	0.0486	0.4164
22	70	v	v	-2.4758	0.2199	56	-11.26	<.0001	<.0001
22	90	27	70	-0.5759	0.1770	56	-3.25	0.0019	0.0299
22	90	27	90	-0.3217	0.1824	56	-1.76	0.0832	0.5771
22	90	32	70	0.3841	0.2064	56	1.86	0.0681	0.5143
22	90	32	90	0.4371	0.2089	56	2.09	0.0410	0.3715
22	90	v	v	-1.5290	0.1676	56	-9.12	<.0001	<.0001
27	70	27	90	0.2542	0.1670	56	1.52	0.1337	0.7307
27	70	32	70	0.9600	0.1931	56	4.97	<.0001	0.0001
27	70	32	90	1.0130	0.1957	56	5.18	<.0001	<.0001
27	70	v	v	-0.9531	0.1508	56	-6.32	<.0001	<.0001
27	90	32	70	0.7058	0.1980	56	3.56	0.0008	0.0126
27	90	32	90	0.7588	0.2006	56	3.78	0.0004	0.0066
27	90	v	v	-1.2073	0.1571	56	-7.68	<.0001	<.0001
32	70	32	90	0.05297	0.2227	56	0.24	0.8129	1.0000
32	70	v	v	-1.9131	0.1845	56	-10.37	<.0001	<.0001
32	90	v	v	-1.9661	0.1873	56	-10.50	<.0001	<.0001

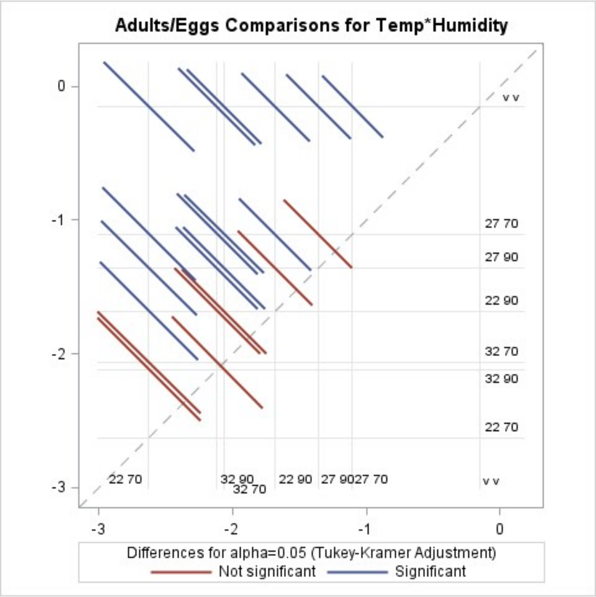
Adults/Eggs Comparisons for Temp*Humidity
0
-1
-2
-3
v v
27 70
27 90
22 90
32 70
32 90
22 70
22 70
32 90
32 70
22 90
27 90
27 70
v v
-3
-2
-1
0
Differences for alpha=0.05 (Tukey-Kramer Adjustment)
Not significant
Significant

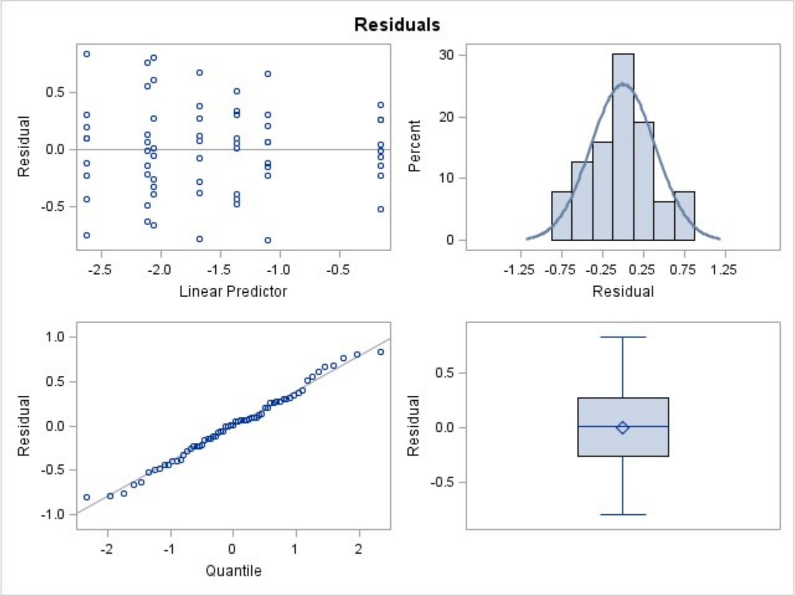
Residuals
Residual
Linear Predictor
-2.5
-2.0
-1.5
-1.0
-0.5
0.5
0.0
-0.5
Percent
30
20
10
0
-1.25
-0.75
-0.25
0.25
0.75
1.25
Residual
1.0
0.5
0.0
-0.5
-1.0
-2
-1
0
1
2
Quantile
Residual
0.5
0.0
-0.5

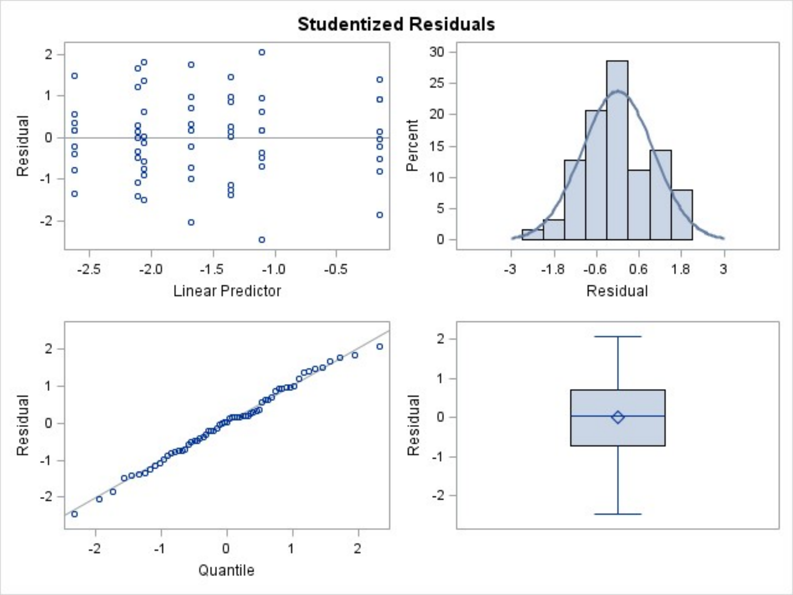
Studentized Residuals
Residual
Linear Predictor
Percent
Residual
Quantile

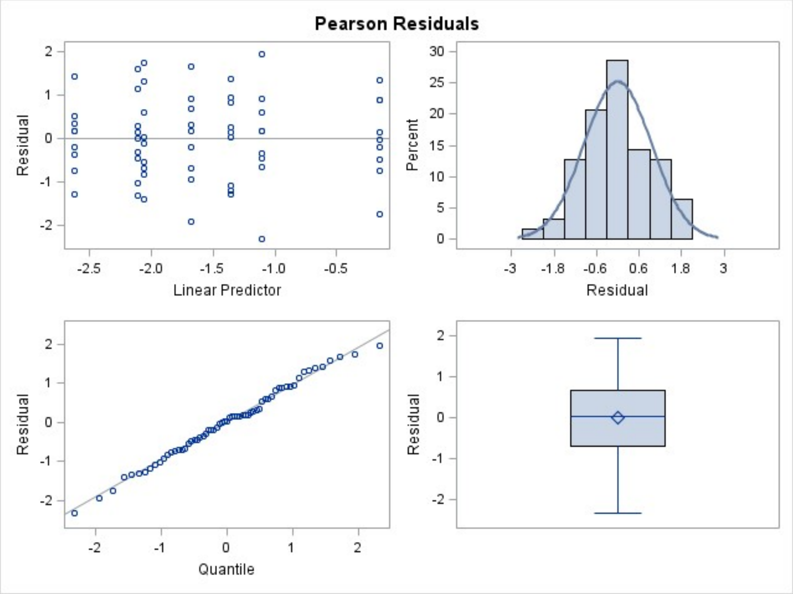
Pearson Residuals
Residual
Linear Predictor
Percent
Residual
Quantile
