# Supplementary material for: The combined effects of temperature and relative humidity parameters on the reproduction of Stomoxys species in a laboratory setting
Source: PLoS One. 2020 Dec 21;15(12):e0242794. doi: 10.1371/journal.pone.0242794 (PMC7751869; doi:10.1371/journal.pone.0242794)
Supplement: Attachment — Submitted filename: Issimov et al The combined effects of temperature and relative humidity parameters on the reproducti.docx [file pone.0242794.s003.docx]

**The combined effects of temperature and relative humidity parameters on the reproduction of Stomoxys species in a laboratory setting**

***Arman Issimov*^1^, Kuandyk Zhugunissov^2^, Kamshat Shorayeva^2^, Lespek Kutumbetov^2^, Marzhan Djumagazieva^3^, Assylbek Zhanabayev^4^, Birzhan Nurgaliyev^5^, Malik Shalmenov^5^, Gaisa Absatirov^5^, Laura Dushayeva^5^, Peter J. White^1^.***

1. Sydney School of Veterinary Science, Faculty of Science, University of Sydney
2. RGE “Research Institute for Biological Safety Problems” Committee of Science, The Ministry of Education and Science of the Republic of Kazakhstan
3. Consultative and Diagnostic Department, Medical Center LLP, Uralsk, Kazakhstan
4. Department of Veterinary Medicine, Saken Seifullin Kazakh Agrotechnical University
5. Department of Veterinary Medicine, Zhangir Khan West Kazakhstan Agrarian – Technical University

**** Corresponding Author***

**Abstract**

In this study, *Stomoxys* species (S. calcitrans, S. sitiens and S. indica) were examined to improve on the current technique for mass rearing using a method of combined incubation parameters. Moreover, the reproductive potential of immature forms at various stages of development was defined.

Immature forms of stable flies were incubated according to species. There was no significant difference in the number of immature forms obtained among species incubated under the same conditions. Six incubation parameters were used in combination, at temperatures (T) of 32 ℃, 27 ℃ and 22 ℃ and relative humidity (RH) of 90% and 70% RH. The combined method resulted in a high number of eggs hatching at 32 ℃ and 90% humidity as well as an increase in the number of larva pupated and emergence of imago at 27 ℃ and 70% humidity.

**Keywords:** Stomoxys spp; humidity; temperature; vector born disease; mechanical vector; combined method.

**Introduction**

Stable flies (*Stomoxys* spp) have long been considered a major pest of livestock in Kazakhstan and are capable of transmitting pathogens present in infected animals. (1). A high concentration of these flies is observed in almost all regions in Kazakhstan with adult *Stomoxys* flies emerging in huge numbers following rainfall events in the later months of spring. Farms are a preferred habitat for stable flies where they can be found in large numbers in association with livestock (2, 3). They interrupt grazing of cattle and cause painful bites, which in turn leads to weight loss in adult and yearling cattle as well as reduced milk yield (4-6). As a result, farmers have developed strategies to minimize direct contact between animals and biting flies, through pasturing animals in the early morning and in the evening while keeping them in enclosed barns during the daytime when the biting insects numbers are highest.

Stable flies are mechanical vectors for many infectious pathogens (7(8-11)). In previous work, we defined the role of *Stomoxys* species in the transmission of Lumpy Skin Disease Virus (LSDV) (12). To do so, a constant supply of large numbers of *Stomoxys* flies reared in laboratory conditions was required. Attempts to produce flies using previously published methods (13, 14) were unsuccessful in obtaining a high yield and as result the aim of this study was to improve the reproduction of three species of *Stomoxys* using various ambient temperature and relative humidity settings on the different developmental stages of flies.

**Materials and methods**

***Fly catching***

Two local farms in the vicinity of the Research Institute for Biological Safety Problems (RIBSP) in Zhambyl oblast, Kazakhstan practising mixed dairy and beef management were examined for the presence of a population of stable fly. Three *Stomoxys* species were identified, namely *S. calcitrans, S. sitiens* and *S. indica,* with *S. sitiens* considered dominant over the other species.

A fly-catching unit (pyramid) consisting of a cellophane-coated three-piece pyramid-shaped structure was used to collect flies (Fig. 1a). The width of the construction at its base was 10 m and 12 m height. At the tip of the pyramid, there was a hole of 10 cm in diameter, on which a container was installed to collect flies that enter it as they fly up along the inside of the pyramid. The unit was installed directly onto a dung heap so that the distance between the lower part of the pyramid and the dung was 5-7 cm. Similarly, several units were placed along haystacks. In addition to the unit described above, a commercial fly catching unit “Miniature CDC light trap with UV light” (USA) (Fig. 1b) was used. The light trap was suspended from the ceiling of cattle barns. The fly traps were monitored every two hours for the presence of *Stomoxys spp*. Each batch of newly caught *Stomoxys spp* was transported immediately to the fly rearing compartment of the Entomology section of the RIBSP for further experimentation. Approximately 780 field collected flies were kept for rearing in glass cages.

**Figure 1.** **a** – fly-catching unit (pyramid). **b** – Miniature CDC light trap with UV light.

***Morphological identification***

Morphological identification was carried out using Xper^2^ software (LIS, France) and the protocol based on Zumpt (15). Stable flies were anaesthetized using CO2 in a gas chamber and placed onto the light board with a Bi-Aspheric magnification lens (COIL 4212 Fixed Stand Magnifier - 12X). Morphological characteristics of *Stomoxys* spp, for example, venation of wings, dorso-abdominal feature, legs and thoracic pattern were defined utilizing a stereomicroscope as well as a table with the morphological features for each species (16) . *Stomoxys spp* were then sorted according to species and allowed to recover. The mean recovery time was established at 20 min post-anaesthesia. The mortality rate due to CO2 exposure constituted up to 1 % per batch. Each batch contained 60 flies of mixed *Stomoxys spp.*

***Fly rearing***

*Preparation of cages for oviposition*

A method of rearing stable flies described by Parr (1959) was modified to obtain sufficient egg production and increased larval survival. The main reason for the modification was the extremely low hatching and larvae survival rates during the first instar obtained from the previous method.

The walls of the quadrangular cages for oviposition measured 45cm x 45cm x 1000cm and were built from wooden sheets (floor, back and sidewalls) and transparent plastic (Plexiglas) (top and front walls). This construction provided excellent illumination that directly affected the activity of the *Stomoxys* flies. On the top of the cage there were four holes cut 4 cm in diameter each fitted with removable mosquito net. They were located 20 cm from each other to allow air circulation and these holes were used for feeding and/or loading newly emerged or field caught adult flies. In the center at the bottom of the front wall, a sliding door, 20 cm in width and 5 cm in height, was made to enable personnel to clean and to introduce oviposition medium in the cage.

*Maintenance of adults and egg collection*

Cages containing field caught *Stomoxys* flies were placed in the vivarium of the entomology section of the RIBSP and maintained at 24℃ and 85% humidity. Fresh citrated bovine blood soaked into cotton pads was provided twice daily at 9 a.m. and at 5 p.m.

Based on the protocol published by Parr (13) a modified medium was designed containing 400g fresh dung and 200g clotted bovine blood. The fresh manure was collected from the insect secured animal facility of RIBSP to eliminate any contamination by other insects. Blood was collected in glass bottles from the abattoir during slaughter and then allowed to clot for 30 min. These components were mixed (Viscosity stirrer AISI 316 steel, Bochem, Germany) and incubated at room temperature overnight. The mixture obtained was equally divided into several shallow plastic pans 15 cm in diameter and 3 cm in height. The pans were then placed into the cages. Gravid female *Stomoxys* flies readily laid eggs in the crevices of the medium. Eggs were also found to a depth of 0.5 cm beneath the surface. The pans were removed twice a day and placed into the incubation chamber for further development.

*Effects of temperature and relative humidity on the development stages of the immature forms of Stomoxys species.*

Experiments were conducted to determine the ideal temperature and humidity range during the incubation to obtain a high degree of hatching. In addition to this, the effect of these factors on the viability of larva and pupae stages as well as on imago emergence was investigated. Gravid female flies were placed in the cylindrical vessels and allowed to oviposit. Eggs collected were divided into batches. Several batches each containing 150 eggs of each *Stomoxys* species were placed into circular pans 15 cm in diameter and 3 cm in depth. In each pan, 300 g medium was added and incubated separately using different incubation settings, namely at temperatures of 32℃, 27℃, 22℃ and relative humidity (RH) of either 90% (experiment 1) or 70% (experiment 2). The experiments were run in triplicates and mean values calculated.

The larval medium was placed into incubation chamber 6 hours prior to experiment commencement and the temperature of the substrate was measured hourly using Type K Thermocouples (Australia) to avoid any fluctuations in the incubation parameters. The medium was monitored every 4 hours for presence any changes in the development of immature *Stomoxys* flies as well as to calculate mean periods of incubation.

In preliminary studies, significant effects of some incubation conditions on the viability and yield of certain stages of immature *Stomoxys* flies were detected. This led to a consideration of utilizing various incubation parameters within a single life cycle of *Stomoxys* flies. To do so, experiment 3 was carried out using a combination of environmental settings, which had demonstrated potential significance on the development of immature stable flies. The combination method was performed as follows: egg maturation was conducted at 32℃, 90% RH; incubation from larva until pupation completion at 27℃, 70% RH; from pupae to imago eclosion at27℃, 70% RH. The content of the medium used in experiment 3 was similar to that utilized in experiments 1 and 2.

The mean time period required for completion of each developmental stage was not extended or reduced specifically to obtain a high yield of immature flies and was determined according to Friesen, Berkebile (14). The experiment was run in triplicate and mean values were calculated.

**Data analysis**

Statistical analysis was conducted using two-way ANOVA and Tukey’s multiple comparisons test in Graphpad Prism 6.0 (Graphpad Software Inc., CA, USA) to define mean values and significant differences between developmental stages using different environmental settings. A value of p≤0.05 was designated statistically significant. The data obtained during this study are openly available in figshare at <https://doi.org/10.6084/m9.figshare.12212549.v1>.

**Results**

***Eggs***

There were no significant differences in the number of eggs hatched among species within each incubation condition (*p*= 0.091). As illustrated in Table 1, eggs maintained at 90% RH demonstrated the largest number of hatching at a temperature of 32 °C. A significant difference was found between groups incubated at 32 ° C, 90% RH and 27, 22°C, 90% RH; 32, 27, 22°C 70% RH (*P* ≤ 0.0001; 95% CI), whereas there was no difference detected between groups maintained at 27, 22°C, 90% RH and 32, 27, 22°C, 70% RH *p* > 0.05 (Figure 2). ANOVA revealed there was no significant difference in the duration of hatching period between groups (*p*= 0.098).

**Table 1.** Summary of incubation parameters affecting on immature forms of *Stomoxys* species.

| Species | t^о^С | Stage of development | | | | | | | | |
| --- | --- | --- | --- | --- | --- | --- | --- | --- | --- | --- |
|  |  | Eggs* | | | Larvae** | | | Pupae*** | | |
|  |  | Period, hours/mean | Number of hatched/ mean±SD | % hatched | Period, hours/ mean | Number of pupated/ mean±SD | % pupated | Period, hours/ mean | Number of imago emerged/ mean±SD | % emerged |
| **Relative humidity 90%** | | | | | | | | | | |
| SC | 32^о^С^Abc^ | 25 (12-30) | 119±8.50 | 79.3 | 240  (235-250) | 49±9.01 | 33 | 144 (от 140 до 150) | 22±6.80 | 17 |
| SS |  | 28 (13-32) | 126±9.45 | 82 | 240  (220-255) | 32±6.55 | 21 | 120 (96-144) | 12±5.03 | 8 |
| SI |  | 22 (15-27) | 133±6.02 | 89 | 264  (245-276) | 51± 12.8 | 34 | 120 (100-138) | 14±4.72 | 9 |
|  | | | | | | | | | | |
| SC | 27^о^С^a^ | 27 (12-31) | 96±13.2 | 64 | 216 (204-228) | 57±14.0 | 38 | 48 (40-72) | 32±11.0 | 21 |
| SS |  | 24 (18-29) | 72±11.9 | 48 | 216 (200-228) | 44±9.60 | 29 | 72 (50-84) | 30±9.60 | 20 |
| SI |  | 24 (10-29) | 87±9.71 | 58 | 240 (216-260) | 60±12.2 | 40 | 48 (40-56) | 29±9.60 | 19 |
|  | | | | | | | | | | |
| SC | 22^о^С^a^ | 30 (18-35) | 99±8.50 | 66 | 312 (264-327) | 59±3.51 | 39 | 52 (45-60) | 24±7.55 | 16 |
| SS |  | 26 (19-32) | 71±9.01 | 60,6 | 288 (238-306) | 44±7.02 | 29 | 72 (60-85) | 30±8.02 | 20 |
| SI |  | 27 (16-33) | 94±9.01 | 47 | 288 (230-312) | 47±6.50 | 31 | 72 (60-84) | 27±9.53 | 18 |
| **Relative humidity 70%** | | | | | | | | | | |
| SC | 32^о^С^ab^ | 30 (21-33) | 102±10.5 | 68 | 240 (216-264) | 44±10.6 | 29 | 96 (90-102) | 18±7.55 | 12 |
| SS |  | 26 (19-29) | 89±12.7 | 59 | 240 (200-252) | 39±4.61 | 26 | 96 (86-106) | 22±6.55 | 14 |
| SI |  | 25 (16-30) | 93±15.7 | 62 | 240 (228-250) | 51±9.61 | 34 | 96 (90-102) | 11±3.21 | 7.3 |
|  | | | | | | | | | | |
| SC | 27^о^С^aBC^ | 24 (15-29) | 84±7.67 | 56 | 240 (210-256) | 62±11.5 | 41 | 72 (65-80) | 44±11.1 | 29 |
| SS |  | 24 (17-26) | 93±10.1 | 62 | 240 (220-246) | 69±14.2 | 46 | 96 (84-108) | 39±7.51 | 26 |
| SI |  | 24 (15-28) | 75±9.45 | 50 | 216 (202-228) | 51±9.01 | 34 | 72 (66-78) | 42±11.3 | 28 |
|  | | | | | | | | | | |
| SC | 22^о^С^abc^ | 26 (16-29) | 101±9.07 | 67 | 288 (240-312) | 34±7.02 | 22 | 72 (67-96) | 9±3.05 | 6 |
| SS |  | 34 (20-38) | 96±5.56 | 64 | 288 (246-300) | 40±9.53 | 26 | 72 (62-96) | 13±4.51 | 8.6 |
| SI |  | 31 (17-37) | 112±11.1 | 75 | 264 (236-294) | 36±10.6 | 24 | 72 (65-96) | 7±4.04 | 4,6 |

Digits in parentheses represents the mean onset and completion time of hatching, pupation and eclosion periods in triplicates.

(*) – A total number of eggs used in the experiment was 150eggs for each *Stomoxys* species.

(**) - The number of larva pupated from first instar hatched.

(***)The number of imago emerged from viable pupae.

A vs a – significant difference between incubation parameters is *p≤0.0001*

B vs b – significant difference between incubation parameters is from *p≤0.001 to p≤0.01*

B vs b – significant difference between incubation parameters is *p≤0.0001*

SC - *Stomoxys calcitrans*

SS - *Stomoxys sitiens*

SI - *Stomoxys indica*

**Figure 2.** Statistical differences between different groups of *Stomoxys* flies incubated under various environmental conditions. **⃰** - significant, ns – not significant; RH – Relative Humidity; t – temperature; **(a)**The mean number of eggs hatched out of total 150eggs for each species incubated under 32℃, 27℃, 22℃/ 90% RH and 32℃, 27℃, 22℃/ 70 RH. **(b)**The mean number of larva pupated out of total 150eggs for each species incubated under 32℃, 27℃, 22℃/ 90% RH and 32℃, 27℃, 22℃/ 70 RH. **(c)**The mean number of imago emerged out of total 150 eggs for each species incubated under 32℃, 27℃, 22℃/ 90% RH and 32℃, 27℃, 22℃/ 70 RH.

***Larva***

As shown in Table 1, the optimal conditions for raising *Stomoxyx* larva was found to be 27°C, 90% RH. The duration of the larval period was not significantly different between groups maintained under 32, 27, 22°C 70% RH and 32, 27, 22°C 90% RH (*P* = 0.074, p≥0.05). There was a difference in the pupa formation between groups incubated at 27 °C, 70% RH and 32°C, 70% RH (*P* ≤ 0.002; 95% CI) (Figure 2). The number of larva pupated was also significantly different for the 27 °C, 90% RH and 22 ° C, 70% RH groups (*P* ≤ 0.001; 95% CI) (Figure 2). There was a significant difference in the larva maturation between groups reared under 32°C, 90% RH and 27 °C, 70% RH (*P* ≤ 0.01; 95% *CI*). The relationship was not significant between groups (p≥0.05) reared under 27 °C, 70% RH and 27°C, 22°C, 90% RH accounting for *P* = 0.655 and *P* = 0.215 respectively.

***Pupa***

The emergence of adult flies was recorded significantly higher under 27°C, 70% RH (*P* ≤ 0.0001; 95 % *CI*) for SC, SS, and SI respectively. No significant difference was detected in the number of *Stomoxys* flies that emerged under 32°C, 27°C, 22°C, 90% RH and 22°C, 32°C, 70% RH (*P* ≥ 0.05) (Figure 2). As demonstrated in Table 1, the eclosion of imago maintained at 27 °C, 70% RH shows the highest number of emergence compare to those incubated at 27 and 22 °C.

**Combined method**

The survival percentage for a total number of 150 eggs incubated under 32 °C, 90% RH was above 80 % whereas the yield percentage for larvae and pupae maintained under 27 °C, 70% RH varied slightly averaging 55% and 48% respectively. The mean quantitative indicators obtained from experiment 3 are shown in Table 2. In addition, linear comparison was demonstrated in Figure 3.

**Table 2.** Quantitative indicators of the development of immature flies using combined incubation parameters in a single life cycle.

| Species | Stage of development | | | | | | | | |
| --- | --- | --- | --- | --- | --- | --- | --- | --- | --- |
|  | Eggs (32^о^С, 90% RH) | | | Larvae (27^о^С, 70% RH) | | | Pupae (27^о^С, 70% RH) | | |
|  | Period, hours/mean | Number of hatched/mean | % hatched | Period, hours/ mean | Number of pupated/mean | % pupated | Period, hours/ mean | Number of imago emerged/mean | % emerged |
| SC | 29 (15-33) | 115±15.04 | 76.6 | 240 (204-268) | 84±9.53 | 56 | 144 (120-156) | 62±10.7 | 41 |
| SS | 27 (17-30) | 131±9.45 | 87.3 | 240 (220-262) | 97±11.01 | 49 | 132 (108-156) | 77±8.73 | 51 |
| SI | 23 (15-28) | 120±7.02 | 80 | 240 (204-280) | 87±8.62 | 58 | 120 (108-140) | 70±9.01 | 53 |

SC - *Stomoxys calcitrans*

SS - *Stomoxys sitiens*

SI - *Stomoxys indica*

**Figure 3.** Linear comparison of survival rates of immature *Stomoxys* species incubated under various fixed conditions vs combined method. The combined method was carried out as follows: egg maturation was conducted at 32℃, 90% RH; incubation from larva until pupation completion at 27℃, 70% RH; from pupae to imago eclosion at 27℃, 70% RH. E – eggs; L – larvae; P – pupae.

**Discussion**

Blood feeding insects have long been considered as carriers of infectious diseases of animals (17-20), including lumpy skin disease virus of cattle (12, 21, 22). Such insects include flies of *Stomoxys* species. To study the role of these flies inhabiting the territory of the Republic of Kazakhstan in the epizootiology of LSD, we have shown that three species of *Stomoxys* flies are capable of transmitting LSDV from diseased to healthy animals using a fractional feeding method (12). In connection to this study, the work presented here aimed to study the biology of reproduction of these flies and, based on the results of which, modelling the microclimate for their maintenance and reproduction under laboratory conditions. The created model will subsequently be used in studies evaluating target flies in the role of the mechanical and biological vector of the causative agent of LSD and other vector-borne diseases during spread within the herd and reservation in the natural niche in the interepizootic period.

As the study results show, the developmental biology of all three species of *Stomoxys* flies was found to be similar. Immature forms of all three species (SC, SS, SI) at the stages of eggs, larva, pupa and imago under the same microclimate conditions developed satisfactorily and similarly (23). In this study, most of the results obtained align with previous results on the effects of ambient environment on the species’ survival and developmental time under laboratory condition (24, 25). As for the tested microclimate parameters, we selected two levels of relative humidity, which were maintained at 70% or 90%, and the ambient temperature was selected as equal to 22 ° C, 27 ° C, or 32 ° C. The selection of these parameters was based on studies reported by Kunz, Berry (24) and McPheron and Broce (26). Observation of eggs laid and maintained under the indicated microclimate conditions showed that the duration of the hatching period at various temperatures and different humidity were approximately similar to each other, except for a temperature of 22 °C, at which this time was extended by 2.6 hours at 90 % humidity and by 3.3 - 6.3 hours at 70% humidity compared with other temperature settings. This observation is in agreement with findings reported by Lysyk (23).

At both humidity indices, the predominantly favourable parameter for the formation of pupae was observed when incubated at 27 °C, at which the maximum number of pupae were obtained (35.6% of pupae at 90% humidity and 40.3% of pupae at 70% humidity). The results obtained demonstrate that pupation most favorably occurs at incubation under 27 °C and 70% relative humidity. This, in turn, is consistent with previous studies where larvae selected moderate moisture levels in which to pupate avoiding excessively high and low moisture levels (26, 27).

The data obtained during the observation of the final biological development stage of flies demonstrates that humidity and ambient temperature play a decisive role in the active formation and emerging of adult insects. The most suitable indicators of these parameters were humidity equal to 70% and temperature of 27 ° C.

Analysis of the study results is indicated that for the active development of flies and their reproduction in high numbers, a certain microclimate is required (28-30). At the stage of eggs, an increased humidity of 90% and a relatively high temperature reaching 32 °C is favorable and productive for their transition into a larval stage. In this mode, up to 80% of larvae can be obtained from the initial number of eggs laid by an adult fly. Probably, the high humidity level is essential for the formation of larvae. Therefore, they burrow deeper into the medium, where the humidity is higher, from the level at which the eggs were laid.

At the next stage of biological development, when pupation occurs, the demand for higher humidity decreases. A possible explanation for this could be a lower moisture requirement to form a puparium as well as the number of formed pupae becomes relatively small. Therefore, a decrease in humidity at this stage of development stimulates the formation of an increased number of pupae, particularly when incubated at a temperature of 27 °C. The benefit of this temperature for the formation of pupae is noted both at high (90%) and low (70%) humidity. Temperature limits above (32 °C) or below (22 °C) sharply reduces (1.5-2 times) the number of pupae formed. Maintaining an incubation microclimate with a humidity of 70% and a temperature of 27 ° C provides a large population of *Stomoxys* spp under laboratory settings, reaching 70% or more of the number of stock pupae available.

In addition, larval medium used in this study has an advantage over those utilized by Parr (13), Friesen, Berkebile (14) and Bailey, Whitfield (31). Initially, to induce egg laying a medium containing the following ingredients was prepared according to Parr (1959): 300g dried cow dung, 150g dried blood granules and 30g sugar. Eggs introduced into this medium demonstrated a meagre percentage of hatching, and those that did hatch did not survive the first instar stage. On reflection, it was hypothesized that dried dung and dried blood became sterile during the medium preparation procedure where ingredients were subjected to complete drying. This is in agreement with findings reported by (32-35) indicating that bacteria present in the natural substrate sustain the viability of stable flies throughout developmental stages. Moreover, the production expenditure is an essential consideration in stable fly’s maintenance program. Lack of some costly ingredients in the larval medium used such as a wheat barn, sugar cane bagasse, fishmeal and wood shavings and ammonium bicarbonate have shown no effect on the viability of immature *Stomoxys* flies reared in this study.

**Conclusion**

To study the biology of the reproduction of *Stomoxys* flies and the selection of the optimal microclimate for their breeding, seven microclimates were designed and tested, with different temperature conditions and air humidity. The most technologically advanced and productive microclimate was defined as combined mode. Using incubation in double-level microclimate have proven to be efficient at the level of production we have been maintaining.

**Acknowledgements**

The author is sincerely thankful to Mr Aslan Kerembaev and Ms Raihan Nissanova for technical assistance provided as well as research project # AP05135323 of the Science Committee/ Ministry of Education and Science/Republic of Kazakhstan for testing, led the design and transportation support.

**Conflict of interest**

The author declare that they have no conflict of interest.

**Bibliography**

1.	Baldacchino F, Muenworn V, Desquesnes M, Desoli F, Charoenviriyaphap T, Duvallet G. Transmission of pathogens by Stomoxys flies (Diptera, Muscidae): a review. Parasite (Paris, France). 2013;20:26-.

2.	Berkebile DR, Thomas GD, Campbell JB. OVERWINTERING OF THE STABLE FLY (DIPTERA, MUSCIDAE) IN SOUTHEASTERN NEBRASKA. Journal of Economic Entomology. 1994;87(6):1555-63.

3.	Lysyk TJ. SEASONAL ABUNDANCE OF STABLE FLIES AND HOUSE-FLIES (DIPTERA, MUSCIDAE) IN DAIRIES IN ALBERTA, CANADA. Journal of Medical Entomology. 1993;30(5):888-95.

4.	Campbell JB, Berry IL, Boxler DJ, Davis RL, Clanton DC, Deutscher GH. Effects of Stable Flies (Diptera: Muscidae) on Weight Gain and Feed Efficiency of Feedlot Cattle. Journal of Economic Entomology. 1987;80(1):117-9.

5.	Bruce WN, Decker GC. The relationship of stable fly abundance to milk production in dairy cattle. Jour Econ Ent. 1958;51((3)):268-74.

6.	Campbell JB, Skoda SR, Berkebile DR, Boxler DJ, Thomas GD, Adams DC, et al. Effects of stable flies (Diptera : Muscidae) on weight gains of grazing yearling cattle. Journal of Economic Entomology. 2001;94(3):780-3.

7.	Desquesnes M, Dia ML. Mechanical transmission of Trypanosoma vivax in cattle by the African tabanid Atylotus fuscipes. Vet Parasitol. 2004;119(1):9-19.

8.	Schuberg A, Boing W. On the transmission of diseases by indigenous biting insects

Ueber die Uebertragung von Krankheiten durch einheimische stechende Insekten. Arb Kaiserl Gesundheits. 1914;47(3):491-512 pp.

9.	Krinsky WL. Animal disease agents transmitted by horse flies and deer flies (Diptera: Tabanidae). Journal of Medical Entomology. 1976;13(3):224-75.

10.	Ferris DH, Hanson RP, Dicke RJ, Roberts RH. EXPERIMENTAL TRANSMISSION OF VESICULAR STOMATITIS VIRUS BY DIPTERA. J Infect Dis. 1955;96(2):184-92.

11.	Hoch AL, Gargan TP, Bailey CL. MECHANICAL TRANSMISSION OF RIFT-VALLEY FEVER VIRUS BY HEMATOPHAGOUS DIPTERA. Am J Trop Med Hyg. 1985;34(1):188-93.

12.	Issimov A, Kutumbetov L, Orynbayev MB, Khairullin B, Myrzakhmetova B, Sultankulova K, et al. Mechanical Transmission of Lumpy Skin Disease Virus by Stomoxys Spp (Stomoxys Calsitrans, Stomoxys Sitiens, Stomoxys Indica), Diptera: Muscidae. Animals : an open access journal from MDPI. 2020;10(3).

13.	Parr HCM. Studies on Stomoxys calcitrans (L.) in Uganda, East Africa.: I.—A Method of Rearing large Numbers of Stomoxys calcitrans. Bulletin of Entomological Research. 1959;50(1):165-9.

14.	Friesen K, Berkebile DR, Zhu JJ, Taylor DB. Laboratory Rearing of Stable Flies and Other Muscoid Diptera. J Vis Exp. 2018(138):7.

15.	Zumpt F. The Stomoxyine biting flies of the world. Diptera: Muscidae. Taxonomy, biology, economic importance and control measures1973. viii + 175 p.

16.	Masmeatathip R, Ketavan C, Duvallet G. Morphological studies of Stomoxys spp. (Diptera: Muscidae) in central Thailand. Kasetsart Journal Natural Sciences. 2006;40(4):872-81.

17.	Turell MJ, Knudson GB. Mechanical transmission of Bacillus anthracis by stable flies (Stomoxys calcitrans) and mosquitoes (Aedes aegypti and Aedes taeniorhynchus). Infection and immunity. 1987;55(8):1859-61.

18.	Mellor PS, Kitching RP, Wilkinson PJ. MECHANICAL TRANSMISSION OF CAPRIPOX VIRUS AND AFRICAN SWINE FEVER VIRUS BY STOMOXYS-CALCITRANS. Res Vet Sci. 1987;43(1):109-12.

19.	Turell MJ, Linthicum KJ, Beaman JR. TRANSMISSION OF RIFT-VALLEY FEVER VIRUS BY ADULT MOSQUITOS AFTER INGESTION OF VIRUS AS LARVAE. Am J Trop Med Hyg. 1990;43(6):677-80.

20.	Foil LD, Meek CL, Adams WV, Issel CJ. MECHANICAL TRANSMISSION OF EQUINE INFECTIOUS-ANEMIA VIRUS BY DEER FLIES (CHRYSOPS-FLAVIDUS) AND STABLE FLIES (STOMOXYS-CALCITRANS). Am J Vet Res. 1983;44(1):155-6.

21.	Chihota CM, Rennie LF, Kitching RP, Mellor PS. Mechanical transmission of lumpy skin disease virus by Aedes aegypti (Diptera: Culicidae). Epidemiology and Infection. 2001;126(2):317-21.

22.	Sohier C, Haegeman A, Mostin L, De Leeuw I, Campe WV, De Vleeschauwer A, et al. Experimental evidence of mechanical lumpy skin disease virus transmission by Stomoxys calcitrans biting flies and Haematopota spp. horseflies. Sci Rep. 2019;9(1):20076.

23.	Lysyk TJ. Relationships between temperature and life-history parameters of Stomoxys calcitrans (Diptera : Muscidae). Journal of Medical Entomology. 1998;35(2):107-19.

24.	Kunz SE, Berry IL, Foerster KW. DEVELOPMENT OF IMMATURE FORMS OF STOMOXYS-CALCITRANS DIPTERA-MUSCIDAE. Ann Entomol Soc Am. 1977;70(2):169-72.

25.	Sutherland B. SOME EFFECTS OF TEMPERATURE ON THE ADULTS, EGGS AND PUPAE OF STOMOXYS-CALCITRANS LINNAEUS (DIPTERA, MUSCIDAE). Onderstepoort Journal of Veterinary Research. 1979;46(4):223-7.

26.	McPheron LJ, Broce AB. Environmental components of pupariation-site selection by the stable fly (Diptera: Muscidae). Environ Entomol. 1996;25(3):665-71.

27.	Rasmussen RL, Campbell JB. Investigation of Environmental Factors and Their Relationship to Populations of the Stable Fly, Stomoxys calcitrans (L.) 12. Environ Entomol. 1981;10(5):798-800.

28.	Gilles J, David JF, Duvallet G. Effects of temperature on the rate of increase of Stomoxys calcitrans and Stomoxys niger niger (Diptera : muscidae) from La Reunion Island. Journal of Medical Entomology. 2005;42(6):959-65.

29.	Gilles J, David JF, Duvallet G. Temperature effects on development and survival of two stable flies, Stomoxys calcitrans and Stomoxys niger niger (Diptera : Muscidae), in La Reunion Island. Journal of Medical Entomology. 2005;42(3):260-5.

30.	Cruz-Vazquez C, Mendoza IV, Parra MR, Garcia-Vazquez Z. Influence of temperature, humidity and rainfall on field population trend of Stomoxys calcitrans (Diptera: Muscidae) in a semiarid climate in Mexico. Parasitologia Latinoamericana. 2004;59(3-4):99-103.

31.	Bailey DL, Whitfield TL, Labrecque GC. LABORATORY BIOLOGY AND TECHNIQUES FOR MASS PRODUCING STABLE FLY, STOMOXYS-CALCITRANS (L) (DIPTERA-MUSCIDAE). Journal of Medical Entomology. 1975;12(2):189-93.

32.	Romero A, Broce A, Zurek L. Role of bacteria in the oviposition behaviour and larval development of stable flies. Med Vet Entomol. 2006;20(1):115-21.

33.	Lysyk TJ, Kalischuk-Tymensen L, Selinger LB, Lancaster RC, Wever L, Cheng KJ. Rearing stable fly larvae (Diptera : Muscidae) on an egg yolk medium. Journal of Medical Entomology. 1999;36(3):382-8.

34.	Albuquerque TA, Zurek L. Temporal changes in the bacterial community of animal feces and their correlation with stable fly oviposition, larval development, and adult fitness. Front Microbiol. 2014;5:9.

35.	Talley J, Broce A, Zurek L. Characterization of Stable Fly (Diptera: Muscidae) Larval Developmental Habitat at Round Hay Bale Feeding Sites. Journal of Medical Entomology. 2009;46(6):1310-9.
