# Supplementary material for: The combined effects of temperature and relative humidity parameters on the reproduction of Stomoxys species in a laboratory setting
Source: PLoS One. 2020 Dec 21;15(12):e0242794. doi: 10.1371/journal.pone.0242794 (PMC7751869; doi:10.1371/journal.pone.0242794)
Supplement: Attachment — Submitted filename: Response to Reviewers.docx [file pone.0242794.s004.docx]

Response to Reviewer 1 Comments

**Point 1: Line 135**  The fresh manure mixed with hay was collected from the insect secured animal facility of RIBSP to eliminate any contamination by other insects. (Despite years of sampling, I have never seen stable fly larvae developing in pure, fresh bovine feces)

**Response 1:** Sorry for misinterpretation, here I meant approximately 2 days old manure. The word “fresh manure” has been translated directly from my language where it is still fresh until dries off completely and become light brown.

**Point 2: Lines 144-146** The mean time period required for completion of each developmental stage was not extended or reduced specifically to obtain a high yield of immature flies and was determined according to Friesen, Berkebile (14). What does this mean?

**Response 2:** In this sentence, I wanted to mention that incubation time for each stage was not extended or reduced to get high numbers of flies. Instead, I used a time interval suggested by Parr and Friesen to keep the process as simple as possible. For example, the larva was collected 24 h post oviposition, pupa 10-13 days after oviposition.

**Point 3: Line 149-153** Statistical analysis was conducted using Tukey-Kramer multiple comparisons test in SAS 9.4 (Proc GLIMMIX, Cary, NC, USA)to define mean values and significant differences in numbers of species hatched, pupated and emerged using different environmental settings. In what? Size? Number?

**Response 3:** In Numbers

**Point 4:** **Line 272** How is this reference relevant to this statement?

**Response 4:** Revised as requested

**Point 5:** **Line 278** This reference discusses % moisture in substrate, not RH. Need to indicate how this relates.

**Response 5:** Revised as requested.

**Point 6:** **Lines 312-313** Temp above 32 and below 22 were not evaluated in this study.

**Response 6:** Revised as requested.

**Point 7:** **Lines 317-319** Data are needed to support this paragraph

**Response 7:** Revised as requested.

**Point 8:** Lines 327-330 Data?

**Response 8:** Revised as requested.

**Point 9:** Lines 334-335 ??

**Response 9:** Revised as requested.
